# Binary charge-transfer complexes using pyromellitic acid dianhydride featuring C—H⋯O hydrogen bonds

**DOI:** 10.1107/S2056989018015645

**Published:** 2018-11-09

**Authors:** Tania N. Hill, Andreas Lemmerer

**Affiliations:** a Molecular Sciences Institute, School of Chemistry, University of the Witwatersrand, Private Bag, PO WITS, 2050, Johannesburg, South Africa

**Keywords:** crystal structure, charge transfer, Hirshfeld surface, hydrogen bonding

## Abstract

Four binary charge-transfer complexes were made using pyromellitic acid dianhydride (pmda), those being pmda–naphthalene (1/1), C_10_H_2_O_6_·C_10_H_8_, (I), pmda–fluoranthene (1/1), C_10_H_2_O_6_·C_16_H_10_, (II), pmda–9-methyl­anthracene (1/1), C_10_H_2_O_6_·C_15_H_12_, (III), and pmda–ethyl anthracene-9-carboxyl­ate (1/2), C_10_H_2_O_6_·2C_17_H_12_O_3_, (IV). All charge-transfer complexes show alternating donor and acceptor stacks, which have weak C—H⋯O hydrogen bonds connecting the donor and acceptor mol­ecules. In addition, complex (I) has *Z*′ = 1/2, complex (II) has a *Z*′ = 2 and complex (IV) has half mol­ecule of pyromellitic acid dianhydride in the asymmetric unit.

## Abstract 2

Four binary charge-transfer complexes were made using pyromellitic acid dianhydride (pmda), all of which show alternating donor and acceptor stacks, which have weak C—H⋯O hydrogen bonds connecting the donor and acceptor mol­ecules.

## Chemical context   

Crystal engineering, the conception and synthesis of mol­ecular solid state structures, is fundamentally based upon the discernment and subsequent exploitation of inter­molecular inter­actions. Consequently, non-covalent bonding inter­actions are primarily used to achieve the organization of mol­ecules and ions in the solid state in order to produce materials with desired properties. and this understanding using a variety of inter­molecular inter­actions is at the very heart of crystal engineering. Recently, it has been shown that one can synthesize supra­molecular assemblies that contain anywhere from three to six different mol­ecular moieties (Paul *et al.*, 2018 ▸). Supra­molecular synthesis chiefly uses the hydrogen-bond inter­action as the most directional of the known inter­molecular inter­actions (Aakeröy & Beatty, 2001 ▸). An equally important inter­action is that of charge transfer (CT) between an electron-rich π-system (donor) and an electron-poor π-system (acceptor) (Herbstein, 2005 ▸). Classic donor mol­ecules (polycyclic aromatic hydro­carbons) generally have an electron-rich π-system. On the other hand, aromatic hydro­carbons with strongly polarizing groups, such as 1,3,5-tri­nitro­benzene (TNB), have an electron-poor π-system and are classified as the acceptor mol­ecule (Hill *et al.*, 2018*a* ▸,*b* ▸). Another common acceptor mol­ecule is pyromellitic acid dianhydride (pmda), which has electron-withdrawing O atoms of the carb­oxy­lic acid dianhydride groups. (pmda)·(pyrene) complexes have been investigated for order–disorder transitions as a function of temperature using infrared and Raman spectroscopy (Isaac *et al.*, 2018 ▸), (pmda)·(naphthalene) has been studied *via* Raman spectroscopy for having orientational disorder (Macfarlane & Ushioda, 1977 ▸), disorder in (pmda)·(perylene) *via* computer simulation (Boeyens & Levendis, 1986 ▸), and photoconductivity and magentoconductance in pmda·(pyrene) (Kato *et al.*, 2017 ▸). To this end, we have synthesized four new charge-transfer co-crystals that show no disorder: (pmda)·(naphthalene) (I), (pmda)·(fluoranthene) (II), (pmda)·(9-methyl­anthracene) (III), and (pmda)_2_·(9-ethyl ester anthracene) (IV).
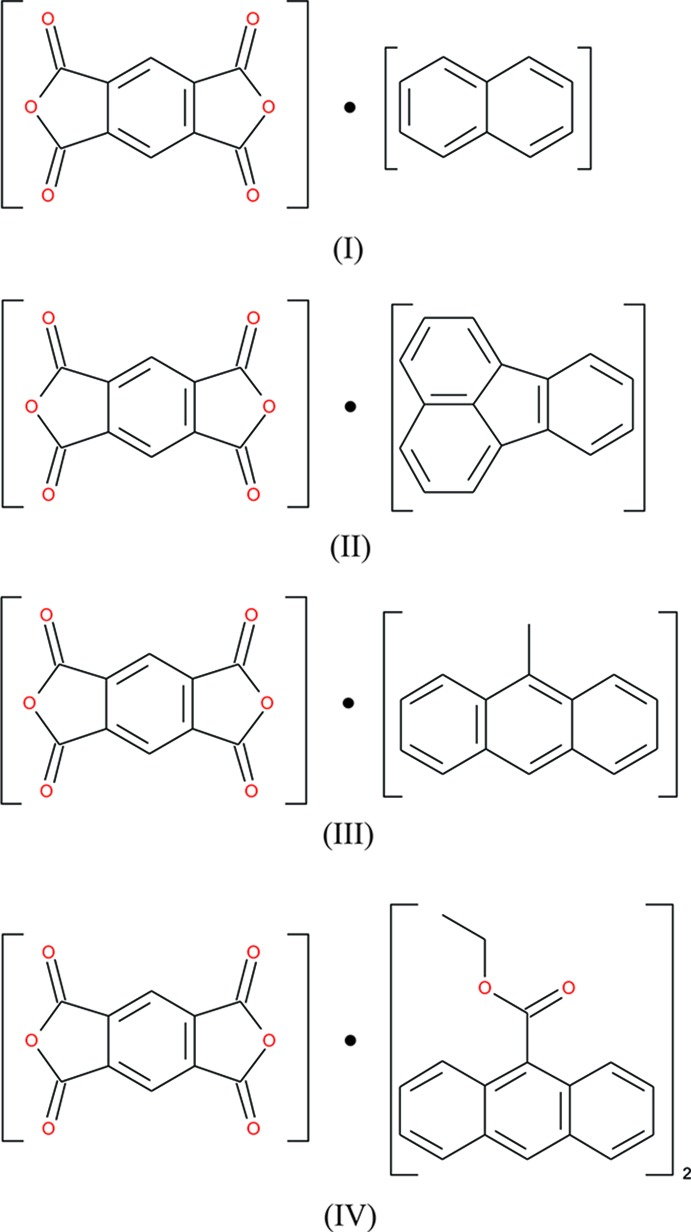


## Structural commentary   

**Figure 1:**
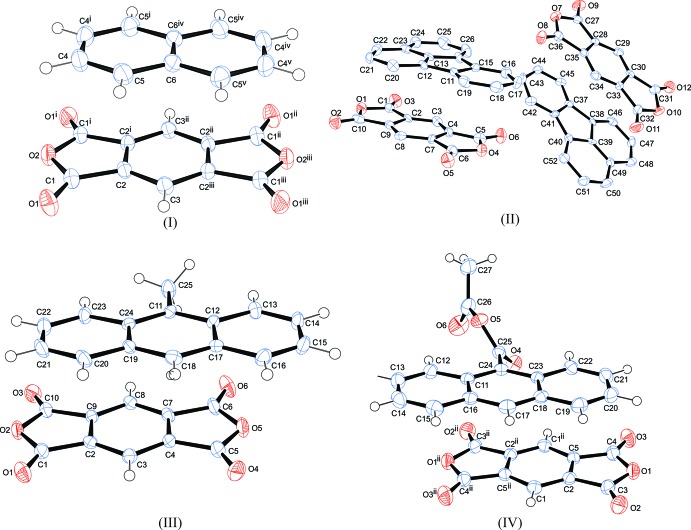
Perspective views of compounds (I)–(IV), showing the atom-numbering schemes. Displacement ellipsoids are drawn at the 50% probability level and H atoms are shown as small spheres of arbitrary radii. [Symmetry codes: (i) *x*, 1 − *y*, *z*; (ii) −*x*, 1 − *y*, 1 − *z*; (iii) −*x*, *y*, 1 − *z*; (iv) −*x*, 1 − *y*, −*z*; (v) −*x*, *y*, −*z*; (vi) *x* − 1, *y* − 1, *z* − 1.]

**Figure 2:**
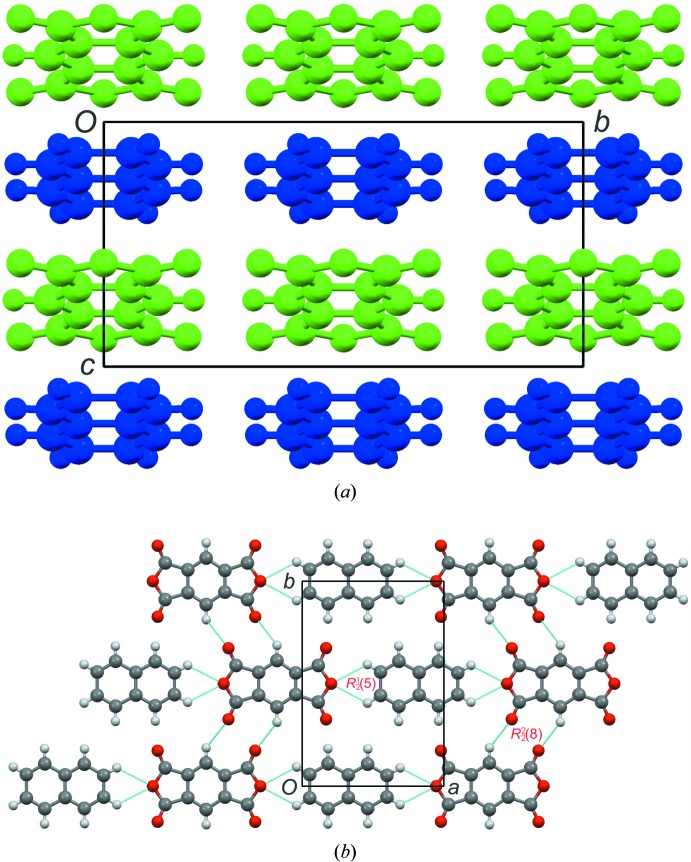
(*a*) A packing diagram of (I) showing the layers of donor (blue) and acceptor (green) mol­ecules. (*b*) Hydrogen-bonding diagram for (I) showing the C—H⋯O hydrogen-bonded rings formed between the pmda and naphthalene mol­ecules.

The asymmetric units and atom-labelling schemes are shown in Fig. 1 ▸, together with their displacement ellipsoids, for all charge-transfer complexes. As a result of the strong polarizing effect of the carb­oxy­lic acid dianhydride groups, pmda has an electron-poor π-system and functions as an acceptor. On the other side, the donor mol­ecules comprising polycyclic aromatic hydro­carbons have an electron-rich π-system. The packing of the mol­ecules of the four complexes follows a donor (*D*) acceptor (*A*) π–π inter­action, which is the major driving force in the formation of these complexes, as seen in Figs. 2 ▸ and 3 ▸ (donor mol­ecules shown in blue/yellow and acceptor in green/red), resulting in a general face-to-face π-stacking, with Table 1 ▸ summarizing the closest centroid–centroid distances between the pmda acceptor and aromatic donor systems. The inter­molecular inter­actions of the *D*⋯*A* stacks can be qu­anti­fied using Hirshfeld surface analysis as well as the resulting fingerprint plots using the programme *CrystalExplorer 17.5* (Spackman & McKinnon, 2002 ▸). Table 2 ▸ summarizes the percentages for all combinations of contacts between C, H and O atoms and the relevant fingerprint plots are given in the supporting information. In the paper by Chen *et al.* (2017 ▸), the authors describe that regions of blue and red triangles on the Hirshfeld surface using the shape index as evidence of π–π inter­actions. Fig. 4 ▸ shows such surfaces plotted for the pmda mol­ecules in (I)–(IV), and for comparison the shape index of the pmda mol­ecule in its unimolecular crystal structure. The red triangles show concave regions indicative of ring carbons of the π stacked mol­ecule above it. Complexes (I)–(IV) display a high number of triangles, which reveals the increased proportion of π–π stacking observed for the four structures.. The shape index of pmda shows no such pattern [Fig. 4 ▸(*a*)]. This π stacking can be qu­anti­fied by looking at the contribution of the C⋯C contacts contained in the fingerprint plots, which vary only slightly from 19.9 to 21.0%. The greatest contribution to the Hirshfeld surfaces are seen in the H⋯O contacts, which vary from 48.5 to 58.4%. In comparison, the C⋯C contacts only make up 0.2% in pmda⋯pmda and the C⋯O contacts have the greatest single contribution at 43%. In summary, the introduction of an aromatic polycylic changes the biggest contributor from C⋯O in pmda to H⋯O in pmda-aromatic polycyclics.

## Supra­molecular features   

Compound (I) crystallizes in the *C*2/m space group with one quarter of the pmda and naphthalene mol­ecules occupying a twofold axis and a mirror plane, resulting in Z′ = 0.25 for the asymmetric unit. The donor and acceptor mol­ecules stack along the *c*-axis direction, and in a checker board fashion along the *ab* plane [Fig. 2 ▸(*a*)]. In the direction of the *a*-axis, there is a symmetrical C4—H4⋯O2 inter­action from both ends of the naphthalene mol­ecule to the oxygen atoms on the pmda [Fig. 2 ▸(*b*), Table 3 ▸]. As a result of the mirror plane symmetry, this results in a very symmetrical 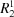
(5) ring as described using graph-set notation (Bernstein *et al.*, 1995 ▸). Along the *b*-axis, there is an additional hydrogen bonded ring, 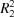
(8), resulting from C3—H3⋯O1 hydrogen-bond inter­action [Fig. 2 ▸(*b*)].

**Figure 3:**
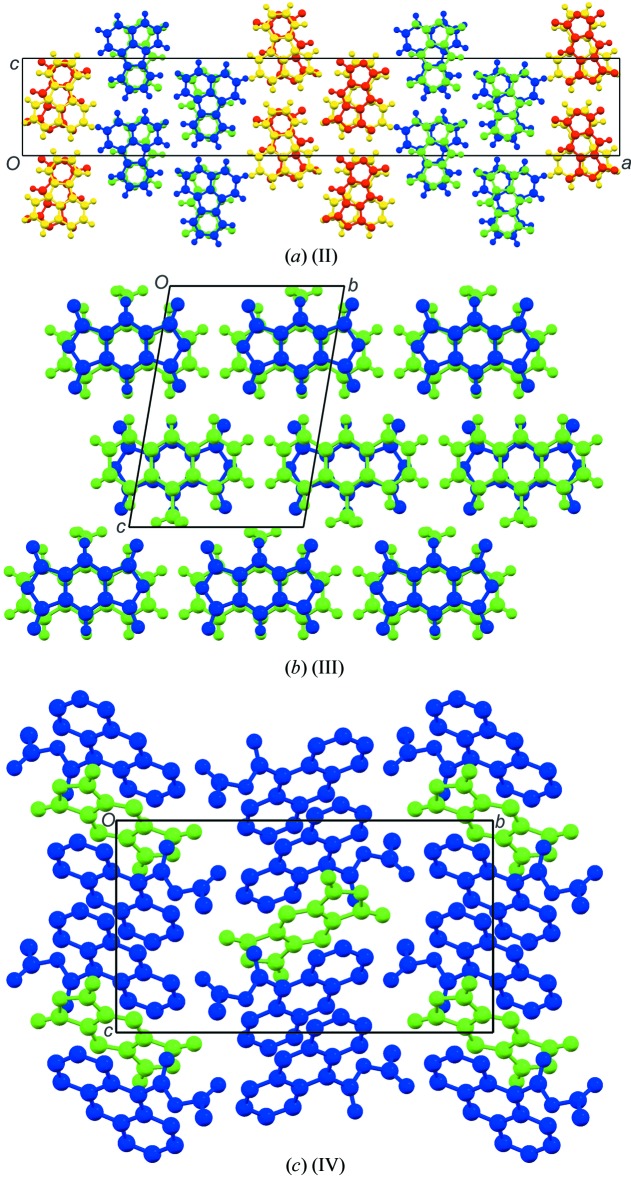
Packing diagrams for (II)–(IV). The donor mol­ecules are shown in blue or yellow, and the acceptor mol­ecules in green or red.

**Figure 4:**
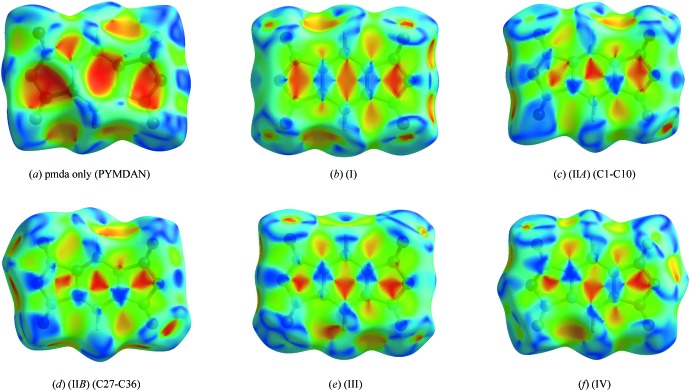
The mol­ecular Hirshfeld surfaces mapped over shape index for the pmda mol­ecule by itself (PYMDAN) and for the pmda acceptor mol­ecule in charge transfer complexes (I)–(IV).

**Figure 5:**
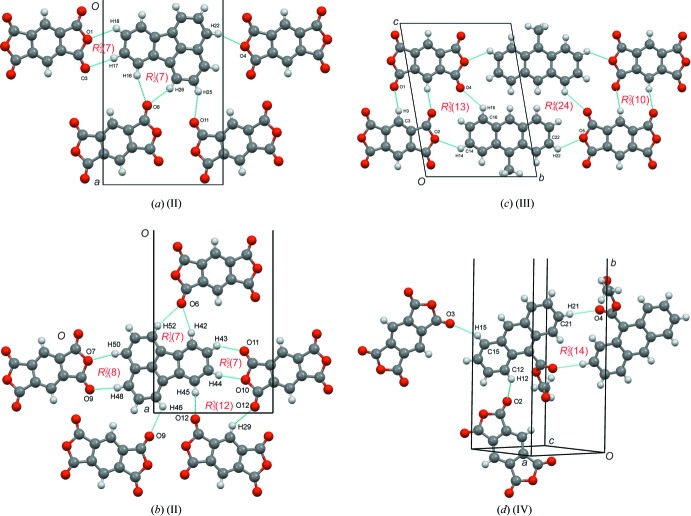
Hydrogen-bonding diagrams for (II)–(IV). Atom labels correspond to those given in the hydrogen-bonding tables.

Compound (II) crystallizes in the *P*ca2_1_ space group with two pmda and two fluoranthene mol­ecules in the asymmetric unit. One set of *D*/*A* pairs is shown in blue/green, and the second is shown in yellow/red. The separation of the two *D*/*A* pairs can be clearly seen in Fig. 3 ▸(*a*). Between the four unique pmda acceptor and fluoranthene donors there are numerous C—H⋯O inter­actions (Table 4 ▸). As the fluoranthene has only C and H atoms, it is the mol­ecule that has the most weak hydrogen-bond donor groups (C—H), and the pmda, with six oxygen atoms, has numerous good hydrogen-bond acceptor atoms (O). Fig. 5 ▸(*a*) and 5(*b*) illustrate four of the hydrogen bonds emanating from the two symmetry-independent fluoranthene mol­ecules, which form a number of hydrogen-bonded rings: 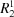
(7), 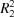
(7), 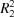
(8) and 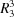
(12).

Compound (III) crystallizes in the *P* 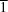 space group with both the pmda and 9-methyl­anthracene in the asymmetric unit. The packing of the structure shows the typical donor–acceptor stacking along the *a* axis [Fig. 3 ▸(*b*)] and has the closest centroid-to-centroid distance of all four charge-transfer complexes at 3.2994 (4) Å (Table 1 ▸). Perpendicular to the stacking axis, the donor and acceptor mol­ecules form hydrogen-bonded layers using four distinct C—H⋯O hydrogen bonds (Table 5 ▸). The combination of these individually or in groups results in three types of hydrogen bonded rings, 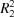
(10), 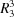
(13) and 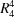
(24), shown in Fig. 5 ▸(*c*).

Compound (IV) crystallizes in the *P*2_1_/*c* space group with half a pmda (on a centre of inversion) and one complete 9-ethyl ester anthracene mol­ecule in the asymmetric unit, giving a ratio of one acceptor to two donors. [Fig. 3 ▸(*c*)]. Two donor mol­ecules form a hydrogen-bonded ring dimer [Fig. 5 ▸(*d*)], graph-set 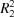
(14), *via* a C21—H21⋯O4 hydrogen bond Two pmda mol­ecules are connected to the donor *via* discrete hydrogen bonds C12—H12⋯O2 and C15—H15⋯O3 (Table 6 ▸).

One of the major differences between the four complexes is the symmetry of the asymmetric unit. Pmda, being a very symmetrical mol­ecule with point group *D*_2h_, is shown to crystallize with *Z*′ = 0.25, 0.5 and 1 in the title complexes. In the literature, the most common case is with *Z*′ = 0.5, such as those with anthracene (ANTPML; Boeyens & Herbstein, 1965 ▸; ANTPML01 and ANTPML01; Robertson & Stezowski, 1978 ▸), acridine (BIWVUY; Karl *et al.*, 1982*b* ▸), bi­phenyl­ene (DURZAR, DURZAR01, DURZAR02; Stezowski *et al.*, 1986 ▸), chrysene (FILHIR; Bulgarovskaya *et al.*, 1987*b* ▸) to name but a few. More unusual is the case with *Z*′ = 0.25, seen only twice in 9,10-di­bromo­anthracene (FILHEN; Bulgarovskaya *et al.*, 1987*a* ▸) and naphthalene (NAPYMA01; Le Bars-Combe *et al.*, 1979 ▸). It has also been observed were pmda is present with both *Z*′ = 0.5 and 1, such as in RUYWIR (Kurebayashi *et al.*, 2001 ▸), 3,6-di­bromo­carbazole (VILFIF; Bulgarovskaya *et al.*, 1989 ▸) and *N*-methyl-3,6-di­bromo­carbazole (WEXKEP; Dzyabchenko *et al.*, 1994 ▸). In summary, we have characterized a further new set of four CT complexes of pmda and aromatic mol­ecules.

## Database survey   

A database survey in the Cambridge Structural Database (CSD, Version 5.39; November 2017 update; Groom *et al.*, 2016 ▸) was undertaken for any structures containing the pmda moiety. A total of 26 complexes were found, four showing polymorphism [BECNUS02 (Karl *et al.*, 1982*a* ▸) and BECNUS10 (Bugarovskaya *et al.*, 1982 ▸); DURZAR and DURZAR01 (Stezowski *et al.*, 1986 ▸); NAPYMA01 (Le Bars-Combe *et al.*, 1979 ▸) and NAPYMA12 (Le Bars-Combe *et al.*, 1981 ▸); PYRPMA04 (Herbstein *et al.*, 1994 ▸) and PYRPMA11 (Kato *et al.*, 2017 ▸)] and one showing stoichiometric variation [VILFEB and VILFIF (Bulgarovskaya *et al.*, 1989 ▸)].

## Synthesis and crystallization   

All chemicals were purchased from commercial sources (Sigma Aldrich) and used as received without further purification. The pyromellitic acid dianhydride charge transfer complexes were prepared in a 10 mL ethano­lic solution with a 1:1 stoichiometric ratio of the donor to the acceptor mol­ecule which was then heated and stirred until total dissolution took place (approx. 4 h). The solution was then cooled very slowly and allowed to evaporate to obtain crystals suitable for X-ray diffraction. Detailed masses are as follows: (I): 0.100 g of pyromellitic acid dianhydride and 0.059 g of naphthalene; (II): 0.100 g of pyromellitic acid dianhydride and 0.093 g of fluoranthene; (III): 0.100 g of pyromellitic acid dianhydride and 0.088 g of 9-methyl­anthracene; and (IV): 0.100 g of pyromellitic acid dianhydride and 0.12 1 g of 9-ethyl ester anthracene.

## Refinement details   

Crystal data, data collection and structure refinement details are summarized in Table 7 ▸. For all compounds, the C-bound H atoms were geometrically placed (C—H bond lengths of 0.96 (methyl CH_3_), and 0.95 (Ar—H) Å) and refined as riding with *U*_iso_(H) = 1.2*U*_eq_(Ar-C) or *U*_iso_(H) = 1.5*U*_eq_(methyl-C).

## Supplementary Material

Crystal structure: contains datablock(s) I, II, III, IV, shelx. DOI: 10.1107/S2056989018015645/eb2013sup1.cif

Structure factors: contains datablock(s) I. DOI: 10.1107/S2056989018015645/eb2013Isup2.hkl

Structure factors: contains datablock(s) II. DOI: 10.1107/S2056989018015645/eb2013IIsup3.hkl

Structure factors: contains datablock(s) III. DOI: 10.1107/S2056989018015645/eb2013IIIsup4.hkl

Structure factors: contains datablock(s) IV. DOI: 10.1107/S2056989018015645/eb2013IVsup5.hkl

Fingerprint plots for all five compounds mentioned in the text. DOI: 10.1107/S2056989018015645/eb2013sup6.pdf

CCDC references: 1877153, 1877152, 1877151, 1877150

Additional supporting information:  crystallographic information; 3D view; checkCIF report

## Figures and Tables

**Table 1:** Centroid distances (Å) between the pmda and the ring centroids (*Cg*) of the aromatic polycyclics

Structure	Acceptor *Cg*	Donor *Cg*	*Cg*⋯*Cg*	Symmetry Operator
(I)	C1–O1 (*Cg*3)	C4–C6 (*Cg*6)	3.3724 (2)	−*x* + 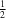 , *y* − 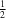 , −*z*
(II)	O1–C10 (*Cg*5)	C11–C19 (*Cg*14)	3.3193 (5)	*x*, *y*, *z*
(III)	C2–C9 (*Cg*3)	C11–C24 (*Cg*10)	3.2994 (4)	*x* − 1, *y*, *z*
(IV)	C1–O1 (*Cg*9)	C11–C24 (*Cg*3)	3.3280 (3)	1 − *x*, −*y*, 1 − *z*

**Table 2:** Proportion (%) of inter­molecular contacts between donor and acceptor (pmda) mol­ecules in the Hirshfeld fingerprint plots

Structure	C⋯C	H⋯H	C⋯H	O⋯O	O⋯H	C⋯O
(pmda)	0.2	8.0	1.0	29.9	17.9	43.0
(I)	19.8	6.6	3.9	9.5	58.4	1.7
(II*A*)	21.0	8.6	5.4	5.5	52.8	6.6
(II*B*)	20.6	11.7	6.2	7.1	48.5	5.9
(III)	20.2	9.5	4.1	4.2	56.8	5.2
(IV)	20.9	10.8	2.7	4.4	53.9	7.3

**Table 3:** Hydrogen-bond geometry (Å, °) for (I)

*D*—H⋯*A*	*D*—H	H⋯*A*	*D*⋯*A*	*D*—H⋯*A*
C4—H4⋯O2^i^	0.95	2.68	3.2748 (14)	121
C3—H3⋯O1^ii^	0.95	2.63	3.3463 (13)	133
C5—H5⋯O1^ii^	0.95	2.69	3.4127 (14)	133

Symmetry codes: (i) 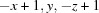; (ii) 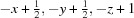.

**Table 4:** Hydrogen-bond geometry (Å, °) for (II)

*D*—H⋯*A*	*D*—H	H⋯*A*	*D*⋯*A*	*D*—H⋯*A*
C8—H8⋯O2^i^	0.95	2.67	3.373 (5)	132
C16—H16⋯O8	0.95	2.59	3.444 (5)	150
C17—H17⋯O3^ii^	0.95	2.65	3.576 (5)	166
C18—H18⋯O1^ii^	0.95	2.67	3.332 (5)	127
C22—H22⋯O4^iii^	0.95	2.59	3.481 (5)	155
C25—H25⋯O11^iii^	0.95	2.55	3.347 (5)	142
C29—H29⋯O12^iv^	0.95	2.71	3.370 (5)	127
C42—H42⋯O6	0.95	2.49	3.413 (5)	165
C43—H43⋯O11^iii^	0.95	2.58	3.293 (5)	132
C44—H44⋯O10^iii^	0.95	2.52	3.428 (5)	160
C45—H45⋯O12^iv^	0.95	2.57	3.429 (5)	150
C46—H46⋯O9^v^	0.95	2.64	3.256 (5)	123
C48—H48⋯O9^vi^	0.95	2.55	3.473 (5)	164
C50—H50⋯O7^vi^	0.95	2.5	3.420 (5)	164
C52—H52⋯O6	0.95	2.62	3.525 (5)	159

Symmetry codes: (i) 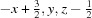; (ii) 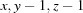; (iii) 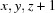; (iv) 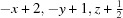; (v) 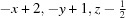; (vi) 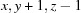.

**Table 5:** Hydrogen-bond geometry (Å, °) for (III)

*D*—H⋯*A*	*D*—H	H⋯*A*	*D*⋯*A*	*D*—H⋯*A*
C3—H3⋯O1^i^	0.95	2.55	3.376 (4)	145
C14—H14⋯O2^ii^	0.95	2.63	3.347 (4)	133
C16—H16⋯O4^iii^	0.95	2.68	3.365 (4)	130
C22—H22⋯O5^iv^	0.95	2.64	3.323 (4)	130

Symmetry codes: (i) 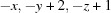; (ii) 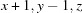; (iii) 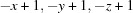; (iv) 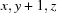.

**Table 6:** Hydrogen-bond geometry (Å, °) for (IV)

*D*—H⋯*A*	*D*—H	H⋯*A*	*D*⋯*A*	*D*—H⋯*A*
C12—H12⋯O2^i^	0.95	2.65	3.351 (2)	131
C15—H15⋯O3^ii^	0.95	2.55	3.306 (2)	137
C21—H21⋯O4^iii^	0.95	2.48	3.433 (2)	176

Symmetry codes: (i) 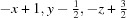; (ii) 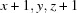; (iii) 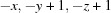.

**Table 7:** Experimental details

	(I)	(II)	(III)	(IV)
Crystal data				
Chemical formula	C_10_H_2_O_6_·C_10_H_8_	C_10_H_2_O_6_·C_16_H_10_	C_10_H_2_O_6_·C_15_H_12_	C_17_H_12_O_3_·0.5C_10_H_2_O_6_
*M*_r_	346.28	420.36	410.36	373.32
Crystal system, space group	Monoclinic, *C*2/*m*	Orthorhombic, *P* *c* *a*2_1_	Triclinic, *P* 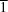	Monoclinic, *P*2_1_/*c*
Temperature (K)	173	173	173	173
*a*, *b*, *c* (Å)	9.1478 (4), 12.8195 (6), 6.7459 (3)	57.356 (9), 7.0172 (10), 9.3429 (13)	7.1012 (8), 9.5674 (12), 13.6147 (16)	9.1949 (7), 17.9751 (14), 10.9716 (10)
α, β, γ (°)	90, 104.202 (3), 90	90, 90, 90	99.109 (4), 99.941 (4), 92.219 (4)	90, 112.829 (2), 90
*V* (Å^3^)	766.91 (6)	3760.3 (9)	897.53 (19)	1671.3 (2)
*Z*	2	8	2	4
Radiation type	Mo *K*α	Mo *K*α	Mo *K*α	Mo *K*α
μ (mm^−1^)	0.11	0.11	0.11	0.11
Crystal size (mm)	0.40 × 0.08 × 0.05	0.5 × 0.1 × 0.1	0.19 × 0.06 × 0.05	0.55 × 0.1 × 0.06

Data collection				
Diffractometer	Bruker D8 Venture Photon CCD area detector	Bruker D8 Venture Photon CCD area detector	Bruker D8 Venture Photon CCD area detector	Bruker D8 Venture Photon CCD area detector
Absorption correction	Multi-scan *SADABS* (Krause *et al.*, 2015 ▸)	Multi-scan *SADABS* (Krause *et al.*, 2015 ▸)	Multi-scan *SADABS* (Krause *et al.*, 2015 ▸)	Multi-scan *SADABS* (Krause *et al.*, 2015 ▸)
*T*_min_, *T*_max_	0.9, 0.95	0.9, 0.95	0.9, 0.95	0.9, 0.95
No. of measured, independent and observed [*I* > 2σ(*I*)] reflections	3774, 967, 841	40403, 6983, 5636	20202, 3280, 2159	13071, 4035, 2731
*R*_int_	0.042	0.054	0.075	0.046

Refinement				
*R*[*F* ^2^ > 2σ(*F* ^2^)], *wR*(*F* ^2^), *S*	0.034, 0.098, 1.04	0.045, 0.106, 1.08	0.073, 0.223, 1.02	0.043, 0.112, 1.05
No. of reflections	967	6983	3280	4035
No. of parameters	63	577	281	254
No. of restraints	0	1	0	0
H-atom treatment	H-atom parameters constrained	H-atom parameters constrained	H-atom parameters constrained	H-atom parameters constrained
Δρ_max_, Δρ_min_ (e Å^−3^)	0.36, −0.3	0.21, −0.17	0.67, −0.28	0.30, −0.26

Computer programs: *APEX3*, *SAINT-Plus* and *XPREP* (Bruker 2016 ▸), *SHELXS97* (Sheldrick, 2008 ▸), *SHELXL2017/1* (Sheldrick, 2015 ▸), *ORTEP-3 for Windows* and *WinGX* publication routines (Farrugia, 2012 ▸) and *Mercury* (Macrae *et al.*, 2006 ▸).

## supplementary crystallographic information

### Pyromellitic acid dianhydride–naphthalene (1/1) (I) Crystal data

**Table:**

C_10_H_2_O_6_·C_10_H_8_	*F*(000) = 356
*M**_r_* = 346.28	*D*_x_ = 1.5 Mg m^−^^3^
Monoclinic, *C*2/*m*	Mo *K*α radiation, λ = 0.71073 Å
Hall symbol: -C 2y	Cell parameters from 2019 reflections
*a* = 9.1478 (4) Å	θ = 2.8–28.2°
*b* = 12.8195 (6) Å	µ = 0.11 mm^−^^1^
*c* = 6.7459 (3) Å	*T* = 173 K
β = 104.202 (3)°	Plate, yellow
*V* = 766.91 (6) Å^3^	0.40 × 0.08 × 0.05 mm
*Z* = 2	

### Pyromellitic acid dianhydride–naphthalene (1/1) (I) Data collection

**Table:**

Bruker D8 Venture Photon CCD area detector diffractometer	841 reflections with *I* > 2σ(*I*)
Graphite monochromator	*R*_int_ = 0.042
ω scans	θ_max_ = 28.0°, θ_min_ = 2.8°
Absorption correction: multi-scan (SADABS; Krause *et al.*, 2015)	*h* = −11→12
*T*_min_ = 0.9, *T*_max_ = 0.95	*k* = −16→16
3774 measured reflections	*l* = −8→8
967 independent reflections	

### Pyromellitic acid dianhydride–naphthalene (1/1) (I) Refinement

**Table:**

Refinement on *F*^2^	Hydrogen site location: mixed
Least-squares matrix: full	H-atom parameters constrained
*R*[*F*^2^ > 2σ(*F*^2^)] = 0.034	*w* = 1/[σ^2^(*F*_o_^2^) + (0.0541*P*)^2^ + 0.2771*P*] where *P* = (*F*_o_^2^ + 2*F*_c_^2^)/3
*wR*(*F*^2^) = 0.098	(Δ/σ)_max_ < 0.001
*S* = 1.04	Δρ_max_ = 0.36 e Å^−^^3^
967 reflections	Δρ_min_ = −0.3 e Å^−^^3^
63 parameters	Extinction correction: SHELXL-2017/1 (Sheldrick 2015), Fc^*^=kFc[1+0.001xFc^2^λ^3^/sin(2θ)]^-1/4^
0 restraints	Extinction coefficient: 0.011 (3)
0 constraints	

### Pyromellitic acid dianhydride–naphthalene (1/1) (I) Special details

**Table:**

Experimental. Absorption corrections were made using the program SADABS (Sheldrick, 1996)
Geometry. All esds (except the esd in the dihedral angle between two l.s. planes) are estimated using the full covariance matrix. The cell esds are taken into account individually in the estimation of esds in distances, angles and torsion angles; correlations between esds in cell parameters are only used when they are defined by crystal symmetry. An approximate (isotropic) treatment of cell esds is used for estimating esds involving l.s. planes.

### Pyromellitic acid dianhydride–naphthalene (1/1) (I) Fractional atomic coordinates and isotropic or equivalent isotropic displacement parameters (Å^2^)

**Table:**

	*x*	*y*	*z*	*U*_iso_*/*U*_eq_	
C1	0.29033 (11)	0.41071 (8)	0.62113 (16)	0.0267 (3)	
C2	0.13074 (10)	0.44565 (8)	0.55335 (13)	0.0221 (3)	
C3	0	0.38652 (11)	0.5	0.0238 (3)	
H3	0	0.312417	0.5	0.029*	
O1	0.34466 (8)	0.32627 (6)	0.65041 (12)	0.0379 (3)	
O2	0.37963 (11)	0.5	0.65364 (16)	0.0309 (3)	
C4	0.27282 (12)	0.44493 (10)	0.10439 (16)	0.0358 (3)	
H4	0.365478	0.407881	0.140233	0.043*	
C5	0.13998 (12)	0.39113 (10)	0.05322 (16)	0.0324 (3)	
H5	0.141317	0.317033	0.053253	0.039*	
C6	0	0.44465 (12)	0	0.0262 (3)	

### Pyromellitic acid dianhydride–naphthalene (1/1) (I) Atomic displacement parameters (Å^2^)

**Table:**

	*U*^11^	*U*^22^	*U*^33^	*U*^12^	*U*^13^	*U*^23^
C1	0.0203 (5)	0.0307 (6)	0.0273 (5)	0.0019 (4)	0.0023 (4)	−0.0027 (4)
C2	0.0187 (5)	0.0248 (5)	0.0219 (5)	0.0025 (3)	0.0032 (3)	0.0002 (3)
C3	0.0225 (6)	0.0209 (6)	0.0263 (7)	0	0.0030 (5)	0
O1	0.0283 (4)	0.0329 (5)	0.0476 (5)	0.0107 (3)	0.0000 (3)	−0.0026 (3)
O2	0.0181 (5)	0.0337 (6)	0.0382 (6)	0	0.0017 (4)	0
C4	0.0221 (5)	0.0545 (7)	0.0289 (6)	0.0073 (5)	0.0029 (4)	0.0021 (5)
C5	0.0294 (6)	0.0377 (6)	0.0295 (5)	0.0060 (4)	0.0059 (4)	0.0020 (4)
C6	0.0224 (6)	0.0353 (8)	0.0204 (6)	0	0.0042 (5)	0

### Pyromellitic acid dianhydride–naphthalene (1/1) (I) Geometric parameters (Å, º)

**Table:**

C1—O1	1.1872 (12)	C4—C5	1.3659 (16)
C1—O2	1.3921 (12)	C4—C4^i^	1.412 (3)
C1—C2	1.4878 (12)	C4—H4	0.9497
C2—C3	1.3868 (12)	C5—C6	1.4190 (13)
C2—C2^i^	1.394 (2)	C5—H5	0.95
C3—H3	0.9499	C6—C6^ii^	1.419 (3)
			
O1—C1—O2	121.20 (9)	C5—C4—C4^i^	120.33 (7)
O1—C1—C2	131.66 (10)	C5—C4—H4	119.7
O2—C1—C2	107.13 (8)	C4^i^—C4—H4	120
C3—C2—C2^i^	123.13 (6)	C4—C5—C6	120.76 (12)
C3—C2—C1	129.34 (9)	C4—C5—H5	119.6
C2^i^—C2—C1	107.52 (6)	C6—C5—H5	119.6
C2—C3—C2^iii^	113.73 (12)	C5—C6—C5^iv^	122.18 (15)
C2—C3—H3	123.1	C5—C6—C6^ii^	118.91 (7)
C2^iii^—C3—H3	123.1	C5^iv^—C6—C6^ii^	118.91 (7)
C1^i^—O2—C1	110.63 (11)		
			
O1—C1—C2—C3	−1.92 (18)	O1—C1—O2—C1^i^	−176.38 (6)
O2—C1—C2—C3	179.21 (8)	C2—C1—O2—C1^i^	2.63 (15)
O1—C1—C2—C2^i^	177.30 (11)	C4^i^—C4—C5—C6	−0.22 (11)
O2—C1—C2—C2^i^	−1.57 (9)	C4—C5—C6—C5^iv^	−179.78 (11)
C2^i^—C2—C3—C2^iii^	0	C4—C5—C6—C6^ii^	0.22 (11)
C1—C2—C3—C2^iii^	179.11 (11)		

Symmetry codes: (i) *x*, −*y*+1, *z*; (ii) −*x*, −*y*+1, −*z*; (iii) −*x*, *y*, −*z*+1; (iv) −*x*, *y*, −*z*.

### Pyromellitic acid dianhydride–naphthalene (1/1) (I) Hydrogen-bond geometry (Å, º)

**Table:**

*D*—H···*A*	*D*—H	H···*A*	*D*···*A*	*D*—H···*A*
C4—H4···O2^v^	0.95	2.68	3.2748 (14)	121
C3—H3···O1^vi^	0.95	2.63	3.3463 (13)	133
C5—H5···O1^vi^	0.95	2.69	3.4127 (14)	133

Symmetry codes: (v) −*x*+1, *y*, −*z*+1; (vi) −*x*+1/2, −*y*+1/2, −*z*+1.

### Pyromellitic acid dianhydride–fluoranthene (1/1) (II) Crystal data

**Table:**

C_10_H_2_O_6_·C_16_H_10_	*F*(000) = 1728
*M**_r_* = 420.36	*D*_x_ = 1.485 Mg m^−^^3^
Orthorhombic, *P**c**a*2_1_	Mo *K*α radiation, λ = 0.71073 Å
Hall symbol: P 2c -2ac	Cell parameters from 9936 reflections
*a* = 57.356 (9) Å	θ = 2.9–25.0°
*b* = 7.0172 (10) Å	µ = 0.11 mm^−^^1^
*c* = 9.3429 (13) Å	*T* = 173 K
*V* = 3760.3 (9) Å^3^	Needle, yellow
*Z* = 8	0.5 × 0.1 × 0.1 mm

### Pyromellitic acid dianhydride–fluoranthene (1/1) (II) Data collection

**Table:**

Bruker D8 Venture Photon CCD area detector diffractometer	5636 reflections with *I* > 2σ(*I*)
Graphite monochromator	*R*_int_ = 0.054
ω scans	θ_max_ = 25.5°, θ_min_ = 2.9°
Absorption correction: multi-scan (SADABS; Krause *et al.*, 2015)	*h* = −68→69
*T*_min_ = 0.9, *T*_max_ = 0.95	*k* = −8→8
40403 measured reflections	*l* = −11→11
6983 independent reflections	

### Pyromellitic acid dianhydride–fluoranthene (1/1) (II) Refinement

**Table:**

Refinement on *F*^2^	0 constraints
Least-squares matrix: full	Hydrogen site location: inferred from neighbouring sites
*R*[*F*^2^ > 2σ(*F*^2^)] = 0.045	H-atom parameters constrained
*wR*(*F*^2^) = 0.106	*w* = 1/[σ^2^(*F*_o_^2^) + (0.0572*P*)^2^ + 0.1599*P*] where *P* = (*F*_o_^2^ + 2*F*_c_^2^)/3
*S* = 1.08	(Δ/σ)_max_ = 0.002
6983 reflections	Δρ_max_ = 0.21 e Å^−^^3^
577 parameters	Δρ_min_ = −0.17 e Å^−^^3^
1 restraint	

### Pyromellitic acid dianhydride–fluoranthene (1/1) (II) Special details

**Table:**

Experimental. Absorption corrections were made using the program SADABS (Sheldrick, 1996)
Geometry. All esds (except the esd in the dihedral angle between two l.s. planes) are estimated using the full covariance matrix. The cell esds are taken into account individually in the estimation of esds in distances, angles and torsion angles; correlations between esds in cell parameters are only used when they are defined by crystal symmetry. An approximate (isotropic) treatment of cell esds is used for estimating esds involving l.s. planes.

### Pyromellitic acid dianhydride–fluoranthene (1/1) (II) Fractional atomic coordinates and isotropic or equivalent isotropic displacement parameters (Å^2^)

**Table:**

	*x*	*y*	*z*	*U*_iso_*/*U*_eq_	
C1	0.82357 (7)	1.1250 (5)	0.7955 (4)	0.0292 (9)	
C2	0.81768 (6)	1.0608 (5)	0.6489 (4)	0.0222 (9)	
C3	0.83230 (7)	1.0187 (5)	0.5355 (4)	0.0247 (9)	
H3	0.848791	1.028283	0.541393	0.03*	
C4	0.82087 (6)	0.9617 (5)	0.4134 (4)	0.0223 (8)	
C5	0.83073 (7)	0.9076 (5)	0.2727 (4)	0.0274 (9)	
C6	0.79114 (7)	0.8856 (5)	0.2571 (4)	0.0265 (9)	
C7	0.79692 (6)	0.9479 (4)	0.4036 (4)	0.0211 (9)	
C8	0.78227 (6)	0.9916 (5)	0.5173 (4)	0.0239 (8)	
H8	0.765778	0.98281	0.510836	0.029*	
C9	0.79363 (6)	1.0491 (5)	0.6408 (4)	0.0237 (9)	
C10	0.78389 (7)	1.1076 (5)	0.7808 (4)	0.0263 (9)	
O1	0.80273 (5)	1.1492 (3)	0.8698 (3)	0.0313 (6)	
O2	0.76441 (5)	1.1242 (4)	0.8216 (3)	0.0367 (7)	
O3	0.84189 (5)	1.1546 (4)	0.8516 (3)	0.0398 (7)	
O4	0.81209 (4)	0.8637 (3)	0.1825 (3)	0.0296 (6)	
O5	0.77309 (5)	0.8529 (4)	0.1999 (3)	0.0362 (7)	
O6	0.85035 (5)	0.8965 (4)	0.2327 (3)	0.0373 (7)	
C11	0.79962 (7)	0.4589 (4)	0.4321 (4)	0.0220 (9)	
C12	0.79336 (6)	0.5209 (5)	0.5780 (4)	0.0216 (8)	
C13	0.81464 (6)	0.5575 (5)	0.6486 (4)	0.0209 (8)	
C14	0.83381 (6)	0.5247 (5)	0.5586 (4)	0.0247 (9)	
C15	0.82429 (6)	0.4634 (5)	0.4204 (4)	0.0235 (9)	
C16	0.83500 (7)	0.4131 (5)	0.2922 (4)	0.0292 (9)	
H16	0.851483	0.416417	0.282892	0.035*	
C17	0.82101 (7)	0.3579 (5)	0.1783 (4)	0.0324 (10)	
H17	0.828098	0.323881	0.09003	0.039*	
C18	0.79686 (7)	0.3513 (5)	0.1905 (4)	0.0309 (10)	
H18	0.787731	0.311898	0.110994	0.037*	
C19	0.78599 (7)	0.4014 (5)	0.3169 (4)	0.0271 (9)	
H19	0.769492	0.396593	0.325054	0.032*	
C20	0.77293 (6)	0.5517 (5)	0.6511 (4)	0.0270 (9)	
H20	0.758286	0.52863	0.606837	0.032*	
C21	0.77417 (7)	0.6187 (5)	0.7942 (4)	0.0281 (9)	
H21	0.760053	0.640805	0.844686	0.034*	
C22	0.79487 (6)	0.6527 (5)	0.8623 (4)	0.0266 (9)	
H22	0.794862	0.696499	0.958518	0.032*	
C23	0.81630 (6)	0.6229 (5)	0.7904 (4)	0.0252 (9)	
C24	0.83898 (7)	0.6531 (5)	0.8429 (5)	0.0316 (9)	
H24	0.841192	0.695894	0.938428	0.038*	
C25	0.85813 (7)	0.6204 (5)	0.7558 (5)	0.0357 (10)	
H25	0.873296	0.643029	0.792923	0.043*	
C26	0.85582 (7)	0.5545 (5)	0.6131 (5)	0.0331 (10)	
H26	0.869228	0.531161	0.55609	0.04*	
C27	0.95132 (7)	0.2701 (5)	0.3916 (4)	0.0273 (9)	
C28	0.94915 (6)	0.3286 (4)	0.2407 (4)	0.0211 (8)	
C29	0.96640 (6)	0.3586 (5)	0.1395 (4)	0.0240 (9)	
H29	0.982522	0.342393	0.159271	0.029*	
C30	0.95802 (6)	0.4145 (4)	0.0070 (4)	0.0214 (8)	
C31	0.97109 (7)	0.4574 (5)	−0.1257 (5)	0.0284 (9)	
C32	0.93229 (7)	0.5019 (5)	−0.1718 (4)	0.0268 (9)	
C33	0.93452 (7)	0.4410 (5)	−0.0213 (4)	0.0236 (9)	
C34	0.91744 (6)	0.4095 (5)	0.0794 (4)	0.0247 (9)	
H34	0.901313	0.425949	0.059588	0.03*	
C35	0.92565 (6)	0.3521 (5)	0.2117 (4)	0.0239 (9)	
C36	0.91269 (7)	0.3065 (5)	0.3450 (5)	0.0299 (9)	
O7	0.92884 (4)	0.2590 (3)	0.4485 (3)	0.0315 (7)	
O8	0.89225 (5)	0.3043 (4)	0.3676 (3)	0.0396 (7)	
O9	0.96799 (5)	0.2355 (4)	0.4632 (3)	0.0396 (7)	
O10	0.95479 (5)	0.5088 (3)	−0.2297 (3)	0.0318 (7)	
O11	0.91570 (5)	0.5429 (4)	−0.2403 (3)	0.0382 (7)	
O12	0.99143 (5)	0.4552 (4)	−0.1519 (3)	0.0370 (7)	
C37	0.94594 (6)	0.8366 (4)	0.2153 (4)	0.0224 (8)	
C38	0.95615 (6)	0.8863 (4)	0.0751 (4)	0.0212 (8)	
C39	0.93732 (6)	0.9377 (5)	−0.0149 (4)	0.0224 (9)	
C40	0.91574 (6)	0.9237 (5)	0.0587 (4)	0.0215 (8)	
C41	0.92138 (6)	0.8593 (5)	0.2046 (4)	0.0218 (8)	
C42	0.90731 (7)	0.8204 (5)	0.3214 (4)	0.0277 (9)	
H42	0.890861	0.833772	0.315028	0.033*	
C43	0.91770 (7)	0.7615 (5)	0.4480 (4)	0.0309 (10)	
H43	0.908251	0.734899	0.52909	0.037*	
C44	0.94165 (7)	0.7410 (5)	0.4578 (4)	0.0302 (10)	
H44	0.948373	0.701203	0.545763	0.036*	
C45	0.95597 (7)	0.7771 (5)	0.3420 (4)	0.0261 (9)	
H45	0.972381	0.761441	0.34947	0.031*	
C46	0.97819 (7)	0.8917 (5)	0.0193 (4)	0.0278 (9)	
H46	0.991274	0.855935	0.075564	0.033*	
C47	0.98106 (7)	0.9521 (5)	−0.1249 (4)	0.0303 (9)	
H47	0.996364	0.956701	−0.16371	0.036*	
C48	0.96282 (7)	1.0037 (5)	−0.2099 (4)	0.0296 (10)	
H48	0.965573	1.043925	−0.305523	0.036*	
C49	0.93963 (6)	0.9974 (4)	−0.1556 (4)	0.0240 (9)	
C50	0.91870 (7)	1.0472 (5)	−0.2278 (5)	0.0322 (10)	
H50	0.919178	1.088363	−0.32461	0.039*	
C51	0.89775 (7)	1.0355 (5)	−0.1570 (4)	0.0296 (9)	
H51	0.883939	1.071225	−0.206259	0.036*	
C52	0.89595 (7)	0.9724 (5)	−0.0137 (4)	0.0286 (9)	
H52	0.88115	0.96405	0.031533	0.034*	

### Pyromellitic acid dianhydride–fluoranthene (1/1) (II) Atomic displacement parameters (Å^2^)

**Table:**

	*U*^11^	*U*^22^	*U*^33^	*U*^12^	*U*^13^	*U*^23^
C1	0.035 (2)	0.023 (2)	0.030 (2)	0.0012 (17)	0.000 (2)	−0.0011 (17)
C2	0.025 (2)	0.0174 (18)	0.024 (2)	0.0030 (15)	0.0004 (18)	−0.0001 (15)
C3	0.023 (2)	0.0215 (19)	0.029 (2)	0.0009 (16)	0.0007 (18)	−0.0023 (16)
C4	0.028 (2)	0.0169 (17)	0.022 (2)	0.0022 (16)	0.0019 (18)	−0.0020 (15)
C5	0.032 (3)	0.0204 (19)	0.030 (2)	0.0005 (16)	0.003 (2)	−0.0028 (17)
C6	0.029 (2)	0.0221 (19)	0.029 (2)	−0.0001 (16)	0.0037 (19)	−0.0039 (18)
C7	0.026 (2)	0.0146 (17)	0.023 (2)	−0.0004 (15)	−0.0003 (18)	−0.0023 (15)
C8	0.025 (2)	0.0201 (17)	0.026 (2)	−0.0004 (16)	0.0009 (19)	0.0016 (15)
C9	0.028 (2)	0.0165 (18)	0.027 (2)	0.0021 (15)	−0.0001 (18)	0.0005 (16)
C10	0.033 (2)	0.0182 (18)	0.028 (2)	−0.0013 (16)	0.004 (2)	0.0002 (16)
O1	0.0394 (17)	0.0318 (14)	0.0226 (16)	0.0010 (12)	0.0035 (14)	−0.0063 (12)
O2	0.0370 (18)	0.0329 (15)	0.0402 (19)	−0.0011 (12)	0.0127 (14)	−0.0046 (13)
O3	0.0395 (18)	0.0430 (16)	0.0368 (18)	0.0043 (13)	−0.0064 (16)	−0.0115 (14)
O4	0.0320 (16)	0.0330 (15)	0.0238 (15)	0.0015 (12)	0.0020 (13)	−0.0062 (12)
O5	0.0347 (17)	0.0394 (15)	0.0345 (17)	−0.0004 (13)	−0.0071 (15)	−0.0096 (13)
O6	0.0295 (17)	0.0480 (17)	0.0344 (18)	0.0026 (13)	0.0100 (14)	−0.0077 (14)
C11	0.028 (2)	0.0155 (17)	0.022 (2)	0.0036 (16)	0.0003 (17)	0.0015 (15)
C12	0.026 (2)	0.0149 (17)	0.024 (2)	0.0005 (15)	−0.0016 (18)	−0.0015 (15)
C13	0.026 (2)	0.0155 (18)	0.021 (2)	−0.0001 (15)	0.0011 (17)	−0.0002 (15)
C14	0.027 (2)	0.0196 (19)	0.027 (2)	0.0003 (15)	0.0003 (18)	−0.0037 (16)
C15	0.028 (2)	0.0188 (18)	0.024 (2)	0.0010 (16)	0.0018 (18)	0.0012 (15)
C16	0.036 (2)	0.0242 (19)	0.028 (2)	−0.0002 (17)	0.006 (2)	−0.0025 (16)
C17	0.053 (3)	0.026 (2)	0.018 (2)	0.0076 (19)	0.011 (2)	−0.0013 (17)
C18	0.047 (3)	0.025 (2)	0.021 (2)	0.0057 (18)	−0.004 (2)	0.0012 (16)
C19	0.032 (2)	0.0209 (18)	0.029 (2)	0.0016 (16)	−0.0035 (19)	−0.0018 (16)
C20	0.029 (2)	0.0215 (19)	0.030 (2)	0.0009 (16)	0.0007 (19)	−0.0017 (16)
C21	0.036 (2)	0.0237 (19)	0.025 (2)	0.0019 (17)	0.0102 (19)	0.0005 (16)
C22	0.036 (2)	0.0245 (19)	0.019 (2)	0.0001 (16)	0.0040 (19)	−0.0038 (16)
C23	0.033 (2)	0.0147 (17)	0.028 (2)	0.0022 (15)	−0.0033 (19)	0.0006 (16)
C24	0.038 (2)	0.028 (2)	0.029 (2)	−0.0019 (17)	−0.004 (2)	−0.0036 (17)
C25	0.028 (2)	0.035 (2)	0.044 (3)	−0.0008 (18)	−0.005 (2)	−0.007 (2)
C26	0.027 (2)	0.031 (2)	0.042 (3)	0.0008 (17)	−0.001 (2)	−0.0073 (19)
C27	0.038 (2)	0.0209 (19)	0.023 (2)	−0.0012 (17)	0.001 (2)	0.0014 (16)
C28	0.027 (2)	0.0154 (17)	0.021 (2)	−0.0006 (15)	0.0009 (17)	−0.0011 (15)
C29	0.025 (2)	0.0205 (18)	0.027 (2)	0.0029 (15)	−0.0039 (17)	0.0029 (16)
C30	0.025 (2)	0.0145 (17)	0.024 (2)	−0.0008 (15)	0.0027 (17)	−0.0032 (15)
C31	0.035 (3)	0.0181 (18)	0.032 (2)	0.0003 (16)	0.001 (2)	0.0007 (17)
C32	0.036 (2)	0.023 (2)	0.022 (2)	0.0023 (17)	0.000 (2)	0.0015 (16)
C33	0.032 (2)	0.0182 (18)	0.021 (2)	0.0026 (16)	0.0001 (18)	0.0014 (15)
C34	0.026 (2)	0.0203 (19)	0.028 (2)	0.0018 (16)	−0.0001 (19)	−0.0007 (16)
C35	0.030 (2)	0.0162 (17)	0.025 (2)	0.0009 (15)	0.0039 (18)	−0.0007 (15)
C36	0.036 (2)	0.0209 (19)	0.033 (2)	0.0020 (17)	0.005 (2)	0.0030 (17)
O7	0.0381 (16)	0.0324 (15)	0.0241 (16)	0.0020 (12)	0.0034 (13)	0.0066 (12)
O8	0.0348 (17)	0.0429 (16)	0.0411 (19)	0.0011 (13)	0.0147 (15)	0.0044 (14)
O9	0.0449 (18)	0.0443 (17)	0.0295 (16)	0.0003 (14)	−0.0095 (15)	0.0090 (14)
O10	0.0377 (17)	0.0334 (14)	0.0241 (15)	0.0025 (12)	0.0060 (14)	0.0084 (12)
O11	0.0432 (18)	0.0424 (16)	0.0291 (17)	0.0034 (13)	−0.0054 (15)	0.0109 (14)
O12	0.0331 (17)	0.0369 (15)	0.0410 (18)	0.0003 (12)	0.0123 (15)	0.0048 (13)
C37	0.029 (2)	0.0138 (16)	0.024 (2)	−0.0023 (15)	0.0005 (18)	−0.0011 (14)
C38	0.026 (2)	0.0124 (17)	0.025 (2)	−0.0031 (14)	−0.0004 (18)	−0.0006 (15)
C39	0.027 (2)	0.0136 (17)	0.027 (2)	−0.0017 (14)	−0.0008 (18)	0.0003 (15)
C40	0.029 (2)	0.0169 (18)	0.019 (2)	−0.0039 (15)	0.0007 (17)	−0.0004 (15)
C41	0.029 (2)	0.0148 (17)	0.021 (2)	−0.0038 (15)	0.0013 (17)	0.0006 (14)
C42	0.031 (2)	0.0230 (19)	0.029 (2)	−0.0005 (16)	0.0037 (19)	0.0020 (16)
C43	0.041 (3)	0.027 (2)	0.024 (2)	−0.0029 (17)	0.007 (2)	0.0011 (17)
C44	0.049 (3)	0.0235 (19)	0.018 (2)	−0.0020 (18)	−0.005 (2)	0.0035 (15)
C45	0.033 (2)	0.0187 (17)	0.027 (2)	−0.0014 (16)	−0.009 (2)	0.0002 (16)
C46	0.026 (2)	0.0207 (18)	0.036 (2)	−0.0011 (16)	−0.0002 (19)	−0.0007 (17)
C47	0.029 (2)	0.0282 (19)	0.034 (3)	−0.0030 (17)	0.011 (2)	−0.0004 (17)
C48	0.041 (3)	0.0218 (19)	0.026 (2)	−0.0064 (17)	0.010 (2)	0.0024 (16)
C49	0.031 (2)	0.0177 (18)	0.023 (2)	−0.0028 (15)	0.0043 (19)	−0.0026 (16)
C50	0.047 (3)	0.027 (2)	0.022 (2)	−0.0032 (18)	−0.001 (2)	0.0065 (17)
C51	0.030 (2)	0.033 (2)	0.026 (2)	0.0011 (17)	−0.0052 (19)	0.0033 (18)
C52	0.029 (2)	0.028 (2)	0.029 (2)	−0.0021 (16)	0.0013 (18)	0.0018 (17)

### Pyromellitic acid dianhydride–fluoranthene (1/1) (II) Geometric parameters (Å, º)

**Table:**

C1—O3	1.193 (5)	C27—O9	1.192 (4)
C1—O1	1.393 (5)	C27—O7	1.397 (4)
C1—C2	1.481 (6)	C27—C28	1.474 (5)
C2—C3	1.383 (5)	C28—C29	1.384 (5)
C2—C9	1.384 (5)	C28—C35	1.385 (5)
C3—C4	1.375 (5)	C29—C30	1.385 (5)
C3—H3	0.95	C29—H29	0.95
C4—C7	1.380 (5)	C30—C33	1.386 (5)
C4—C5	1.480 (5)	C30—C31	1.479 (5)
C5—O6	1.188 (4)	C31—O12	1.193 (4)
C5—O4	1.396 (4)	C31—O10	1.396 (5)
C6—O5	1.188 (4)	C32—O11	1.182 (4)
C6—O4	1.397 (4)	C32—O10	1.400 (5)
C6—C7	1.474 (5)	C32—C33	1.475 (5)
C7—C8	1.389 (5)	C33—C34	1.376 (5)
C8—C9	1.385 (5)	C34—C35	1.383 (5)
C8—H8	0.95	C34—H34	0.95
C9—C10	1.480 (5)	C35—C36	1.485 (5)
C10—O2	1.186 (4)	C36—O8	1.191 (4)
C10—O1	1.395 (5)	C36—O7	1.380 (5)
C11—C19	1.390 (5)	C37—C45	1.381 (5)
C11—C15	1.420 (5)	C37—C41	1.421 (5)
C11—C12	1.475 (5)	C37—C38	1.476 (5)
C12—C20	1.374 (5)	C38—C46	1.368 (5)
C12—C13	1.411 (5)	C38—C39	1.416 (5)
C13—C14	1.403 (5)	C39—C49	1.386 (5)
C13—C23	1.405 (5)	C39—C40	1.419 (5)
C14—C26	1.378 (5)	C40—C52	1.365 (5)
C14—C15	1.466 (5)	C40—C41	1.472 (5)
C15—C16	1.391 (5)	C41—C42	1.385 (5)
C16—C17	1.388 (5)	C42—C43	1.387 (5)
C16—H16	0.95	C42—H42	0.95
C17—C18	1.391 (5)	C43—C44	1.384 (5)
C17—H17	0.95	C43—H43	0.95
C18—C19	1.381 (5)	C44—C45	1.381 (5)
C18—H18	0.95	C44—H44	0.95
C19—H19	0.95	C45—H45	0.95
C20—C21	1.419 (6)	C46—C47	1.422 (6)
C20—H20	0.95	C46—H46	0.95
C21—C22	1.368 (5)	C47—C48	1.362 (5)
C21—H21	0.95	C47—H47	0.95
C22—C23	1.416 (5)	C48—C49	1.424 (5)
C22—H22	0.95	C48—H48	0.95
C23—C24	1.407 (5)	C49—C50	1.421 (5)
C24—C25	1.386 (6)	C50—C51	1.374 (5)
C24—H24	0.95	C50—H50	0.95
C25—C26	1.417 (6)	C51—C52	1.413 (6)
C25—H25	0.95	C51—H51	0.95
C26—H26	0.95	C52—H52	0.95
			
O3—C1—O1	121.1 (4)	O9—C27—O7	121.0 (3)
O3—C1—C2	131.3 (4)	O9—C27—C28	131.4 (4)
O1—C1—C2	107.6 (3)	O7—C27—C28	107.6 (3)
C3—C2—C9	123.3 (4)	C29—C28—C35	123.0 (3)
C3—C2—C1	129.4 (3)	C29—C28—C27	129.4 (4)
C9—C2—C1	107.2 (3)	C35—C28—C27	107.6 (3)
C4—C3—C2	114.1 (3)	C28—C29—C30	113.9 (3)
C4—C3—H3	122.9	C28—C29—H29	123
C2—C3—H3	122.9	C30—C29—H29	123
C3—C4—C7	123.4 (3)	C29—C30—C33	123.0 (4)
C3—C4—C5	129.0 (3)	C29—C30—C31	129.1 (3)
C7—C4—C5	107.7 (3)	C33—C30—C31	107.8 (3)
O6—C5—O4	121.4 (4)	O12—C31—O10	121.0 (4)
O6—C5—C4	131.2 (4)	O12—C31—C30	131.7 (4)
O4—C5—C4	107.5 (3)	O10—C31—C30	107.3 (3)
O5—C6—O4	120.3 (4)	O11—C32—O10	121.6 (4)
O5—C6—C7	132.1 (4)	O11—C32—C33	131.0 (4)
O4—C6—C7	107.6 (3)	O10—C32—C33	107.4 (3)
C4—C7—C8	122.4 (3)	C34—C33—C30	122.7 (4)
C4—C7—C6	107.8 (3)	C34—C33—C32	129.5 (4)
C8—C7—C6	129.8 (3)	C30—C33—C32	107.7 (3)
C9—C8—C7	114.7 (3)	C33—C34—C35	114.5 (3)
C9—C8—H8	122.7	C33—C34—H34	122.7
C7—C8—H8	122.7	C35—C34—H34	122.7
C2—C9—C8	122.1 (4)	C34—C35—C28	122.8 (3)
C2—C9—C10	108.1 (3)	C34—C35—C36	129.9 (3)
C8—C9—C10	129.8 (3)	C28—C35—C36	107.3 (3)
O2—C10—O1	121.2 (4)	O8—C36—O7	122.2 (4)
O2—C10—C9	131.8 (4)	O8—C36—C35	130.1 (4)
O1—C10—C9	107.0 (3)	O7—C36—C35	107.7 (3)
C1—O1—C10	110.0 (3)	C36—O7—C27	109.8 (3)
C5—O4—C6	109.4 (3)	C31—O10—C32	109.8 (3)
C19—C11—C15	120.5 (3)	C45—C37—C41	120.5 (3)
C19—C11—C12	131.6 (4)	C45—C37—C38	131.8 (3)
C15—C11—C12	107.9 (3)	C41—C37—C38	107.7 (3)
C20—C12—C13	118.5 (4)	C46—C38—C39	118.1 (4)
C20—C12—C11	135.5 (4)	C46—C38—C37	135.3 (4)
C13—C12—C11	106.0 (3)	C39—C38—C37	106.6 (3)
C14—C13—C23	124.5 (3)	C49—C39—C38	124.6 (3)
C14—C13—C12	111.5 (3)	C49—C39—C40	124.3 (3)
C23—C13—C12	124.0 (3)	C38—C39—C40	111.1 (3)
C26—C14—C13	118.1 (4)	C52—C40—C39	117.9 (3)
C26—C14—C15	135.4 (4)	C52—C40—C41	136.0 (4)
C13—C14—C15	106.5 (3)	C39—C40—C41	106.2 (3)
C16—C15—C11	120.0 (3)	C42—C41—C37	120.0 (3)
C16—C15—C14	131.9 (4)	C42—C41—C40	131.5 (3)
C11—C15—C14	108.1 (3)	C37—C41—C40	108.5 (3)
C17—C16—C15	118.4 (4)	C41—C42—C43	118.7 (3)
C17—C16—H16	120.8	C41—C42—H42	120.6
C15—C16—H16	120.8	C43—C42—H42	120.6
C16—C17—C18	121.5 (4)	C44—C43—C42	120.9 (4)
C16—C17—H17	119.3	C44—C43—H43	119.6
C18—C17—H17	119.3	C42—C43—H43	119.6
C19—C18—C17	120.7 (4)	C45—C44—C43	121.3 (4)
C19—C18—H18	119.6	C45—C44—H44	119.4
C17—C18—H18	119.6	C43—C44—H44	119.4
C18—C19—C11	118.8 (4)	C37—C45—C44	118.6 (3)
C18—C19—H19	120.6	C37—C45—H45	120.7
C11—C19—H19	120.6	C44—C45—H45	120.7
C12—C20—C21	118.6 (4)	C38—C46—C47	118.5 (4)
C12—C20—H20	120.7	C38—C46—H46	120.8
C21—C20—H20	120.7	C47—C46—H46	120.8
C22—C21—C20	122.7 (4)	C48—C47—C46	122.8 (4)
C22—C21—H21	118.7	C48—C47—H47	118.6
C20—C21—H21	118.7	C46—C47—H47	118.6
C21—C22—C23	120.4 (4)	C47—C48—C49	120.1 (4)
C21—C22—H22	119.8	C47—C48—H48	120
C23—C22—H22	119.8	C49—C48—H48	120
C13—C23—C24	116.1 (4)	C39—C49—C50	116.3 (3)
C13—C23—C22	115.9 (3)	C39—C49—C48	115.9 (4)
C24—C23—C22	128.0 (4)	C50—C49—C48	127.8 (4)
C25—C24—C23	120.2 (4)	C51—C50—C49	119.7 (4)
C25—C24—H24	119.9	C51—C50—H50	120.1
C23—C24—H24	119.9	C49—C50—H50	120.1
C24—C25—C26	122.2 (4)	C50—C51—C52	122.6 (4)
C24—C25—H25	118.9	C50—C51—H51	118.7
C26—C25—H25	118.9	C52—C51—H51	118.7
C14—C26—C25	118.9 (4)	C40—C52—C51	119.1 (4)
C14—C26—H26	120.6	C40—C52—H52	120.4
C25—C26—H26	120.6	C51—C52—H52	120.4
			
O3—C1—C2—C3	−0.5 (7)	O9—C27—C28—C29	0.3 (6)
O1—C1—C2—C3	179.7 (3)	O7—C27—C28—C29	−179.9 (3)
O3—C1—C2—C9	179.8 (4)	O9—C27—C28—C35	−179.4 (4)
O1—C1—C2—C9	0.0 (4)	O7—C27—C28—C35	0.4 (4)
C9—C2—C3—C4	−0.7 (5)	C35—C28—C29—C30	0.1 (5)
C1—C2—C3—C4	179.7 (3)	C27—C28—C29—C30	−179.6 (3)
C2—C3—C4—C7	0.2 (5)	C28—C29—C30—C33	1.0 (5)
C2—C3—C4—C5	179.4 (3)	C28—C29—C30—C31	−179.5 (3)
C3—C4—C5—O6	1.8 (7)	C29—C30—C31—O12	0.0 (7)
C7—C4—C5—O6	−178.9 (4)	C33—C30—C31—O12	179.5 (4)
C3—C4—C5—O4	−179.0 (3)	C29—C30—C31—O10	−179.5 (3)
C7—C4—C5—O4	0.2 (4)	C33—C30—C31—O10	0.0 (4)
C3—C4—C7—C8	0.2 (5)	C29—C30—C33—C34	−1.6 (5)
C5—C4—C7—C8	−179.1 (3)	C31—C30—C33—C34	178.8 (3)
C3—C4—C7—C6	179.2 (3)	C29—C30—C33—C32	179.4 (3)
C5—C4—C7—C6	−0.1 (4)	C31—C30—C33—C32	−0.2 (4)
O5—C6—C7—C4	179.2 (4)	O11—C32—C33—C34	2.4 (7)
O4—C6—C7—C4	−0.1 (4)	O10—C32—C33—C34	−178.6 (3)
O5—C6—C7—C8	−1.9 (7)	O11—C32—C33—C30	−178.7 (4)
O4—C6—C7—C8	178.8 (3)	O10—C32—C33—C30	0.3 (4)
C4—C7—C8—C9	−0.3 (5)	C30—C33—C34—C35	1.0 (5)
C6—C7—C8—C9	−179.0 (3)	C32—C33—C34—C35	179.7 (3)
C3—C2—C9—C8	0.7 (5)	C33—C34—C35—C28	0.2 (5)
C1—C2—C9—C8	−179.6 (3)	C33—C34—C35—C36	179.6 (3)
C3—C2—C9—C10	−179.0 (3)	C29—C28—C35—C34	−0.7 (5)
C1—C2—C9—C10	0.7 (4)	C27—C28—C35—C34	179.0 (3)
C7—C8—C9—C2	−0.2 (5)	C29—C28—C35—C36	179.8 (3)
C7—C8—C9—C10	179.4 (3)	C27—C28—C35—C36	−0.5 (4)
C2—C9—C10—O2	177.8 (4)	C34—C35—C36—O8	2.1 (7)
C8—C9—C10—O2	−1.9 (6)	C28—C35—C36—O8	−178.4 (4)
C2—C9—C10—O1	−1.2 (4)	C34—C35—C36—O7	−179.1 (3)
C8—C9—C10—O1	179.2 (3)	C28—C35—C36—O7	0.4 (4)
O3—C1—O1—C10	179.4 (3)	O8—C36—O7—C27	178.7 (3)
C2—C1—O1—C10	−0.8 (4)	C35—C36—O7—C27	−0.2 (4)
O2—C10—O1—C1	−177.9 (3)	O9—C27—O7—C36	179.7 (3)
C9—C10—O1—C1	1.2 (4)	C28—C27—O7—C36	−0.1 (4)
O6—C5—O4—C6	179.0 (3)	O12—C31—O10—C32	−179.4 (3)
C4—C5—O4—C6	−0.3 (4)	C30—C31—O10—C32	0.2 (4)
O5—C6—O4—C5	−179.1 (3)	O11—C32—O10—C31	178.8 (3)
C7—C6—O4—C5	0.2 (4)	C33—C32—O10—C31	−0.3 (4)
C19—C11—C12—C20	−3.0 (6)	C45—C37—C38—C46	0.3 (7)
C15—C11—C12—C20	177.4 (4)	C41—C37—C38—C46	−179.6 (4)
C19—C11—C12—C13	178.7 (3)	C45—C37—C38—C39	−179.9 (3)
C15—C11—C12—C13	−0.8 (4)	C41—C37—C38—C39	0.2 (4)
C20—C12—C13—C14	−178.2 (3)	C46—C38—C39—C49	−1.4 (5)
C11—C12—C13—C14	0.4 (4)	C37—C38—C39—C49	178.8 (3)
C20—C12—C13—C23	0.4 (5)	C46—C38—C39—C40	179.7 (3)
C11—C12—C13—C23	179.0 (3)	C37—C38—C39—C40	−0.1 (4)
C23—C13—C14—C26	1.5 (5)	C49—C39—C40—C52	−0.1 (5)
C12—C13—C14—C26	−179.9 (3)	C38—C39—C40—C52	178.8 (3)
C23—C13—C14—C15	−178.4 (3)	C49—C39—C40—C41	−178.9 (3)
C12—C13—C14—C15	0.2 (4)	C38—C39—C40—C41	0.0 (4)
C19—C11—C15—C16	1.1 (5)	C45—C37—C41—C42	−0.5 (5)
C12—C11—C15—C16	−179.3 (3)	C38—C37—C41—C42	179.5 (3)
C19—C11—C15—C14	−178.7 (3)	C45—C37—C41—C40	179.9 (3)
C12—C11—C15—C14	0.9 (4)	C38—C37—C41—C40	−0.1 (4)
C26—C14—C15—C16	−0.3 (7)	C52—C40—C41—C42	2.1 (7)
C13—C14—C15—C16	179.6 (4)	C39—C40—C41—C42	−179.5 (3)
C26—C14—C15—C11	179.4 (4)	C52—C40—C41—C37	−178.3 (4)
C13—C14—C15—C11	−0.7 (4)	C39—C40—C41—C37	0.1 (4)
C11—C15—C16—C17	−0.5 (5)	C37—C41—C42—C43	0.7 (5)
C14—C15—C16—C17	179.2 (3)	C40—C41—C42—C43	−179.8 (3)
C15—C16—C17—C18	−0.3 (5)	C41—C42—C43—C44	−0.3 (5)
C16—C17—C18—C19	0.6 (5)	C42—C43—C44—C45	−0.4 (5)
C17—C18—C19—C11	0.1 (5)	C41—C37—C45—C44	−0.2 (5)
C15—C11—C19—C18	−0.9 (5)	C38—C37—C45—C44	179.9 (3)
C12—C11—C19—C18	179.6 (3)	C43—C44—C45—C37	0.6 (5)
C13—C12—C20—C21	0.0 (5)	C39—C38—C46—C47	1.2 (5)
C11—C12—C20—C21	−178.1 (3)	C37—C38—C46—C47	−179.0 (3)
C12—C20—C21—C22	−0.4 (5)	C38—C46—C47—C48	−0.4 (5)
C20—C21—C22—C23	0.5 (5)	C46—C47—C48—C49	−0.4 (5)
C14—C13—C23—C24	−1.1 (5)	C38—C39—C49—C50	−178.6 (3)
C12—C13—C23—C24	−179.5 (3)	C40—C39—C49—C50	0.2 (5)
C14—C13—C23—C22	178.1 (3)	C38—C39—C49—C48	0.6 (5)
C12—C13—C23—C22	−0.3 (5)	C40—C39—C49—C48	179.4 (3)
C21—C22—C23—C13	−0.2 (5)	C47—C48—C49—C39	0.3 (5)
C21—C22—C23—C24	179.0 (3)	C47—C48—C49—C50	179.4 (3)
C13—C23—C24—C25	0.7 (5)	C39—C49—C50—C51	0.4 (5)
C22—C23—C24—C25	−178.5 (3)	C48—C49—C50—C51	−178.7 (3)
C23—C24—C25—C26	−0.7 (6)	C49—C50—C51—C52	−1.0 (6)
C13—C14—C26—C25	−1.4 (5)	C39—C40—C52—C51	−0.5 (5)
C15—C14—C26—C25	178.5 (4)	C41—C40—C52—C51	177.8 (4)
C24—C25—C26—C14	1.1 (6)	C50—C51—C52—C40	1.0 (5)

### Pyromellitic acid dianhydride–fluoranthene (1/1) (II) Hydrogen-bond geometry (Å, º)

**Table:**

*D*—H···*A*	*D*—H	H···*A*	*D*···*A*	*D*—H···*A*
C8—H8···O2^i^	0.95	2.67	3.373 (5)	132
C16—H16···O8	0.95	2.59	3.444 (5)	150
C17—H17···O3^ii^	0.95	2.65	3.576 (5)	166
C18—H18···O1^ii^	0.95	2.67	3.332 (5)	127
C22—H22···O4^iii^	0.95	2.59	3.481 (5)	155
C25—H25···O11^iii^	0.95	2.55	3.347 (5)	142
C29—H29···O12^iv^	0.95	2.71	3.370 (5)	127
C42—H42···O6	0.95	2.49	3.413 (5)	165
C43—H43···O11^iii^	0.95	2.58	3.293 (5)	132
C44—H44···O10^iii^	0.95	2.52	3.428 (5)	160
C45—H45···O12^iv^	0.95	2.57	3.429 (5)	150
C46—H46···O9^v^	0.95	2.64	3.256 (5)	123
C48—H48···O9^vi^	0.95	2.55	3.473 (5)	164
C50—H50···O7^vi^	0.95	2.5	3.420 (5)	164
C52—H52···O6	0.95	2.62	3.525 (5)	159

Symmetry codes: (i) −*x*+3/2, *y*, *z*−1/2; (ii) *x*, *y*−1, *z*−1; (iii) *x*, *y*, *z*+1; (iv) −*x*+2, −*y*+1, *z*+1/2; (v) −*x*+2, −*y*+1, *z*−1/2; (vi) *x*, *y*+1, *z*−1.

### Pyromellitic acid dianhydride–9-methylanthracene (1/1) (III) Crystal data

**Table:**

C_10_H_2_O_6_·C_15_H_12_	*Z* = 2
*M**_r_* = 410.36	*F*(000) = 424
Triclinic, *P*1	*D*_x_ = 1.518 Mg m^−^^3^
Hall symbol: -P 1	Mo *K*α radiation, λ = 0.71073 Å
*a* = 7.1012 (8) Å	Cell parameters from 4805 reflections
*b* = 9.5674 (12) Å	θ = 3.5–28.2°
*c* = 13.6147 (16) Å	µ = 0.11 mm^−^^1^
α = 99.109 (4)°	*T* = 173 K
β = 99.941 (4)°	Needle, red
γ = 92.219 (4)°	0.19 × 0.06 × 0.05 mm
*V* = 897.53 (19) Å^3^	

### Pyromellitic acid dianhydride–9-methylanthracene (1/1) (III) Data collection

**Table:**

Bruker D8 Venture Photon CCD area detector diffractometer	2159 reflections with *I* > 2σ(*I*)
Graphite monochromator	*R*_int_ = 0.075
ω scans	θ_max_ = 25.5°, θ_min_ = 3.0°
Absorption correction: multi-scan (SADABS; Krause *et al.*, 2015)	*h* = −8→8
*T*_min_ = 0.9, *T*_max_ = 0.95	*k* = −11→11
20202 measured reflections	*l* = −16→16
3280 independent reflections	

### Pyromellitic acid dianhydride–9-methylanthracene (1/1) (III) Refinement

**Table:**

Refinement on *F*^2^	0 constraints
Least-squares matrix: full	Hydrogen site location: inferred from neighbouring sites
*R*[*F*^2^ > 2σ(*F*^2^)] = 0.073	H-atom parameters constrained
*wR*(*F*^2^) = 0.223	*w* = 1/[σ^2^(*F*_o_^2^) + (0.1395*P*)^2^ + 0.5023*P*] where *P* = (*F*_o_^2^ + 2*F*_c_^2^)/3
*S* = 1.02	(Δ/σ)_max_ = 0.028
3280 reflections	Δρ_max_ = 0.67 e Å^−^^3^
281 parameters	Δρ_min_ = −0.28 e Å^−^^3^
0 restraints	

### Pyromellitic acid dianhydride–9-methylanthracene (1/1) (III) Special details

**Table:**

Experimental. Absorption corrections were made using the program SADABS (Sheldrick, 1996)
Geometry. All esds (except the esd in the dihedral angle between two l.s. planes) are estimated using the full covariance matrix. The cell esds are taken into account individually in the estimation of esds in distances, angles and torsion angles; correlations between esds in cell parameters are only used when they are defined by crystal symmetry. An approximate (isotropic) treatment of cell esds is used for estimating esds involving l.s. planes.

### Pyromellitic acid dianhydride–9-methylanthracene (1/1) (III) Fractional atomic coordinates and isotropic or equivalent isotropic displacement parameters (Å^2^)

**Table:**

	*x*	*y*	*z*	*U*_iso_*/*U*_eq_	
C11	0.5511 (4)	0.7888 (3)	0.1593 (2)	0.0168 (7)	
C12	0.6280 (4)	0.6829 (3)	0.2138 (2)	0.0186 (7)	
C13	0.6688 (5)	0.5463 (3)	0.1648 (2)	0.0238 (8)	
H13	0.645706	0.525778	0.093003	0.029*	
C14	0.7394 (5)	0.4460 (3)	0.2181 (3)	0.0267 (8)	
H14	0.764739	0.356517	0.183297	0.032*	
C15	0.7760 (5)	0.4733 (4)	0.3256 (3)	0.0293 (8)	
H15	0.823867	0.401744	0.362338	0.035*	
C16	0.7426 (5)	0.6011 (4)	0.3757 (3)	0.0255 (8)	
H16	0.769232	0.618668	0.447533	0.031*	
C17	0.6678 (4)	0.7105 (3)	0.3225 (2)	0.0193 (7)	
C18	0.6349 (4)	0.8429 (3)	0.3731 (2)	0.0213 (7)	
H18	0.663705	0.86158	0.444956	0.026*	
C19	0.5610 (4)	0.9481 (3)	0.3215 (2)	0.0170 (7)	
C20	0.5291 (5)	1.0846 (3)	0.3738 (3)	0.0258 (8)	
H20	0.561001	1.104084	0.445555	0.031*	
C21	0.4535 (5)	1.1878 (4)	0.3228 (3)	0.0279 (8)	
H21	0.434305	1.277953	0.358915	0.033*	
C22	0.4041 (5)	1.1596 (3)	0.2159 (3)	0.0270 (8)	
H22	0.34882	1.230449	0.180651	0.032*	
C23	0.4348 (5)	1.0322 (3)	0.1632 (2)	0.0220 (7)	
H23	0.402008	1.016283	0.091479	0.026*	
C24	0.5155 (4)	0.9210 (3)	0.2130 (2)	0.0173 (7)	
C25	0.5090 (5)	0.7591 (4)	0.0452 (2)	0.0279 (8)	
H25A	0.61712	0.714047	0.020221	0.042*	
H25B	0.489642	0.848319	0.019345	0.042*	
H25C	0.392889	0.695435	0.022068	0.042*	
O1	0.0077 (3)	1.1492 (2)	0.41401 (18)	0.0317 (6)	
O2	−0.0687 (3)	1.1373 (2)	0.24510 (17)	0.0277 (6)	
O3	−0.1253 (3)	1.0619 (3)	0.07623 (18)	0.0333 (7)	
O4	0.2767 (4)	0.5551 (3)	0.42121 (19)	0.0380 (7)	
O5	0.2349 (3)	0.4790 (2)	0.25220 (18)	0.0313 (6)	
O6	0.1745 (4)	0.4690 (3)	0.08289 (19)	0.0383 (7)	
C1	−0.0022 (5)	1.0820 (3)	0.3321 (2)	0.0233 (8)	
C2	0.0472 (4)	0.9353 (3)	0.2990 (2)	0.0189 (7)	
C3	0.1180 (4)	0.8363 (3)	0.3567 (2)	0.0202 (7)	
H3	0.140718	0.85421	0.428537	0.024*	
C4	0.1534 (4)	0.7083 (3)	0.3013 (2)	0.0198 (7)	
C5	0.2282 (5)	0.5797 (4)	0.3378 (3)	0.0274 (8)	
C6	0.1740 (5)	0.5354 (4)	0.1647 (3)	0.0267 (8)	
C7	0.1187 (4)	0.6823 (3)	0.1960 (2)	0.0204 (7)	
C8	0.0455 (4)	0.7800 (3)	0.1375 (2)	0.0196 (7)	
H8	0.020749	0.761353	0.065698	0.023*	
C9	0.0114 (4)	0.9083 (3)	0.1936 (2)	0.0180 (7)	
C10	−0.0672 (5)	1.0373 (3)	0.1585 (2)	0.0236 (8)	

### Pyromellitic acid dianhydride–9-methylanthracene (1/1) (III) Atomic displacement parameters (Å^2^)

**Table:**

	*U*^11^	*U*^22^	*U*^33^	*U*^12^	*U*^13^	*U*^23^
C11	0.0162 (16)	0.0139 (15)	0.0196 (16)	−0.0028 (12)	0.0030 (12)	0.0017 (12)
C12	0.0155 (16)	0.0157 (16)	0.0255 (17)	−0.0010 (12)	0.0053 (13)	0.0052 (13)
C13	0.0258 (18)	0.0182 (17)	0.0272 (18)	0.0042 (14)	0.0053 (14)	0.0017 (14)
C14	0.0250 (19)	0.0147 (16)	0.041 (2)	0.0021 (14)	0.0061 (15)	0.0058 (15)
C15	0.0249 (19)	0.0211 (18)	0.046 (2)	0.0026 (14)	0.0057 (16)	0.0178 (16)
C16	0.0244 (18)	0.0283 (19)	0.0266 (18)	0.0021 (15)	0.0042 (14)	0.0137 (15)
C17	0.0153 (16)	0.0209 (17)	0.0241 (17)	0.0007 (13)	0.0078 (13)	0.0063 (13)
C18	0.0172 (17)	0.0283 (18)	0.0187 (16)	0.0007 (14)	0.0041 (13)	0.0038 (14)
C19	0.0134 (15)	0.0162 (16)	0.0215 (16)	0.0007 (12)	0.0047 (12)	0.0017 (12)
C20	0.0194 (17)	0.0196 (17)	0.0364 (19)	−0.0005 (14)	0.0107 (14)	−0.0069 (14)
C21	0.0229 (18)	0.0176 (17)	0.042 (2)	0.0039 (14)	0.0111 (15)	−0.0051 (15)
C22	0.0240 (18)	0.0195 (17)	0.038 (2)	0.0031 (14)	0.0062 (15)	0.0046 (15)
C23	0.0222 (17)	0.0198 (17)	0.0247 (17)	0.0056 (14)	0.0018 (13)	0.0072 (14)
C24	0.0149 (16)	0.0191 (16)	0.0195 (16)	−0.0012 (13)	0.0055 (12)	0.0061 (13)
C25	0.037 (2)	0.0235 (17)	0.0230 (18)	0.0082 (15)	0.0052 (15)	0.0019 (14)
O1	0.0327 (14)	0.0253 (13)	0.0343 (15)	0.0050 (11)	0.0070 (11)	−0.0055 (11)
O2	0.0291 (13)	0.0181 (12)	0.0371 (14)	0.0093 (10)	0.0068 (10)	0.0055 (10)
O3	0.0345 (15)	0.0393 (15)	0.0312 (14)	0.0162 (12)	0.0072 (11)	0.0169 (11)
O4	0.0389 (16)	0.0406 (15)	0.0427 (16)	0.0136 (12)	0.0101 (12)	0.0261 (13)
O5	0.0303 (14)	0.0202 (12)	0.0455 (15)	0.0106 (10)	0.0081 (11)	0.0083 (11)
O6	0.0379 (15)	0.0294 (14)	0.0431 (16)	0.0119 (12)	0.0058 (12)	−0.0082 (12)
C1	0.0192 (17)	0.0209 (17)	0.0307 (19)	0.0013 (14)	0.0068 (14)	0.0048 (15)
C2	0.0131 (16)	0.0224 (17)	0.0222 (17)	0.0022 (13)	0.0043 (12)	0.0050 (13)
C3	0.0164 (16)	0.0248 (17)	0.0200 (16)	0.0025 (13)	0.0027 (12)	0.0057 (13)
C4	0.0168 (16)	0.0191 (16)	0.0250 (17)	0.0020 (13)	0.0051 (13)	0.0069 (13)
C5	0.0216 (18)	0.0253 (18)	0.038 (2)	0.0055 (14)	0.0085 (15)	0.0107 (16)
C6	0.0221 (18)	0.0216 (18)	0.037 (2)	0.0080 (14)	0.0069 (15)	0.0038 (16)
C7	0.0113 (15)	0.0194 (16)	0.0285 (18)	−0.0004 (12)	0.0028 (13)	−0.0003 (13)
C8	0.0165 (16)	0.0207 (17)	0.0211 (16)	0.0010 (13)	0.0031 (12)	0.0025 (13)
C9	0.0132 (15)	0.0208 (17)	0.0220 (16)	0.0039 (13)	0.0046 (12)	0.0077 (13)
C10	0.0233 (18)	0.0220 (18)	0.0277 (18)	0.0074 (14)	0.0079 (14)	0.0065 (15)

### Pyromellitic acid dianhydride–9-methylanthracene (1/1) (III) Geometric parameters (Å, º)

**Table:**

C11—C24	1.411 (4)	C23—H23	0.95
C11—C12	1.420 (4)	C25—H25A	0.98
C11—C25	1.509 (4)	C25—H25B	0.98
C12—C13	1.434 (4)	C25—H25C	0.98
C12—C17	1.437 (4)	O1—C1	1.186 (4)
C13—C14	1.353 (4)	O2—C1	1.389 (4)
C13—H13	0.95	O2—C10	1.398 (4)
C14—C15	1.422 (5)	O3—C10	1.188 (4)
C14—H14	0.95	O4—C5	1.191 (4)
C15—C16	1.353 (5)	O5—C6	1.393 (4)
C15—H15	0.95	O5—C5	1.399 (4)
C16—C17	1.432 (4)	O6—C6	1.193 (4)
C16—H16	0.95	C1—C2	1.479 (4)
C17—C18	1.392 (4)	C2—C3	1.380 (4)
C18—C19	1.386 (4)	C2—C9	1.394 (4)
C18—H18	0.95	C3—C4	1.389 (4)
C19—C20	1.432 (4)	C3—H3	0.95
C19—C24	1.435 (4)	C4—C7	1.392 (4)
C20—C21	1.369 (5)	C4—C5	1.482 (4)
C20—H20	0.95	C6—C7	1.491 (4)
C21—C22	1.417 (5)	C7—C8	1.382 (4)
C21—H21	0.95	C8—C9	1.392 (4)
C22—C23	1.358 (4)	C8—H8	0.95
C22—H22	0.95	C9—C10	1.486 (4)
C23—C24	1.437 (4)		
			
C24—C11—C12	119.3 (3)	C11—C24—C23	122.5 (3)
C24—C11—C25	120.9 (3)	C19—C24—C23	117.6 (3)
C12—C11—C25	119.8 (3)	C11—C25—H25A	109.5
C11—C12—C13	122.6 (3)	C11—C25—H25B	109.5
C11—C12—C17	120.0 (3)	H25A—C25—H25B	109.5
C13—C12—C17	117.3 (3)	C11—C25—H25C	109.5
C14—C13—C12	121.8 (3)	H25A—C25—H25C	109.5
C14—C13—H13	119.1	H25B—C25—H25C	109.5
C12—C13—H13	119.1	C1—O2—C10	110.9 (2)
C13—C14—C15	120.6 (3)	C6—O5—C5	110.2 (2)
C13—C14—H14	119.7	O1—C1—O2	121.9 (3)
C15—C14—H14	119.7	O1—C1—C2	131.2 (3)
C16—C15—C14	120.1 (3)	O2—C1—C2	106.9 (3)
C16—C15—H15	120	C3—C2—C9	122.7 (3)
C14—C15—H15	120	C3—C2—C1	129.2 (3)
C15—C16—C17	121.3 (3)	C9—C2—C1	108.1 (3)
C15—C16—H16	119.4	C2—C3—C4	114.6 (3)
C17—C16—H16	119.4	C2—C3—H3	122.7
C18—C17—C16	121.8 (3)	C4—C3—H3	122.7
C18—C17—C12	119.3 (3)	C3—C4—C7	122.4 (3)
C16—C17—C12	118.9 (3)	C3—C4—C5	129.1 (3)
C19—C18—C17	121.7 (3)	C7—C4—C5	108.4 (3)
C19—C18—H18	119.2	O4—C5—O5	121.8 (3)
C17—C18—H18	119.2	O4—C5—C4	131.3 (3)
C18—C19—C20	121.5 (3)	O5—C5—C4	106.9 (3)
C18—C19—C24	119.8 (3)	O6—C6—O5	121.4 (3)
C20—C19—C24	118.7 (3)	O6—C6—C7	130.9 (3)
C21—C20—C19	121.4 (3)	O5—C6—C7	107.7 (3)
C21—C20—H20	119.3	C8—C7—C4	123.4 (3)
C19—C20—H20	119.3	C8—C7—C6	129.8 (3)
C20—C21—C22	119.7 (3)	C4—C7—C6	106.7 (3)
C20—C21—H21	120.1	C7—C8—C9	113.8 (3)
C22—C21—H21	120.1	C7—C8—H8	123.1
C23—C22—C21	120.7 (3)	C9—C8—H8	123.1
C23—C22—H22	119.7	C8—C9—C2	123.0 (3)
C21—C22—H22	119.7	C8—C9—C10	129.7 (3)
C22—C23—C24	121.8 (3)	C2—C9—C10	107.3 (3)
C22—C23—H23	119.1	O3—C10—O2	121.5 (3)
C24—C23—H23	119.1	O3—C10—C9	131.7 (3)
C11—C24—C19	119.9 (3)	O2—C10—C9	106.8 (3)
			
C24—C11—C12—C13	−179.8 (3)	O2—C1—C2—C3	−179.6 (3)
C25—C11—C12—C13	0.1 (5)	O1—C1—C2—C9	−179.0 (3)
C24—C11—C12—C17	0.3 (4)	O2—C1—C2—C9	0.8 (3)
C25—C11—C12—C17	−179.8 (3)	C9—C2—C3—C4	0.9 (5)
C11—C12—C13—C14	−179.0 (3)	C1—C2—C3—C4	−178.6 (3)
C17—C12—C13—C14	0.9 (5)	C2—C3—C4—C7	−0.4 (5)
C12—C13—C14—C15	−0.1 (5)	C2—C3—C4—C5	−179.8 (3)
C13—C14—C15—C16	−0.9 (5)	C6—O5—C5—O4	−178.9 (3)
C14—C15—C16—C17	0.9 (5)	C6—O5—C5—C4	1.3 (4)
C15—C16—C17—C18	−179.3 (3)	C3—C4—C5—O4	−0.7 (6)
C15—C16—C17—C12	0.0 (5)	C7—C4—C5—O4	179.8 (4)
C11—C12—C17—C18	−1.7 (4)	C3—C4—C5—O5	179.1 (3)
C13—C12—C17—C18	178.4 (3)	C7—C4—C5—O5	−0.4 (4)
C11—C12—C17—C16	179.0 (3)	C5—O5—C6—O6	177.4 (3)
C13—C12—C17—C16	−0.9 (4)	C5—O5—C6—C7	−1.6 (4)
C16—C17—C18—C19	−179.5 (3)	C3—C4—C7—C8	−0.4 (5)
C12—C17—C18—C19	1.3 (5)	C5—C4—C7—C8	179.1 (3)
C17—C18—C19—C20	−179.4 (3)	C3—C4—C7—C6	179.9 (3)
C17—C18—C19—C24	0.5 (5)	C5—C4—C7—C6	−0.5 (3)
C18—C19—C20—C21	−179.0 (3)	O6—C6—C7—C8	2.8 (6)
C24—C19—C20—C21	1.1 (5)	O5—C6—C7—C8	−178.3 (3)
C19—C20—C21—C22	0.5 (5)	O6—C6—C7—C4	−177.6 (4)
C20—C21—C22—C23	−1.5 (5)	O5—C6—C7—C4	1.3 (4)
C21—C22—C23—C24	0.8 (5)	C4—C7—C8—C9	0.7 (5)
C12—C11—C24—C19	1.4 (4)	C6—C7—C8—C9	−179.7 (3)
C25—C11—C24—C19	−178.5 (3)	C7—C8—C9—C2	−0.2 (4)
C12—C11—C24—C23	−178.9 (3)	C7—C8—C9—C10	−179.5 (3)
C25—C11—C24—C23	1.3 (5)	C3—C2—C9—C8	−0.6 (5)
C18—C19—C24—C11	−1.8 (4)	C1—C2—C9—C8	179.0 (3)
C20—C19—C24—C11	178.0 (3)	C3—C2—C9—C10	178.8 (3)
C18—C19—C24—C23	178.4 (3)	C1—C2—C9—C10	−1.7 (3)
C20—C19—C24—C23	−1.7 (4)	C1—O2—C10—O3	177.3 (3)
C22—C23—C24—C11	−179.0 (3)	C1—O2—C10—C9	−1.4 (3)
C22—C23—C24—C19	0.8 (5)	C8—C9—C10—O3	2.7 (6)
C10—O2—C1—O1	−179.7 (3)	C2—C9—C10—O3	−176.6 (4)
C10—O2—C1—C2	0.4 (3)	C8—C9—C10—O2	−178.8 (3)
O1—C1—C2—C3	0.5 (6)	C2—C9—C10—O2	1.9 (3)

### Pyromellitic acid dianhydride–9-methylanthracene (1/1) (III) Hydrogen-bond geometry (Å, º)

**Table:**

*D*—H···*A*	*D*—H	H···*A*	*D*···*A*	*D*—H···*A*
C3—H3···O1^i^	0.95	2.55	3.376 (4)	145
C14—H14···O2^ii^	0.95	2.63	3.347 (4)	133
C16—H16···O4^iii^	0.95	2.68	3.365 (4)	130
C22—H22···O5^iv^	0.95	2.64	3.323 (4)	130

Symmetry codes: (i) −*x*, −*y*+2, −*z*+1; (ii) *x*+1, *y*−1, *z*; (iii) −*x*+1, −*y*+1, −*z*+1; (iv) *x*, *y*+1, *z*.

### Pyromellitic acid dianhydride–ethyl anthracene-9-carboxylate (1/2) (IV) Crystal data

**Table:**

C_17_H_12_O_3_·0.5C_10_H_2_O_6_	*F*(000) = 772
*M**_r_* = 373.32	*D*_x_ = 1.484 Mg m^−^^3^
Monoclinic, *P*2_1_/*c*	Mo *K*α radiation, λ = 0.71073 Å
Hall symbol: -P 2ybc	Cell parameters from 2311 reflections
*a* = 9.1949 (7) Å	θ = 2.3–26.6°
*b* = 17.9751 (14) Å	µ = 0.11 mm^−^^1^
*c* = 10.9716 (10) Å	*T* = 173 K
β = 112.829 (2)°	Plate, red
*V* = 1671.3 (2) Å^3^	0.55 × 0.1 × 0.06 mm
*Z* = 4	

### Pyromellitic acid dianhydride–ethyl anthracene-9-carboxylate (1/2) (IV) Data collection

**Table:**

Bruker D8 Venture Photon CCD area detector diffractometer	2731 reflections with *I* > 2σ(*I*)
Graphite monochromator	*R*_int_ = 0.046
ω scans	θ_max_ = 28.0°, θ_min_ = 2.3°
Absorption correction: multi-scan (SADABS; Krause *et al.*, 2015)	*h* = −12→12
*T*_min_ = 0.9, *T*_max_ = 0.95	*k* = −23→21
13071 measured reflections	*l* = −14→14
4035 independent reflections	

### Pyromellitic acid dianhydride–ethyl anthracene-9-carboxylate (1/2) (IV) Refinement

**Table:**

Refinement on *F*^2^	0 constraints
Least-squares matrix: full	Hydrogen site location: inferred from neighbouring sites
*R*[*F*^2^ > 2σ(*F*^2^)] = 0.043	H-atom parameters constrained
*wR*(*F*^2^) = 0.112	*w* = 1/[σ^2^(*F*_o_^2^) + (0.0487*P*)^2^ + 0.0752*P*] where *P* = (*F*_o_^2^ + 2*F*_c_^2^)/3
*S* = 1.05	(Δ/σ)_max_ < 0.001
4035 reflections	Δρ_max_ = 0.30 e Å^−^^3^
254 parameters	Δρ_min_ = −0.26 e Å^−^^3^
0 restraints	

### Pyromellitic acid dianhydride–ethyl anthracene-9-carboxylate (1/2) (IV) Special details

**Table:**

Experimental. Absorption corrections were made using the program SADABS (Sheldrick, 1996)
Geometry. All esds (except the esd in the dihedral angle between two l.s. planes) are estimated using the full covariance matrix. The cell esds are taken into account individually in the estimation of esds in distances, angles and torsion angles; correlations between esds in cell parameters are only used when they are defined by crystal symmetry. An approximate (isotropic) treatment of cell esds is used for estimating esds involving l.s. planes.

### Pyromellitic acid dianhydride–ethyl anthracene-9-carboxylate (1/2) (IV) Fractional atomic coordinates and isotropic or equivalent isotropic displacement parameters (Å^2^)

**Table:**

	*x*	*y*	*z*	*U*_iso_*/*U*_eq_	
C1	0.63812 (17)	0.54682 (8)	0.55902 (15)	0.0264 (3)	
H1	0.728631	0.57749	0.597638	0.032*	
C2	0.49342 (18)	0.57369 (8)	0.47345 (15)	0.0251 (3)	
C3	0.44815 (19)	0.65055 (9)	0.42556 (16)	0.0306 (4)	
C4	0.22896 (19)	0.57678 (9)	0.33296 (15)	0.0302 (4)	
C5	0.36097 (17)	0.52887 (8)	0.41673 (14)	0.0243 (3)	
O1	0.28769 (13)	0.64923 (6)	0.34113 (11)	0.0342 (3)	
O2	0.52155 (15)	0.70646 (6)	0.44651 (13)	0.0415 (3)	
O3	0.09445 (13)	0.56283 (7)	0.26877 (12)	0.0417 (3)	
C11	0.55637 (17)	0.42627 (8)	0.88576 (14)	0.0238 (3)	
C12	0.60955 (18)	0.35455 (9)	0.94068 (15)	0.0296 (4)	
H12	0.539628	0.313325	0.913622	0.035*	
C13	0.75932 (19)	0.34440 (9)	1.03144 (16)	0.0342 (4)	
H13	0.791473	0.29626	1.067614	0.041*	
C14	0.86796 (19)	0.40408 (10)	1.07291 (16)	0.0357 (4)	
H14	0.971959	0.395898	1.136131	0.043*	
C15	0.82309 (18)	0.47274 (10)	1.02220 (15)	0.0322 (4)	
H15	0.896493	0.512595	1.050527	0.039*	
C16	0.66755 (17)	0.48668 (8)	0.92684 (14)	0.0251 (3)	
C17	0.62178 (18)	0.55718 (8)	0.87435 (15)	0.0278 (3)	
H17	0.696566	0.596523	0.901315	0.033*	
C18	0.46992 (17)	0.57207 (8)	0.78354 (14)	0.0248 (3)	
C19	0.4253 (2)	0.64542 (9)	0.73299 (16)	0.0319 (4)	
H19	0.500385	0.684592	0.760919	0.038*	
C20	0.2764 (2)	0.65969 (9)	0.64539 (16)	0.0339 (4)	
H20	0.248288	0.708695	0.612195	0.041*	
C21	0.1630 (2)	0.60214 (9)	0.60322 (16)	0.0323 (4)	
H21	0.059209	0.612936	0.542045	0.039*	
C22	0.20050 (17)	0.53167 (9)	0.64893 (15)	0.0271 (3)	
H22	0.122112	0.493954	0.619804	0.032*	
C23	0.35631 (16)	0.51312 (8)	0.74042 (14)	0.0223 (3)	
C24	0.40167 (16)	0.44101 (8)	0.79185 (14)	0.0230 (3)	
C25	0.28407 (17)	0.38021 (8)	0.74148 (15)	0.0250 (3)	
C26	0.18246 (19)	0.27453 (9)	0.81897 (17)	0.0346 (4)	
C27	0.0880 (2)	0.26785 (10)	0.9011 (2)	0.0451 (5)	
H27A	0.023888	0.222539	0.8766	0.068*	
H27B	0.018793	0.311258	0.886488	0.068*	
H27C	0.158797	0.265338	0.99463	0.068*	
O4	0.21058 (13)	0.36619 (6)	0.62747 (11)	0.0335 (3)	
O5	0.26000 (12)	0.34368 (6)	0.84255 (11)	0.0313 (3)	
O6	0.20212 (17)	0.22943 (7)	0.74753 (14)	0.0532 (4)	

### Pyromellitic acid dianhydride–ethyl anthracene-9-carboxylate (1/2) (IV) Atomic displacement parameters (Å^2^)

**Table:**

	*U*^11^	*U*^22^	*U*^33^	*U*^12^	*U*^13^	*U*^23^
C1	0.0272 (8)	0.0265 (8)	0.0271 (8)	−0.0014 (6)	0.0121 (6)	−0.0029 (6)
C2	0.0308 (8)	0.0239 (8)	0.0242 (7)	0.0019 (6)	0.0145 (6)	−0.0001 (6)
C3	0.0382 (9)	0.0265 (9)	0.0318 (8)	0.0056 (7)	0.0185 (7)	0.0034 (7)
C4	0.0342 (9)	0.0329 (9)	0.0239 (8)	0.0073 (7)	0.0116 (7)	0.0017 (7)
C5	0.0256 (8)	0.0275 (8)	0.0213 (7)	0.0036 (6)	0.0108 (6)	0.0006 (6)
O1	0.0379 (7)	0.0295 (6)	0.0346 (6)	0.0106 (5)	0.0136 (5)	0.0061 (5)
O2	0.0503 (7)	0.0221 (6)	0.0567 (8)	0.0005 (5)	0.0259 (6)	0.0035 (5)
O3	0.0299 (6)	0.0495 (8)	0.0363 (7)	0.0073 (6)	0.0027 (5)	0.0015 (6)
C11	0.0262 (8)	0.0277 (8)	0.0206 (7)	0.0000 (6)	0.0125 (6)	−0.0014 (6)
C12	0.0322 (8)	0.0274 (9)	0.0297 (8)	0.0015 (7)	0.0127 (7)	0.0026 (6)
C13	0.0383 (9)	0.0332 (9)	0.0318 (9)	0.0100 (8)	0.0144 (7)	0.0045 (7)
C14	0.0275 (8)	0.0492 (11)	0.0265 (8)	0.0072 (8)	0.0061 (7)	−0.0015 (7)
C15	0.0258 (8)	0.0411 (10)	0.0284 (8)	−0.0051 (7)	0.0091 (7)	−0.0076 (7)
C16	0.0264 (8)	0.0289 (8)	0.0223 (7)	−0.0021 (6)	0.0119 (6)	−0.0040 (6)
C17	0.0288 (8)	0.0273 (9)	0.0290 (8)	−0.0079 (7)	0.0132 (7)	−0.0058 (6)
C18	0.0313 (8)	0.0230 (8)	0.0242 (7)	−0.0002 (6)	0.0156 (6)	−0.0032 (6)
C19	0.0414 (9)	0.0232 (8)	0.0372 (9)	−0.0030 (7)	0.0218 (8)	−0.0019 (7)
C20	0.0465 (10)	0.0250 (8)	0.0367 (9)	0.0085 (8)	0.0232 (8)	0.0056 (7)
C21	0.0346 (9)	0.0344 (9)	0.0289 (8)	0.0098 (7)	0.0134 (7)	0.0014 (7)
C22	0.0266 (8)	0.0275 (8)	0.0276 (8)	0.0017 (6)	0.0112 (6)	−0.0021 (6)
C23	0.0256 (7)	0.0240 (8)	0.0204 (7)	0.0013 (6)	0.0123 (6)	−0.0012 (6)
C24	0.0242 (7)	0.0243 (8)	0.0228 (7)	−0.0019 (6)	0.0118 (6)	−0.0022 (6)
C25	0.0241 (7)	0.0227 (8)	0.0289 (8)	0.0002 (6)	0.0110 (6)	−0.0003 (6)
C26	0.0334 (9)	0.0278 (9)	0.0362 (9)	−0.0067 (7)	0.0065 (7)	0.0033 (7)
C27	0.0367 (10)	0.0422 (10)	0.0581 (12)	−0.0048 (8)	0.0203 (9)	0.0160 (9)
O4	0.0363 (6)	0.0304 (6)	0.0289 (6)	−0.0059 (5)	0.0072 (5)	−0.0030 (5)
O5	0.0362 (6)	0.0282 (6)	0.0317 (6)	−0.0117 (5)	0.0158 (5)	−0.0025 (5)
O6	0.0713 (10)	0.0330 (7)	0.0551 (9)	−0.0128 (7)	0.0244 (8)	−0.0085 (6)

### Pyromellitic acid dianhydride–ethyl anthracene-9-carboxylate (1/2) (IV) Geometric parameters (Å, º)

**Table:**

C1—C2	1.384 (2)	C17—C18	1.391 (2)
C1—C5^i^	1.386 (2)	C17—H17	0.95
C1—H1	0.95	C18—C19	1.428 (2)
C2—C5	1.390 (2)	C18—C23	1.433 (2)
C2—C3	1.479 (2)	C19—C20	1.357 (2)
C3—O2	1.1824 (19)	C19—H19	0.95
C3—O1	1.4054 (19)	C20—C21	1.413 (2)
C4—O3	1.1885 (19)	C20—H20	0.95
C4—O1	1.399 (2)	C21—C22	1.357 (2)
C4—C5	1.481 (2)	C21—H21	0.95
C11—C24	1.420 (2)	C22—C23	1.434 (2)
C11—C12	1.427 (2)	C22—H22	0.95
C11—C16	1.439 (2)	C23—C24	1.411 (2)
C12—C13	1.363 (2)	C24—C25	1.485 (2)
C12—H12	0.95	C25—O4	1.1954 (18)
C13—C14	1.415 (2)	C25—O5	1.3786 (18)
C13—H13	0.95	C26—O6	1.189 (2)
C14—C15	1.351 (2)	C26—O5	1.4061 (19)
C14—H14	0.95	C26—C27	1.479 (2)
C15—C16	1.429 (2)	C27—H27A	0.98
C15—H15	0.95	C27—H27B	0.98
C16—C17	1.388 (2)	C27—H27C	0.98
			
C2—C1—C5^i^	113.92 (14)	C18—C17—H17	119
C2—C1—H1	123	C17—C18—C19	120.89 (14)
C5^i^—C1—H1	123	C17—C18—C23	119.65 (13)
C1—C2—C5	123.11 (14)	C19—C18—C23	119.45 (14)
C1—C2—C3	129.11 (15)	C20—C19—C18	120.57 (15)
C5—C2—C3	107.78 (13)	C20—C19—H19	119.7
O2—C3—O1	121.11 (14)	C18—C19—H19	119.7
O2—C3—C2	131.61 (16)	C19—C20—C21	120.45 (15)
O1—C3—C2	107.28 (13)	C19—C20—H20	119.8
O3—C4—O1	121.42 (14)	C21—C20—H20	119.8
O3—C4—C5	131.21 (16)	C22—C21—C20	120.88 (15)
O1—C4—C5	107.36 (13)	C22—C21—H21	119.6
C1^i^—C5—C2	122.97 (14)	C20—C21—H21	119.6
C1^i^—C5—C4	129.23 (14)	C21—C22—C23	121.16 (15)
C2—C5—C4	107.80 (14)	C21—C22—H22	119.4
C4—O1—C3	109.78 (11)	C23—C22—H22	119.4
C24—C11—C12	123.88 (14)	C24—C23—C18	118.87 (13)
C24—C11—C16	118.45 (13)	C24—C23—C22	123.63 (13)
C12—C11—C16	117.66 (13)	C18—C23—C22	117.48 (13)
C13—C12—C11	120.88 (15)	C23—C24—C11	121.26 (13)
C13—C12—H12	119.6	C23—C24—C25	117.89 (13)
C11—C12—H12	119.6	C11—C24—C25	120.82 (13)
C12—C13—C14	121.36 (15)	O4—C25—O5	122.63 (14)
C12—C13—H13	119.3	O4—C25—C24	125.38 (14)
C14—C13—H13	119.3	O5—C25—C24	111.86 (12)
C15—C14—C13	119.70 (15)	O6—C26—O5	121.88 (16)
C15—C14—H14	120.1	O6—C26—C27	128.43 (16)
C13—C14—H14	120.1	O5—C26—C27	109.59 (15)
C14—C15—C16	121.38 (15)	C26—C27—H27A	109.5
C14—C15—H15	119.3	C26—C27—H27B	109.5
C16—C15—H15	119.3	H27A—C27—H27B	109.5
C17—C16—C15	121.34 (14)	C26—C27—H27C	109.5
C17—C16—C11	119.67 (13)	H27A—C27—H27C	109.5
C15—C16—C11	118.99 (14)	H27B—C27—H27C	109.5
C16—C17—C18	122.10 (14)	C25—O5—C26	120.09 (12)
C16—C17—H17	119		
			
C5^i^—C1—C2—C5	−0.1 (2)	C11—C16—C17—C18	0.8 (2)
C5^i^—C1—C2—C3	179.81 (14)	C16—C17—C18—C19	178.85 (14)
C1—C2—C3—O2	0.7 (3)	C16—C17—C18—C23	−0.9 (2)
C5—C2—C3—O2	−179.41 (17)	C17—C18—C19—C20	−179.35 (15)
C1—C2—C3—O1	−179.62 (14)	C23—C18—C19—C20	0.4 (2)
C5—C2—C3—O1	0.30 (16)	C18—C19—C20—C21	0.3 (2)
C1—C2—C5—C1^i^	0.1 (3)	C19—C20—C21—C22	−0.2 (2)
C3—C2—C5—C1^i^	−179.82 (13)	C20—C21—C22—C23	−0.5 (2)
C1—C2—C5—C4	179.33 (14)	C17—C18—C23—C24	0.2 (2)
C3—C2—C5—C4	−0.59 (16)	C19—C18—C23—C24	−179.56 (13)
O3—C4—C5—C1^i^	1.0 (3)	C17—C18—C23—C22	178.68 (13)
O1—C4—C5—C1^i^	179.84 (15)	C19—C18—C23—C22	−1.0 (2)
O3—C4—C5—C2	−178.20 (17)	C21—C22—C23—C24	179.59 (14)
O1—C4—C5—C2	0.68 (16)	C21—C22—C23—C18	1.1 (2)
O3—C4—O1—C3	178.52 (15)	C18—C23—C24—C11	0.6 (2)
C5—C4—O1—C3	−0.50 (15)	C22—C23—C24—C11	−177.83 (13)
O2—C3—O1—C4	179.89 (15)	C18—C23—C24—C25	−177.47 (12)
C2—C3—O1—C4	0.14 (16)	C22—C23—C24—C25	4.1 (2)
C24—C11—C12—C13	−179.39 (14)	C12—C11—C24—C23	−179.58 (13)
C16—C11—C12—C13	1.7 (2)	C16—C11—C24—C23	−0.7 (2)
C11—C12—C13—C14	−1.0 (2)	C12—C11—C24—C25	−1.6 (2)
C12—C13—C14—C15	0.2 (2)	C16—C11—C24—C25	177.35 (13)
C13—C14—C15—C16	−0.1 (2)	C23—C24—C25—O4	50.9 (2)
C14—C15—C16—C17	−179.71 (15)	C11—C24—C25—O4	−127.22 (17)
C14—C15—C16—C11	0.8 (2)	C23—C24—C25—O5	−125.00 (14)
C24—C11—C16—C17	0.0 (2)	C11—C24—C25—O5	56.92 (17)
C12—C11—C16—C17	178.95 (13)	O4—C25—O5—C26	18.8 (2)
C24—C11—C16—C15	179.41 (13)	C24—C25—O5—C26	−165.19 (13)
C12—C11—C16—C15	−1.6 (2)	O6—C26—O5—C25	37.5 (2)
C15—C16—C17—C18	−178.63 (14)	C27—C26—O5—C25	−146.03 (14)

Symmetry code: (i) −*x*+1, −*y*+1, −*z*+1.

### Pyromellitic acid dianhydride–ethyl anthracene-9-carboxylate (1/2) (IV) Hydrogen-bond geometry (Å, º)

**Table:**

*D*—H···*A*	*D*—H	H···*A*	*D*···*A*	*D*—H···*A*
C12—H12···O2^ii^	0.95	2.65	3.351 (2)	131
C15—H15···O3^iii^	0.95	2.55	3.306 (2)	137
C21—H21···O4^iv^	0.95	2.48	3.433 (2)	176

Symmetry codes: (ii) −*x*+1, *y*−1/2, −*z*+3/2; (iii) *x*+1, *y*, *z*+1; (iv) −*x*, −*y*+1, −*z*+1.
