# Supplementary material for: Binary charge-transfer complexes using pyromellitic acid dianhydride featuring C—H⋯O hydrogen bonds
Source: Acta Crystallogr E Crystallogr Commun. 2018 Nov 9;74(Pt 12):1772–7. doi: 10.1107/S2056989018015645 (PMC6281116; doi:10.1107/S2056989018015645)
Supplement: Supplementary file 6 [file e-74-01772-sup6.pdf]

PYMDAN

ALL: 100 %

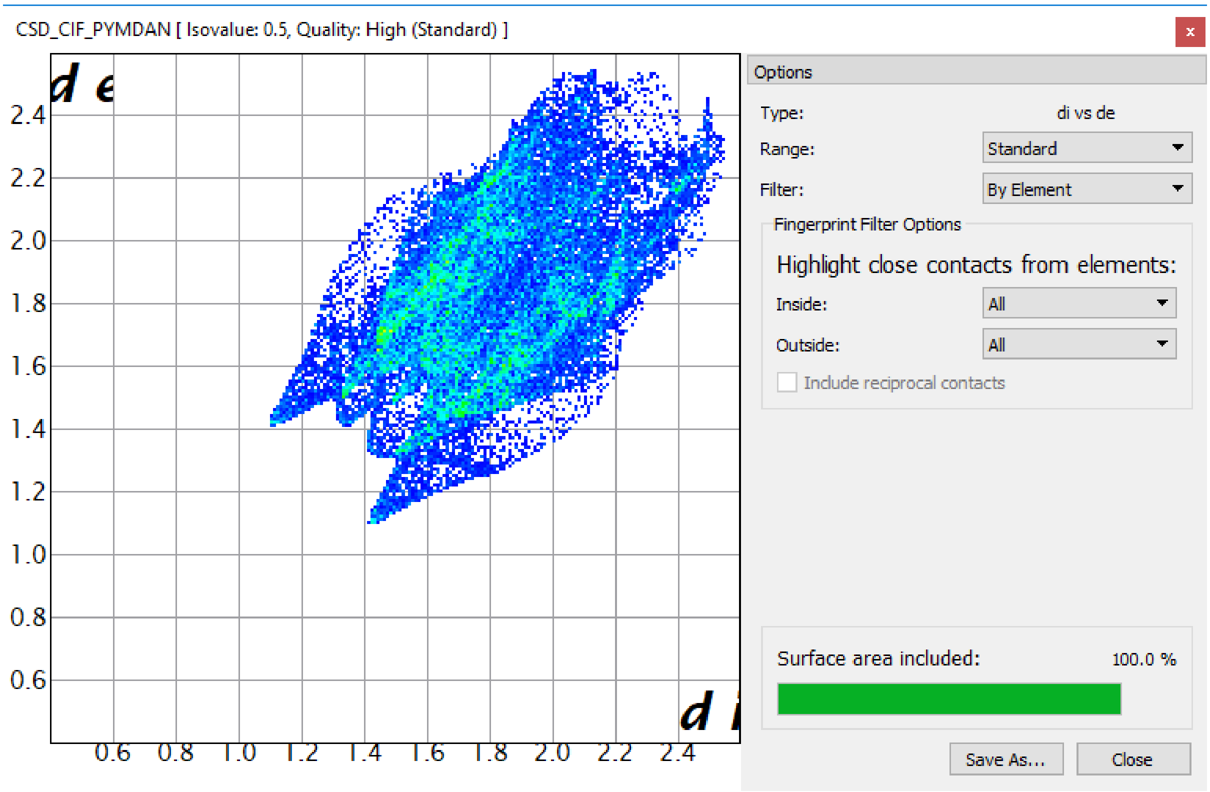

C-C: 0.2 %

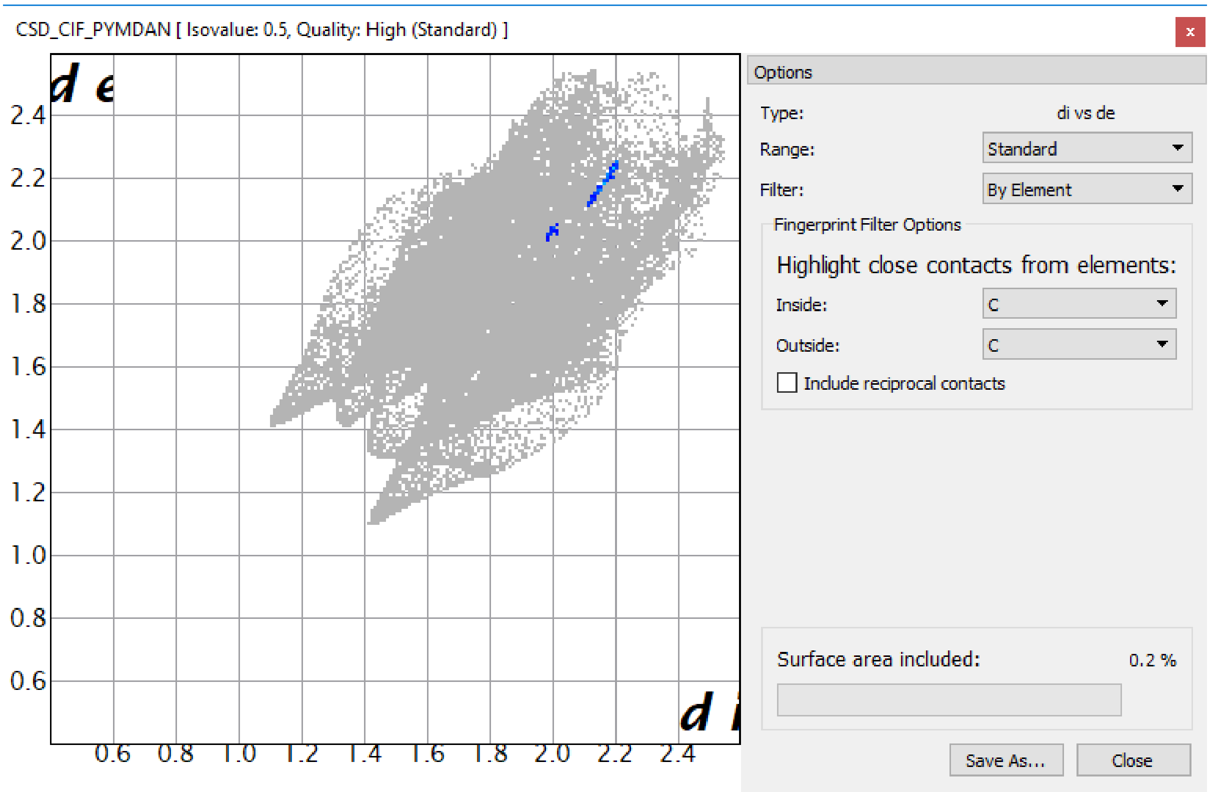
CSD_CIF_PYMDAN [Isovalue: 0.5, Quality: High (Standard)]
d e
2.4
2.2
2.0
1.8
1.6
1.4
1.2
1.0
0.8
0.6
d i
0.6 0.8 1.0 1.2 1.4 1.6 1.8 2.0 2.2 2.4
Options
Type: di vs de
Range: Standard
Filter: By Element
Fingerprint Filter Options
Highlight close contacts from elements:
Inside: C
Outside: C
Include reciprocal contacts
Surface area included: 0.2 %
Save As...
Close

O-O: 29.9 %

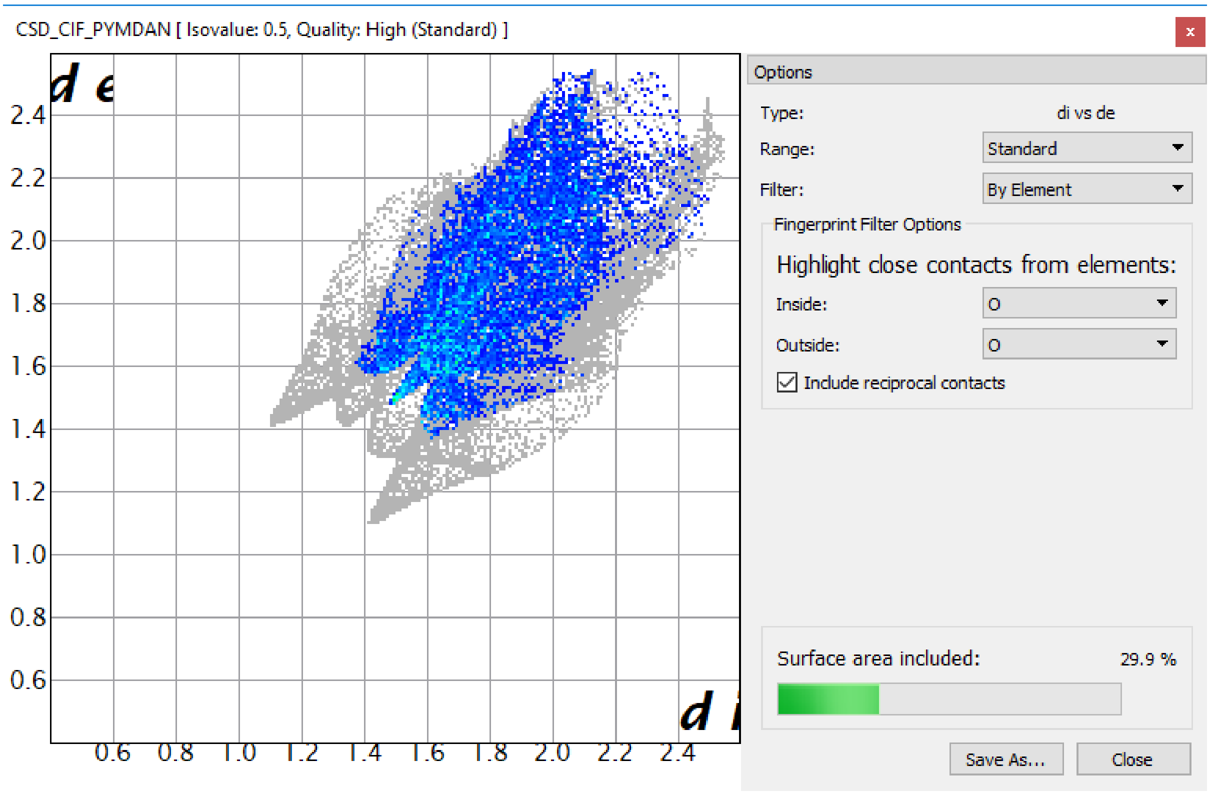
CSD_CIF_PYMDAN [Isovalue: 0.5, Quality: High (Standard)]
d e
2.4
2.2
2.0
1.8
1.6
1.4
1.2
1.0
0.8
0.6
d i
0.6 0.8 1.0 1.2 1.4 1.6 1.8 2.0 2.2 2.4
Options
Type: di vs de
Range: Standard
Filter: By Element
Fingerprint Filter Options
Highlight close contacts from elements:
Inside: O
Outside: O
Include reciprocal contacts
Surface area included: 29.9 %
Save As...
Close

H-H: 8.0 %

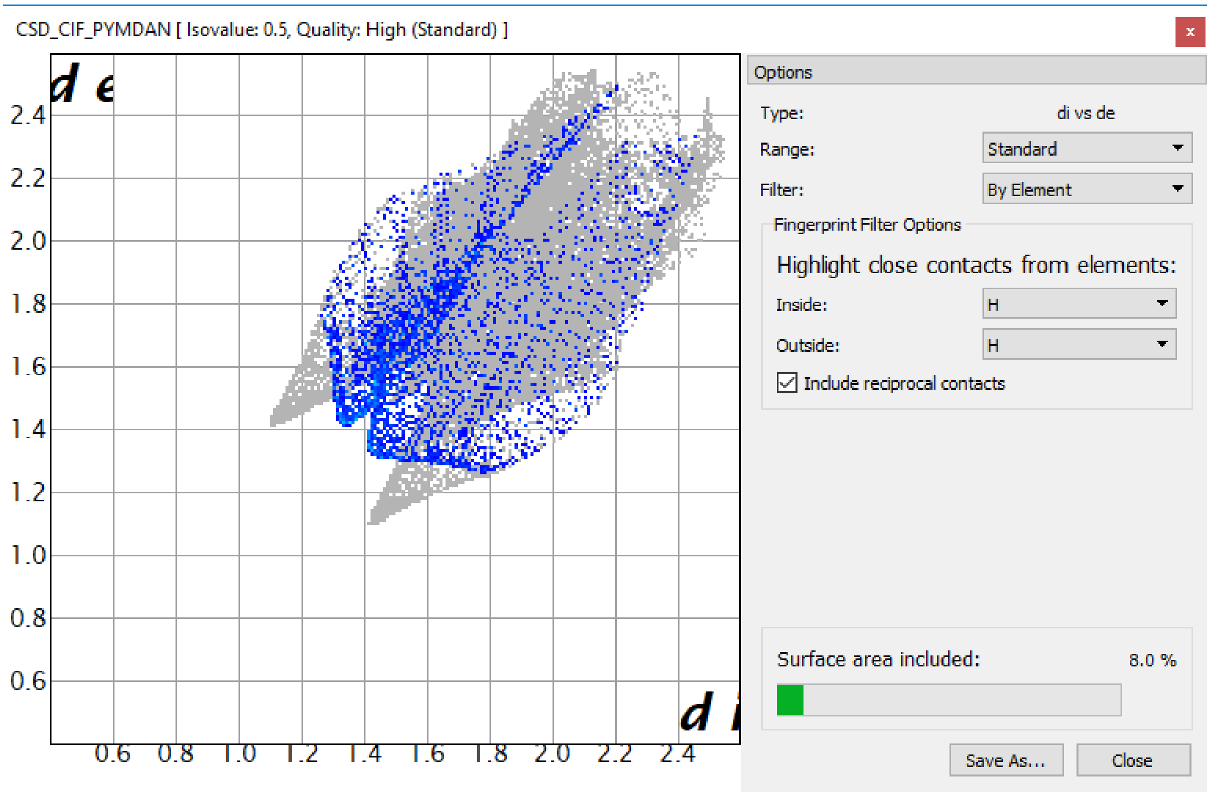
CSD_CIF_PYMDAN [Isovalue: 0.5, Quality: High (Standard)]
d e
2.4
2.2
2.0
1.8
1.6
1.4
1.2
1.0
0.8
0.6
d i
0.6 0.8 1.0 1.2 1.4 1.6 1.8 2.0 2.2 2.4
Options
Type: di vs de
Range: Standard
Filter: By Element
Fingerprint Filter Options
Highlight close contacts from elements:
Inside: H
Outside: H
Include reciprocal contacts
Surface area included: 8.0 %
Save As...
Close

C-H: 1.0 %

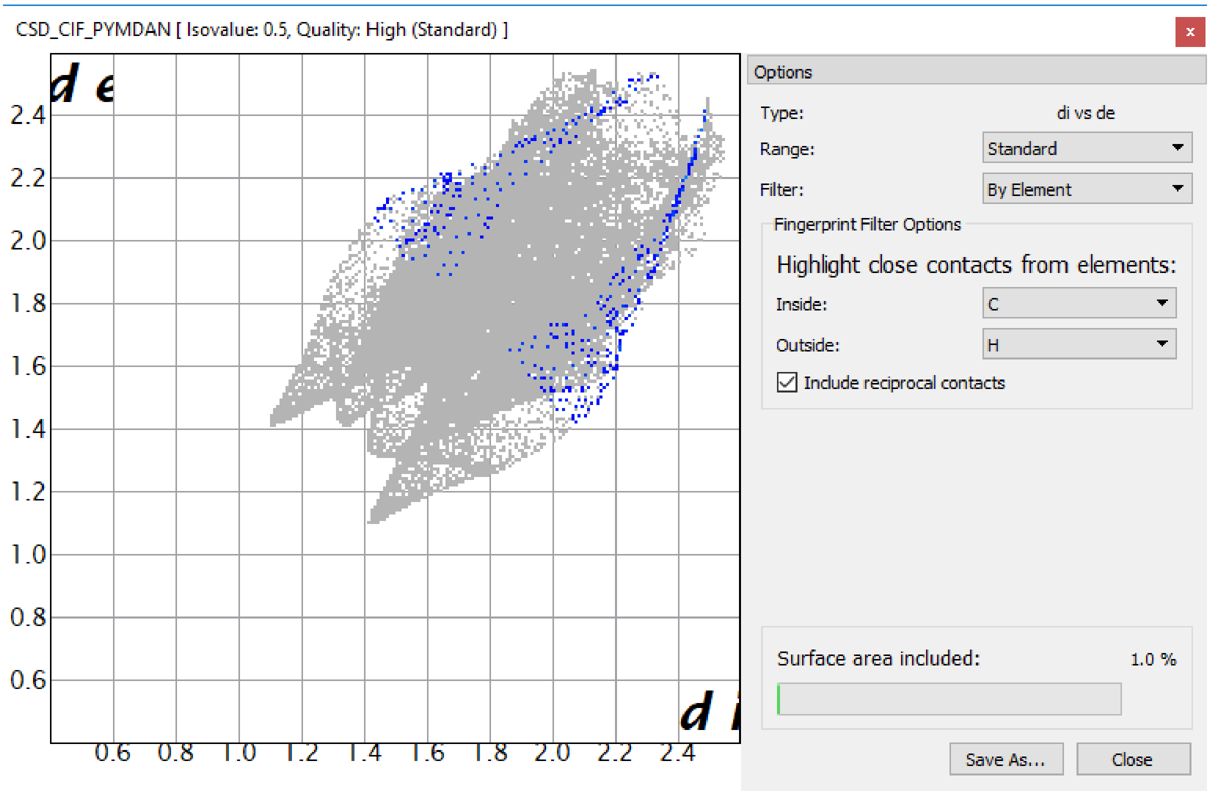
CSD_CIF_PYMDAN [Isovalue: 0.5, Quality: High (Standard)]
d e
2.4
2.2
2.0
1.8
1.6
1.4
1.2
1.0
0.8
0.6
d i
0.6 0.8 1.0 1.2 1.4 1.6 1.8 2.0 2.2 2.4
Options
Type: di vs de
Range: Standard
Filter: By Element
Fingerprint Filter Options
Highlight close contacts from elements:
Inside: C
Outside: H
Include reciprocal contacts
Surface area included: 1.0 %
Save As...
Close

C-O: 43.0 %

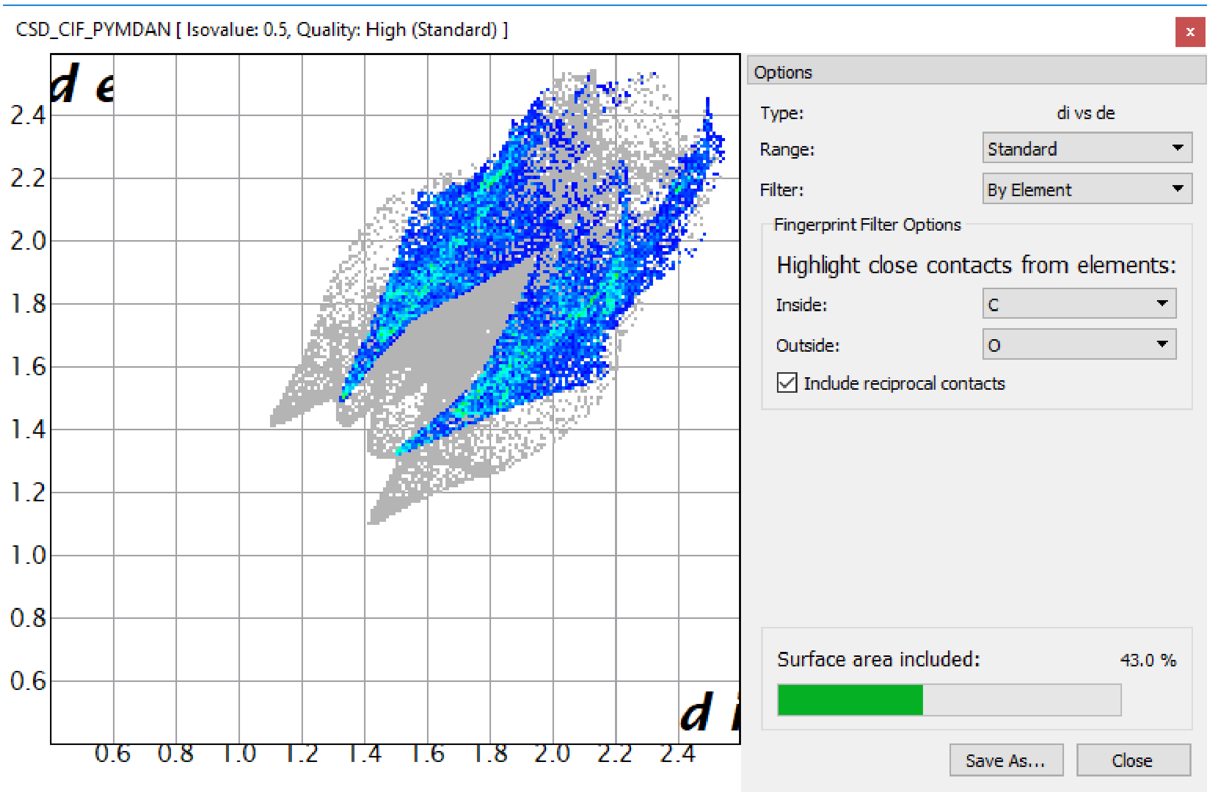
CSD_CIF_PYMDAN [Isovalue: 0.5, Quality: High (Standard)]
d e
2.4
2.2
2.0
1.8
1.6
1.4
1.2
1.0
0.8
0.6
d i
0.6 0.8 1.0 1.2 1.4 1.6 1.8 2.0 2.2 2.4
Options
Type: di vs de
Range: Standard
Filter: By Element
Fingerprint Filter Options
Highlight close contacts from elements:
Inside: C
Outside: O
Include reciprocal contacts
Surface area included: 43.0 %
Save As...
Close

O-H: 17.9 %

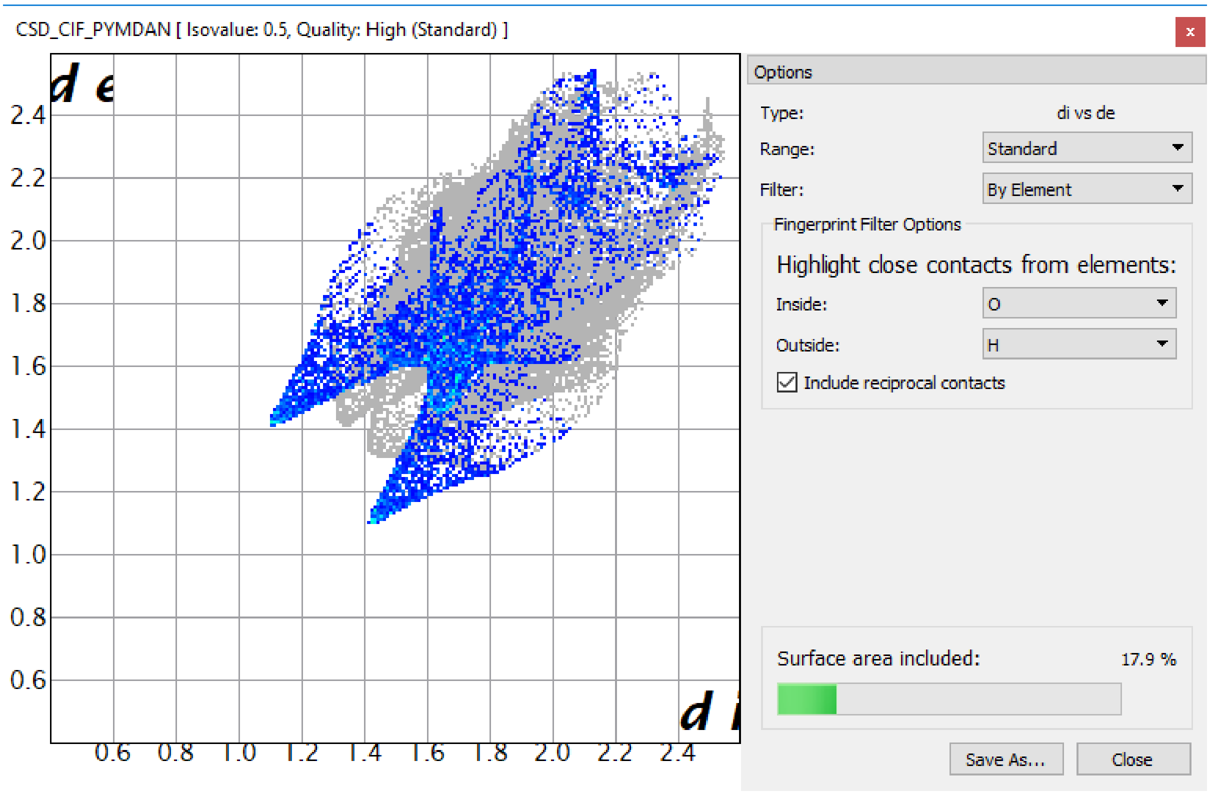
CSD_CIF_PYMDAN [Isovalue: 0.5, Quality: High (Standard)]
d e
2.4
2.2
2.0
1.8
1.6
1.4
1.2
1.0
0.8
0.6
d i
0.6 0.8 1.0 1.2 1.4 1.6 1.8 2.0 2.2 2.4
Options
Type: di vs de
Range: Standard
Filter: By Element
Fingerprint Filter Options
Highlight close contacts from elements:
Inside: O
Outside: H
Include reciprocal contacts
Surface area included: 17.9 %
Save As...
Close

(I)

ALL: 100 %

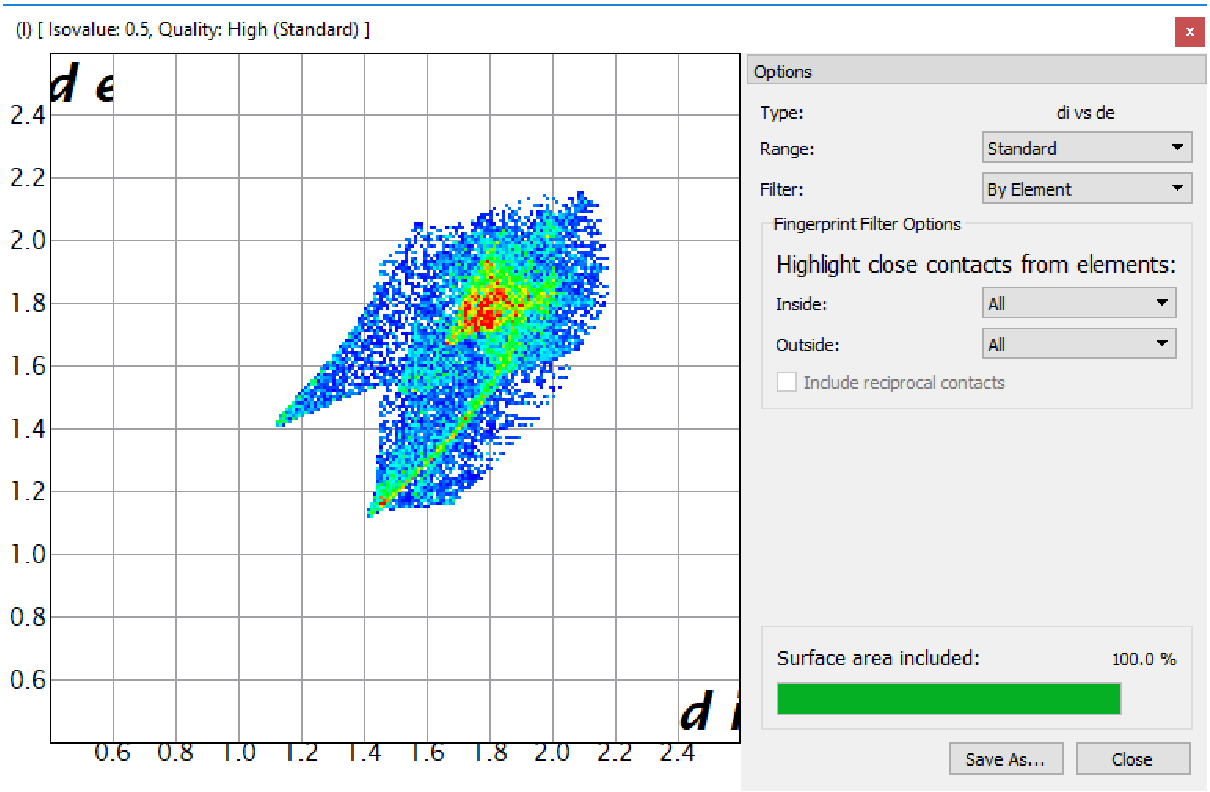
(I) [Isovalue: 0.5, Quality: High (Standard)]
d e
2.4
2.2
2.0
1.8
1.6
1.4
1.2
1.0
0.8
0.6
d i
0.6 0.8 1.0 1.2 1.4 1.6 1.8 2.0 2.2 2.4
Options
Type: di vs de
Range: Standard
Filter: By Element
Fingerprint Filter Options
Highlight close contacts from elements:
Inside: All
Outside: All
Include reciprocal contacts
Surface area included: 100.0 %
Save As...
Close

C-C: 19.8 %

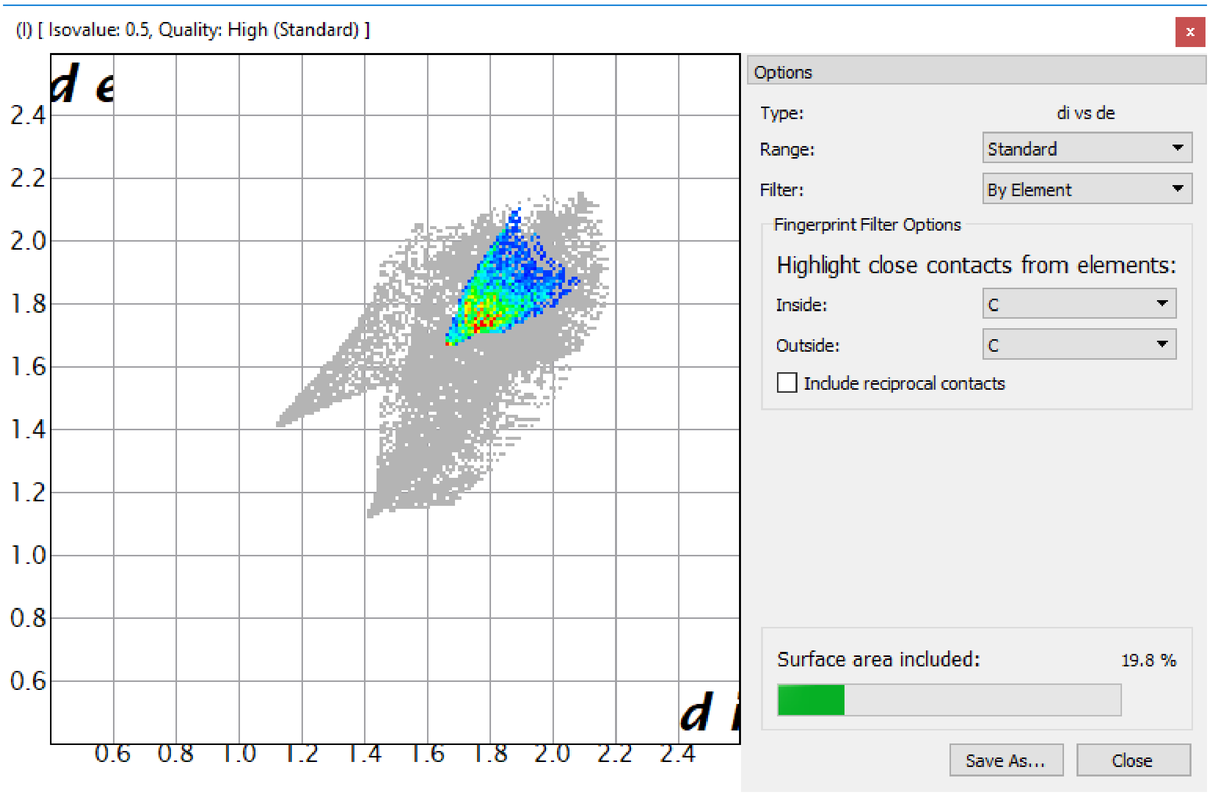
(I) [Isovalue: 0.5, Quality: High (Standard)]
d e
2.4
2.2
2.0
1.8
1.6
1.4
1.2
1.0
0.8
0.6
d i
0.6 0.8 1.0 1.2 1.4 1.6 1.8 2.0 2.2 2.4
Options
Type: di vs de
Range: Standard
Filter: By Element
Fingerprint Filter Options
Highlight close contacts from elements:
Inside: C
Outside: C
Include reciprocal contacts
Surface area included: 19.8 %
Save As...
Close

H-H: 6.6 %

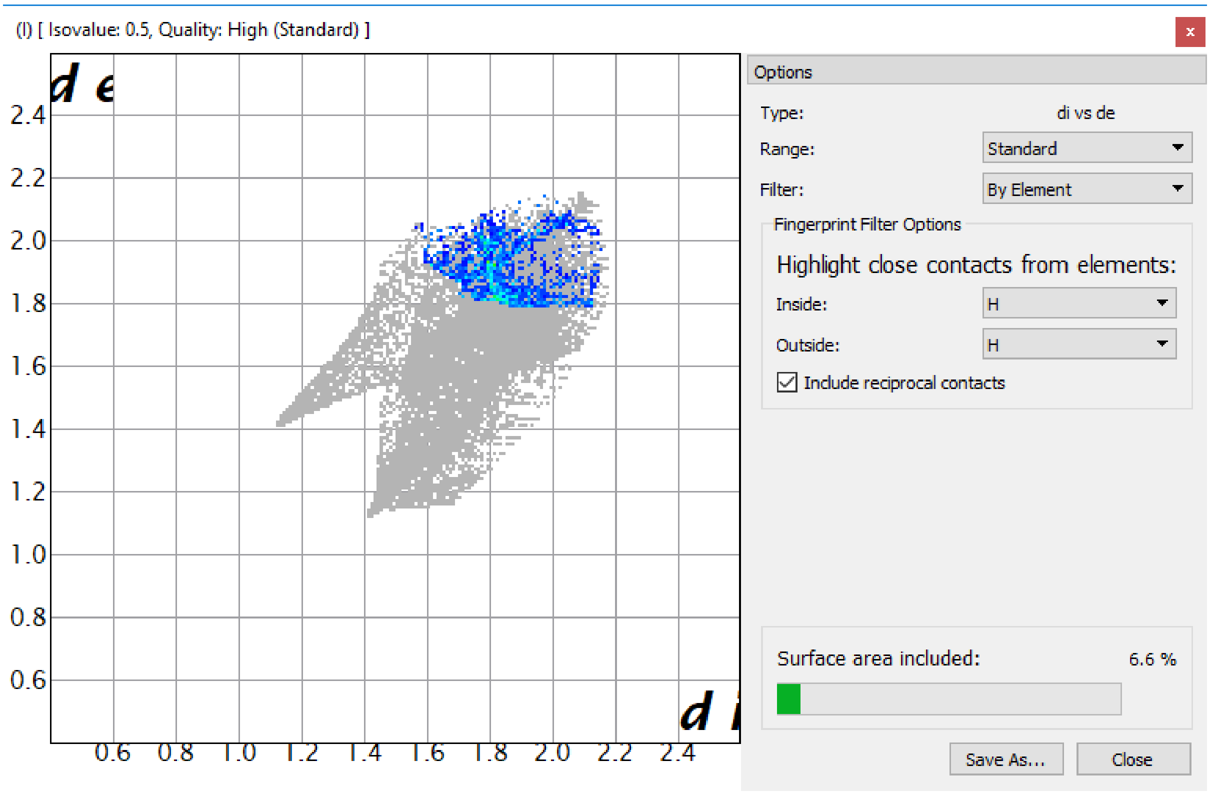
(I) [Isovalue: 0.5, Quality: High (Standard)]
d e
2.4
2.2
2.0
1.8
1.6
1.4
1.2
1.0
0.8
0.6
d i
0.6 0.8 1.0 1.2 1.4 1.6 1.8 2.0 2.2 2.4
Options
Type: di vs de
Range: Standard
Filter: By Element
Fingerprint Filter Options
Highlight close contacts from elements:
Inside: H
Outside: H
Include reciprocal contacts
Surface area included: 6.6 %
Save As...
Close

O-O: 9.5 %

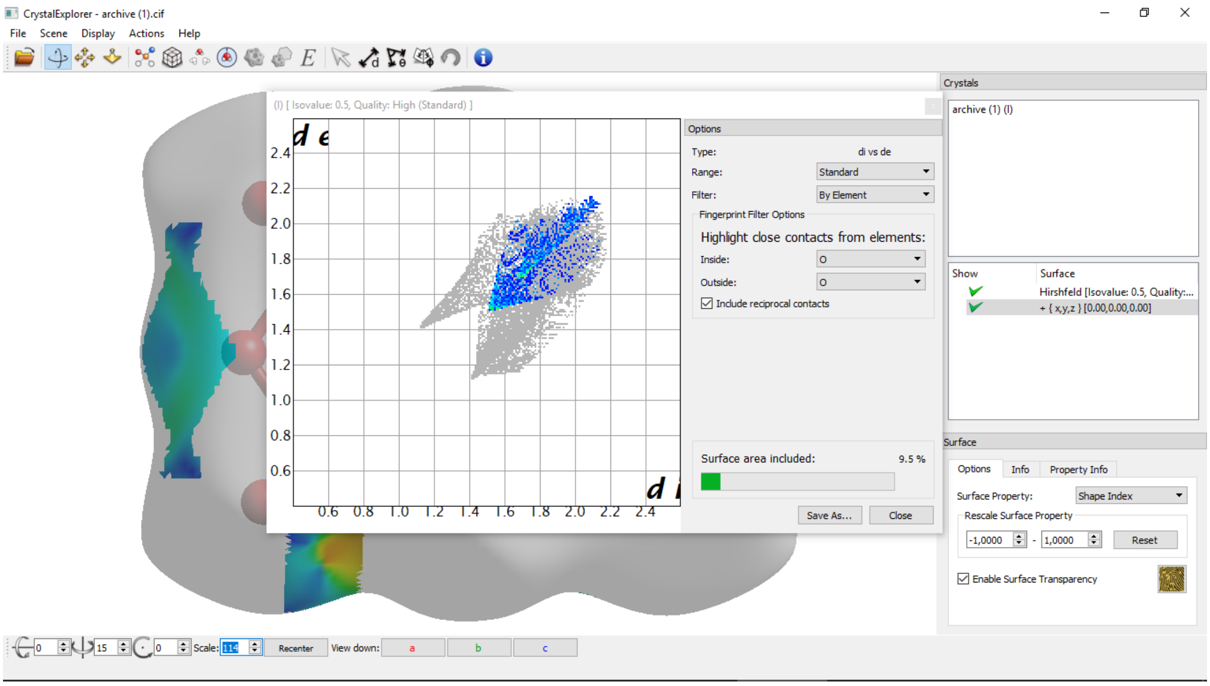
CrystalExplorer - archive (1).cif
File Scene Display Actions Help
(I) [Isovalue: 0.5, Quality: High (Standard)]
Options
Type: di vs de
Range: Standard
Filter: By Element
Fingerprint Filter Options
Highlight close contacts from elements:
Inside: O
Outside: O
Include reciprocal contacts
Surface area included: 9.5 %
Save As... Close
Crystals
archive (1) (I)
Show Surface
Hirshfeld [Isovalue: 0.5, Quality:...
+ { x,y,z } [0.00,0.00,0.00]
Surface
Options Info Property Info
Surface Property: Shape Index
Rescale Surface Property
-1,0000 - 1,0000 Reset
Enable Surface Transparency
Recenter View down: a b c

C-H: 3.9 %

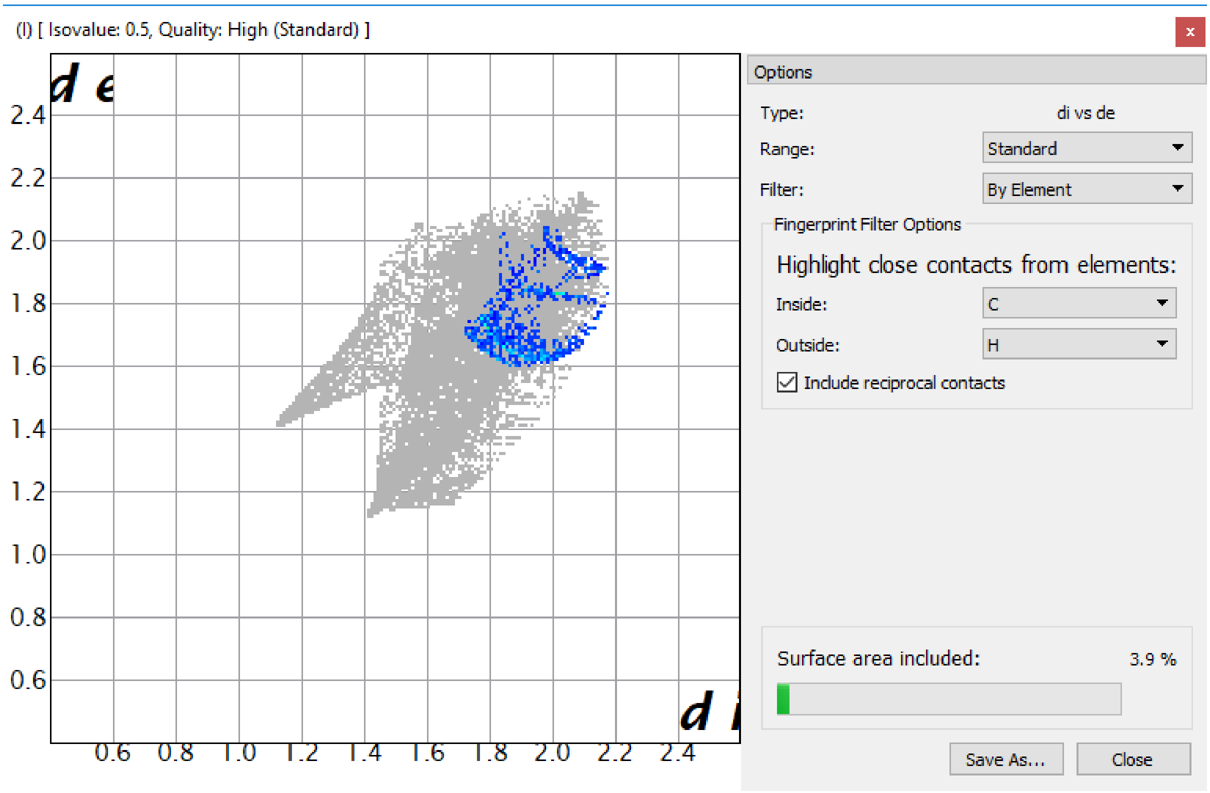
(I) [Isovalue: 0.5, Quality: High (Standard)]
Options
Type: di vs de
Range: Standard
Filter: By Element
Fingerprint Filter Options
Highlight close contacts from elements:
Inside: C
Outside: H
Include reciprocal contacts
Surface area included: 3.9 %
Save As... Close

C-O: 1.7 %

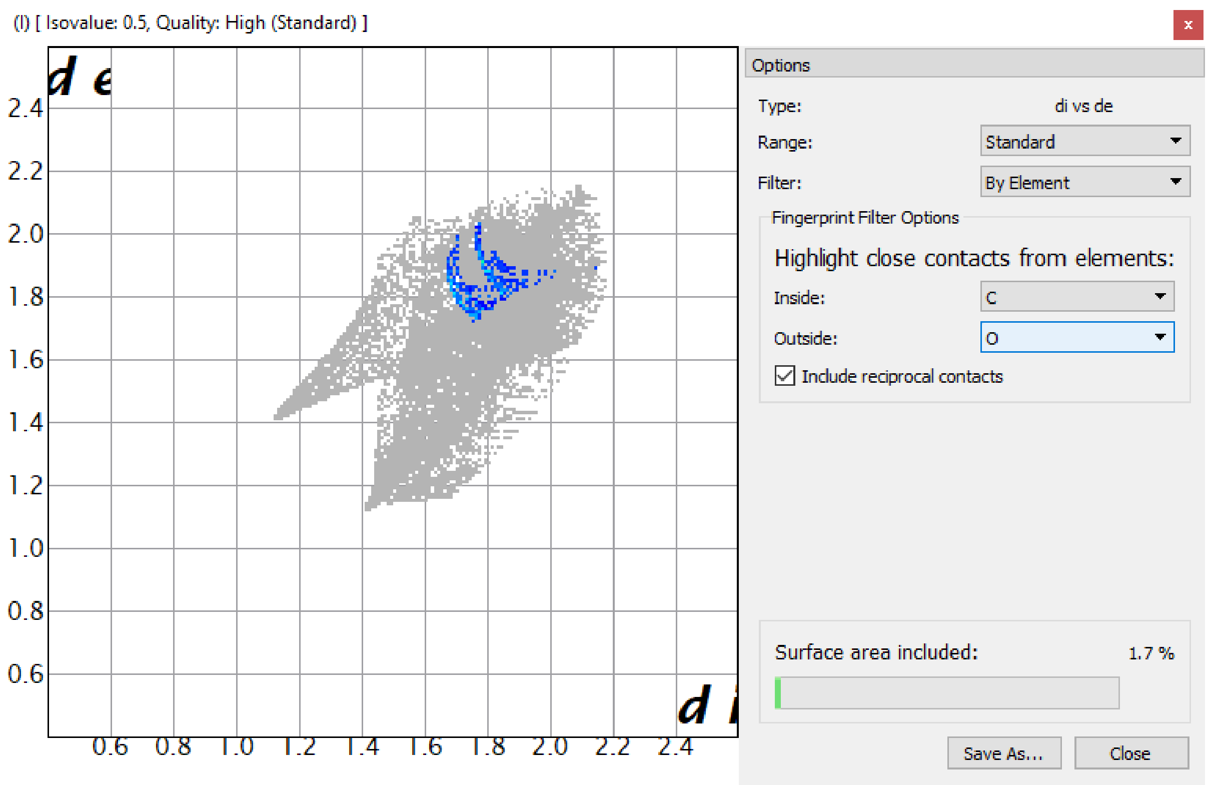
(I) [Isovalue: 0.5, Quality: High (Standard)]
d e
2.4
2.2
2.0
1.8
1.6
1.4
1.2
1.0
0.8
0.6
d i
0.6 0.8 1.0 1.2 1.4 1.6 1.8 2.0 2.2 2.4
Options
Type: di vs de
Range: Standard
Filter: By Element
Fingerprint Filter Options
Highlight close contacts from elements:
Inside: C
Outside: O
Include reciprocal contacts
Surface area included: 1.7 %
Save As...
Close

H-O: 58.4 %

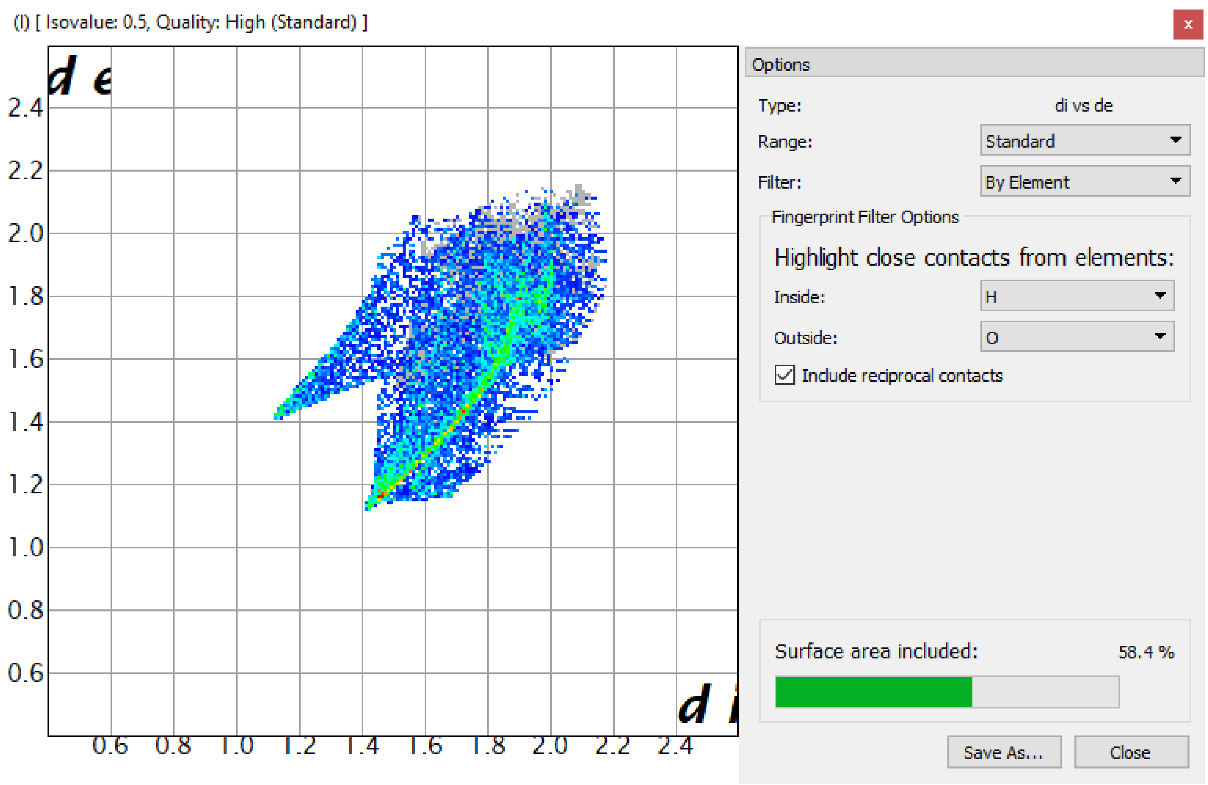
(I) [Isovalue: 0.5, Quality: High (Standard)]
d e
2.4
2.2
2.0
1.8
1.6
1.4
1.2
1.0
0.8
0.6
d i
0.6 0.8 1.0 1.2 1.4 1.6 1.8 2.0 2.2 2.4
Options
Type: di vs de
Range: Standard
Filter: By Element
Fingerprint Filter Options
Highlight close contacts from elements:
Inside: H
Outside: O
Include reciprocal contacts
Surface area included: 58.4 %
Save As...
Close

(II) pmda molecule 1 in ASU

ALL: 100 %

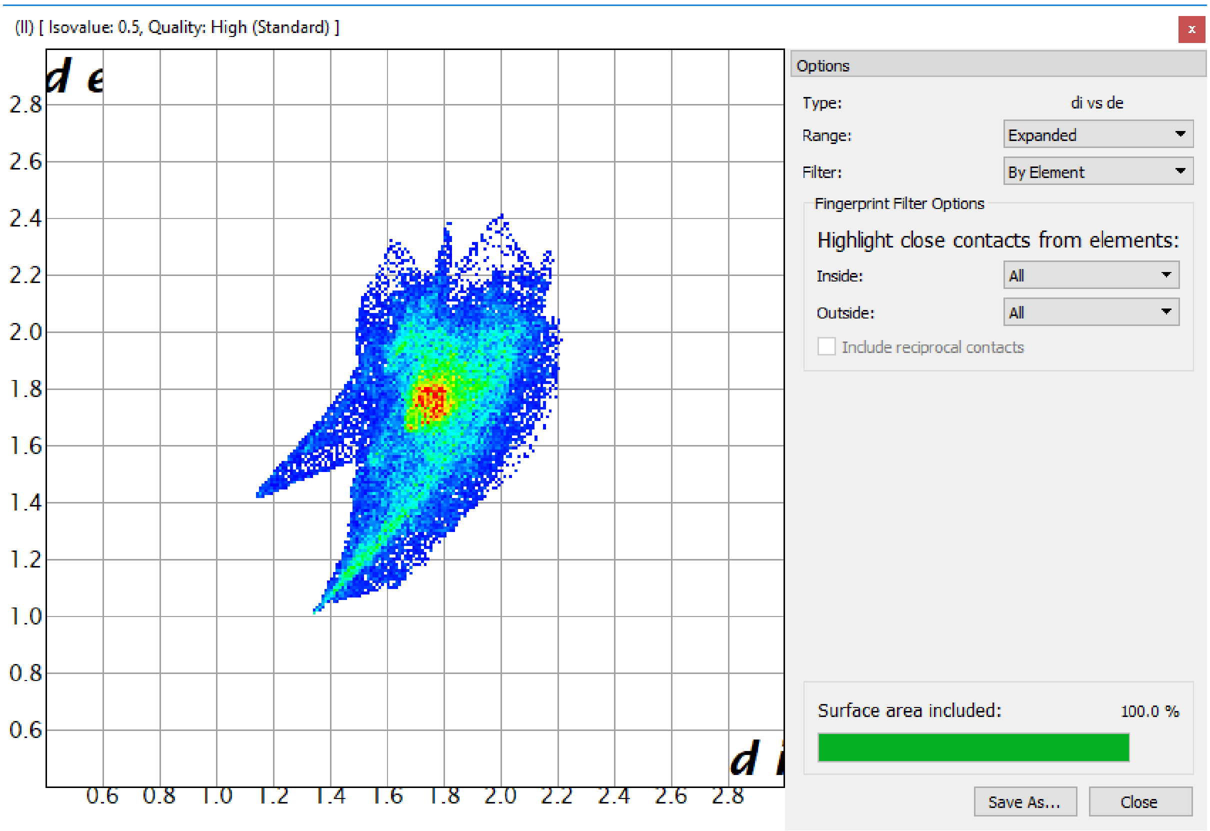

C-C: 21.0

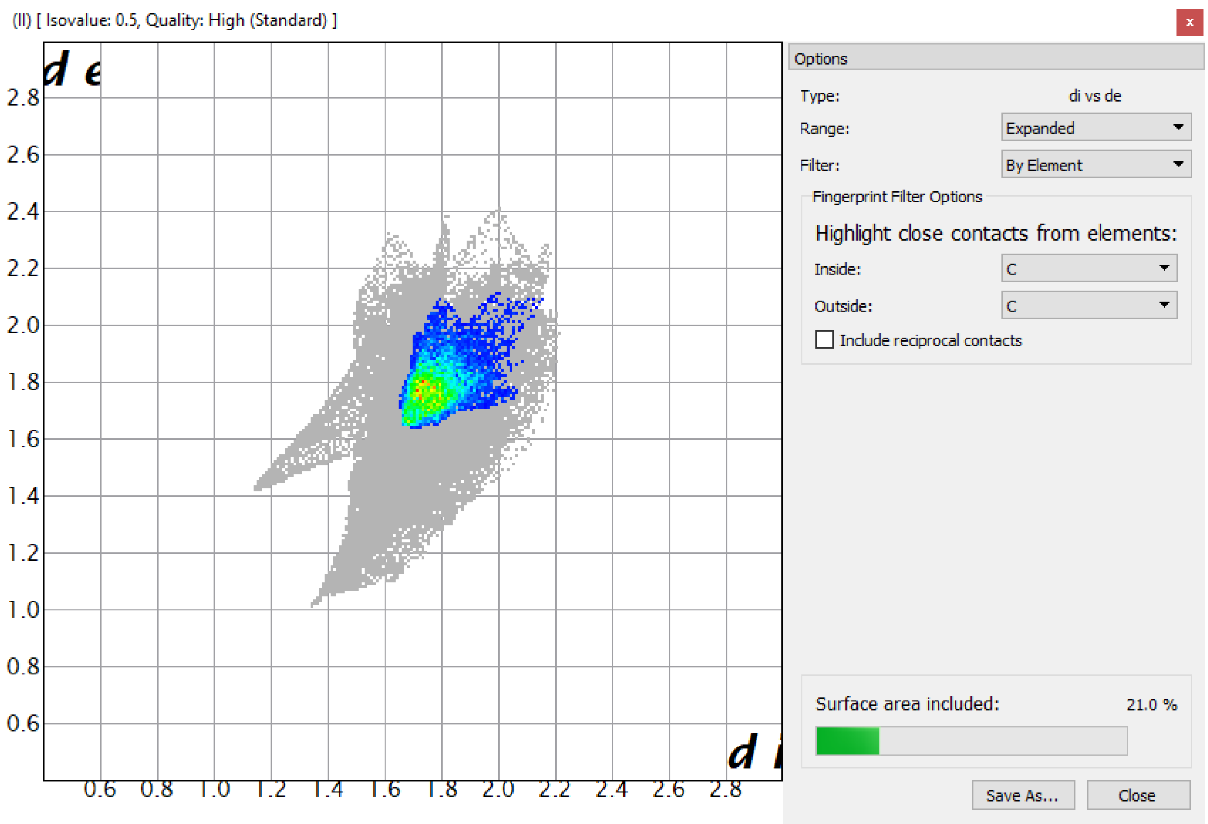
(II) [Isovalue: 0.5, Quality: High (Standard)]
d e
2.8
2.6
2.4
2.2
2.0
1.8
1.6
1.4
1.2
1.0
0.8
0.6
d i
0.6 0.8 1.0 1.2 1.4 1.6 1.8 2.0 2.2 2.4 2.6 2.8
Options
Type: di vs de
Range: Expanded
Filter: By Element
Fingerprint Filter Options
Highlight close contacts from elements:
Inside: C
Outside: C
Include reciprocal contacts
Surface area included: 21.0 %
Save As...
Close

H-H: 8.6 %

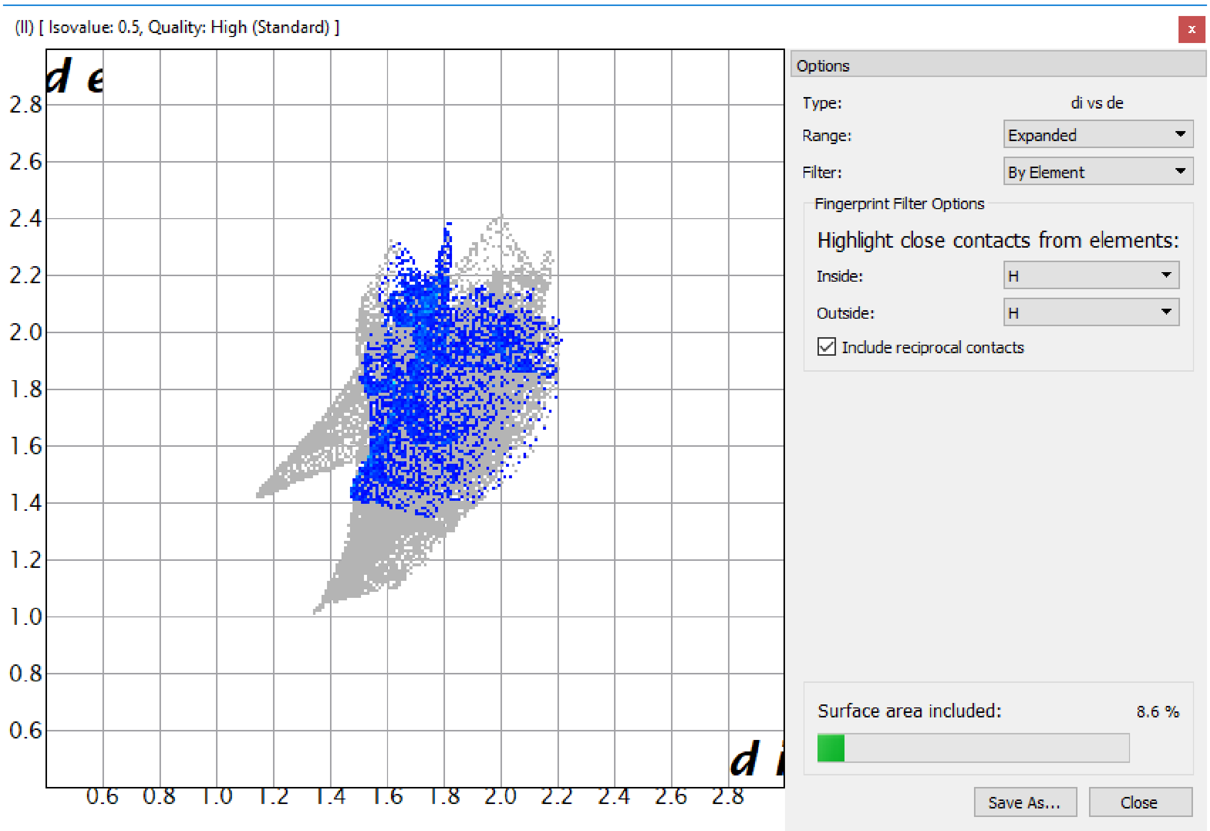
(II) [Isovalue: 0.5, Quality: High (Standard)]
d e
d i
0.6 0.8 1.0 1.2 1.4 1.6 1.8 2.0 2.2 2.4 2.6 2.8
Options
Type: di vs de
Range: Expanded
Filter: By Element
Fingerprint Filter Options
Highlight close contacts from elements:
Inside: H
Outside: H
Include reciprocal contacts
Surface area included: 8.6 %
Save As...
Close

O-O: 5.5 %

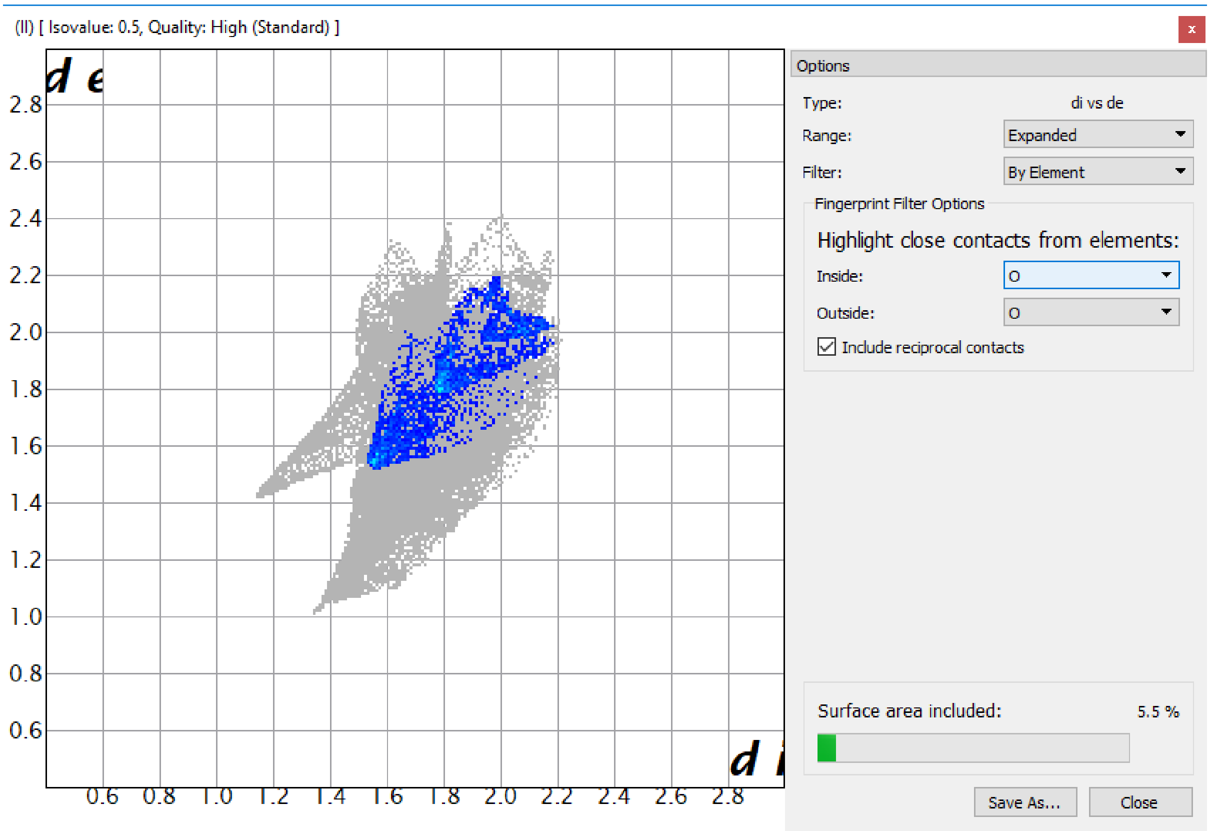
(II) [Isovalue: 0.5, Quality: High (Standard)]
d e
d i
0.6 0.8 1.0 1.2 1.4 1.6 1.8 2.0 2.2 2.4 2.6 2.8
Options
Type: di vs de
Range: Expanded
Filter: By Element
Fingerprint Filter Options
Highlight close contacts from elements:
Inside: O
Outside: O
Include reciprocal contacts
Surface area included: 5.5 %
Save As...
Close

C-H: 5.4 %

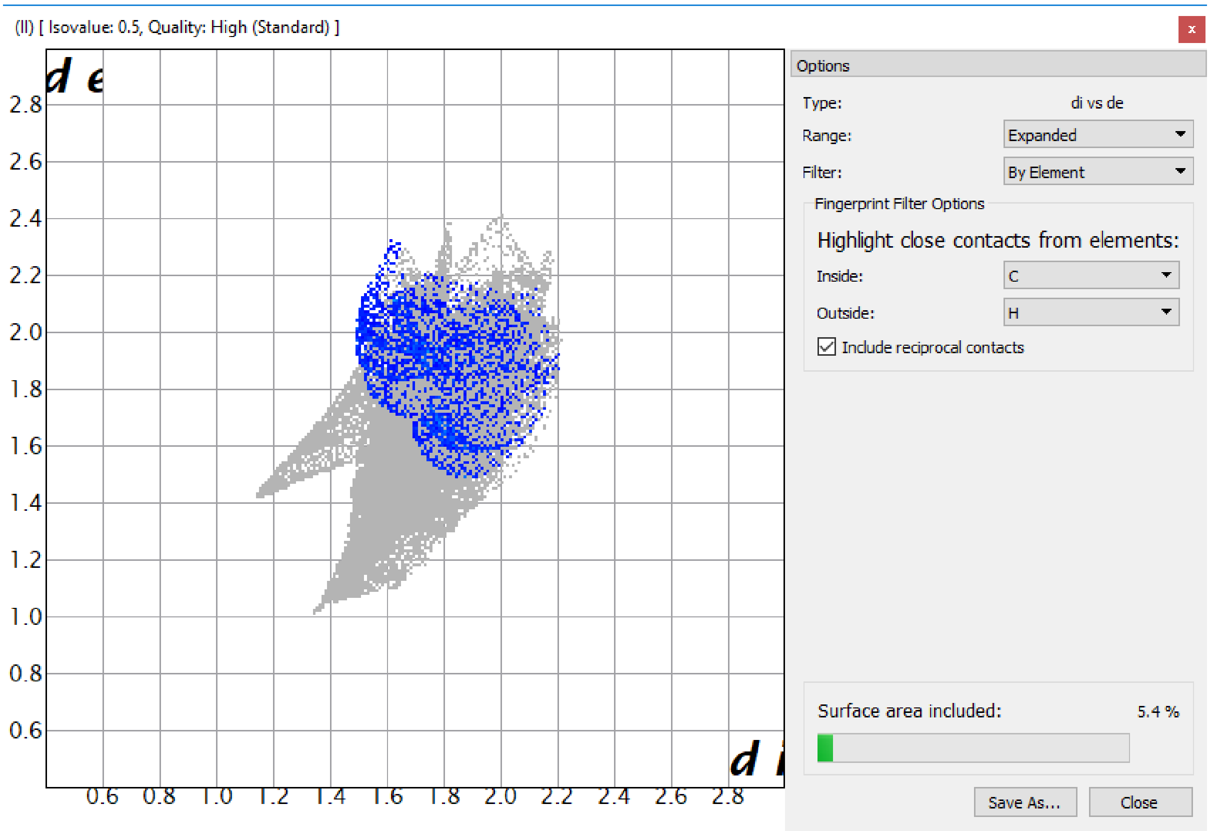
(II) [Isovalue: 0.5, Quality: High (Standard)]
d e
2.8
2.6
2.4
2.2
2.0
1.8
1.6
1.4
1.2
1.0
0.8
0.6
d i
0.6 0.8 1.0 1.2 1.4 1.6 1.8 2.0 2.2 2.4 2.6 2.8
Options
Type: di vs de
Range: Expanded
Filter: By Element
Fingerprint Filter Options
Highlight close contacts from elements:
Inside: C
Outside: H
Include reciprocal contacts
Surface area included: 5.4 %
Save As...
Close

C-O: 6.6 %

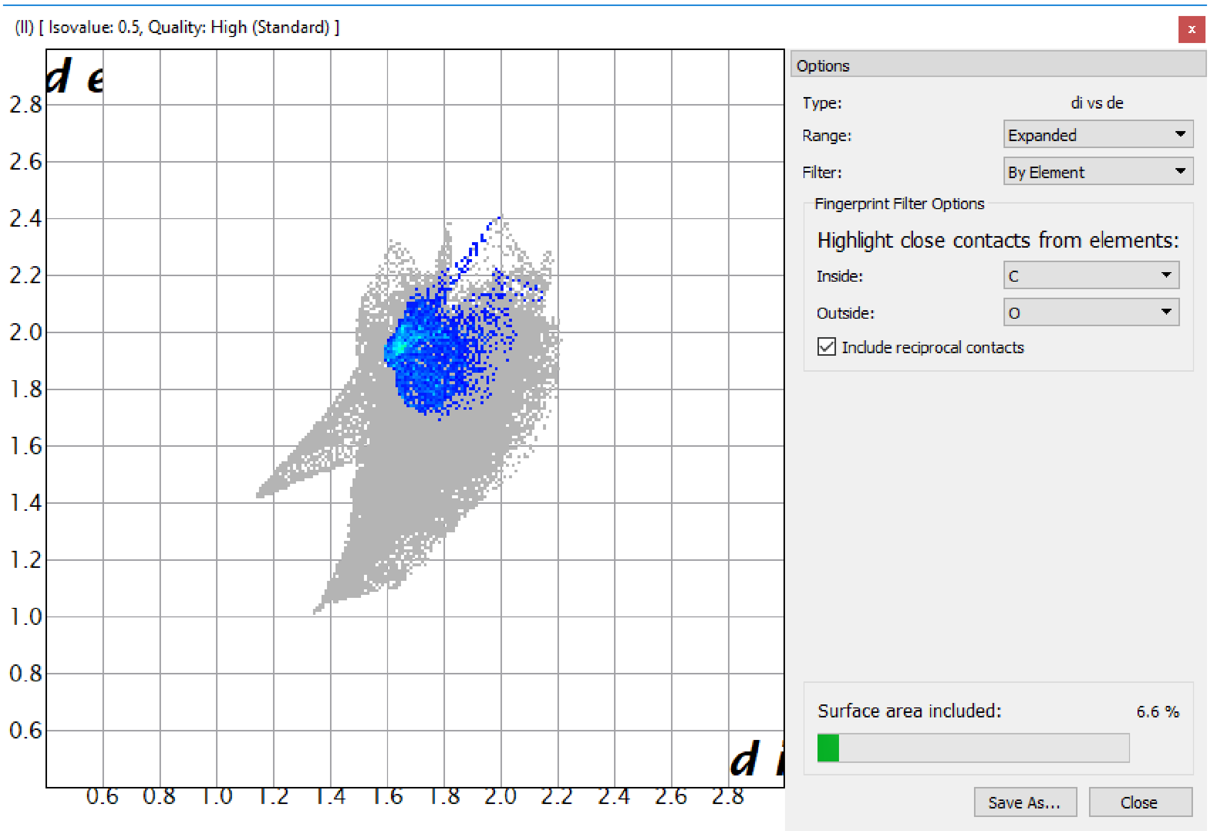
(II) [Isovalue: 0.5, Quality: High (Standard)]
d e
2.8
2.6
2.4
2.2
2.0
1.8
1.6
1.4
1.2
1.0
0.8
0.6
d i
0.6 0.8 1.0 1.2 1.4 1.6 1.8 2.0 2.2 2.4 2.6 2.8
Options
Type: di vs de
Range: Expanded
Filter: By Element
Fingerprint Filter Options
Highlight close contacts from elements:
Inside: C
Outside: O
Include reciprocal contacts
Surface area included: 6.6 %
Save As...
Close

H-O: 52.8 %

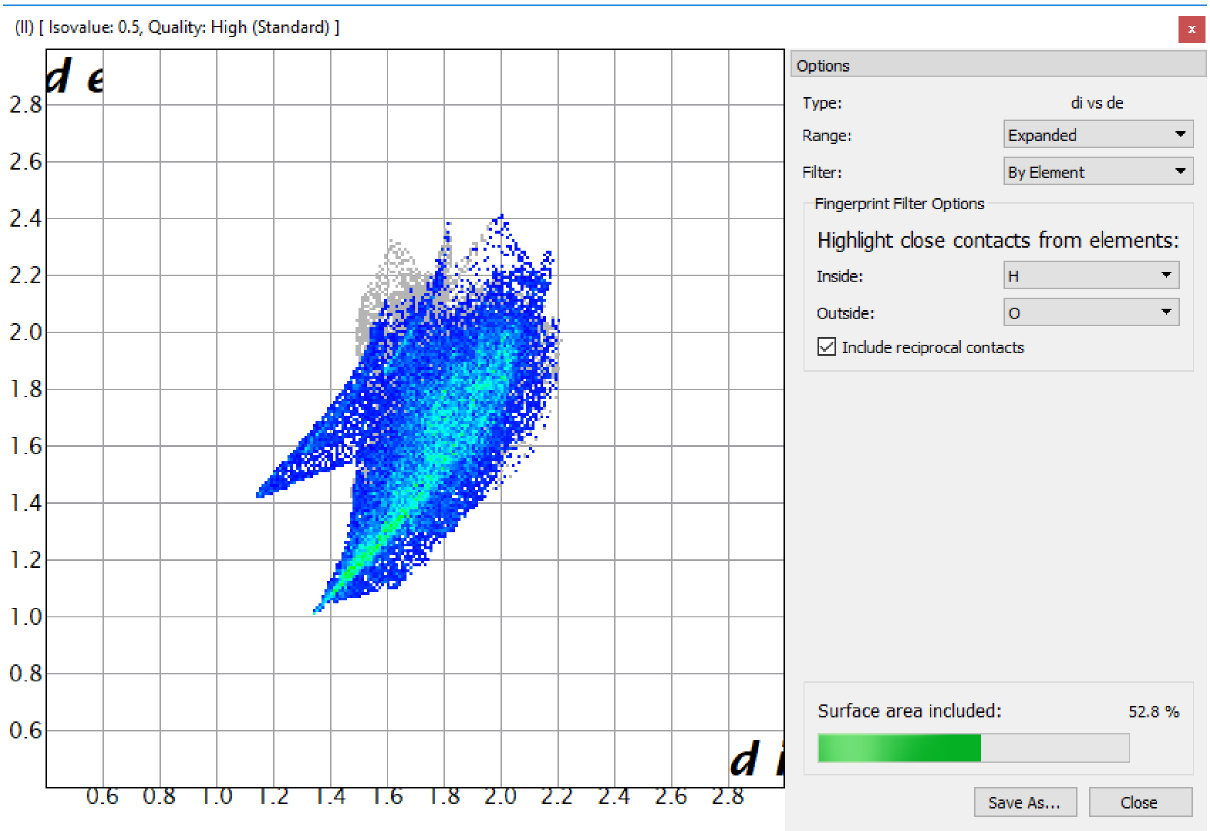

(II) [Isovalue: 0.5, Quality: High (Standard)]
d e
2.8
2.6
2.4
2.2
2.0
1.8
1.6
1.4
1.2
1.0
0.8
0.6
d i
0.6 0.8 1.0 1.2 1.4 1.6 1.8 2.0 2.2 2.4 2.6 2.8
Options
Type: di vs de
Range: Expanded
Filter: By Element
Fingerprint Filter Options
Highlight close contacts from elements:
Inside: H
Outside: O
Include reciprocal contacts
Surface area included: 52.8 %
Save As...
Close

(II) pmda molecule 2 in ASU

ALL: 100%

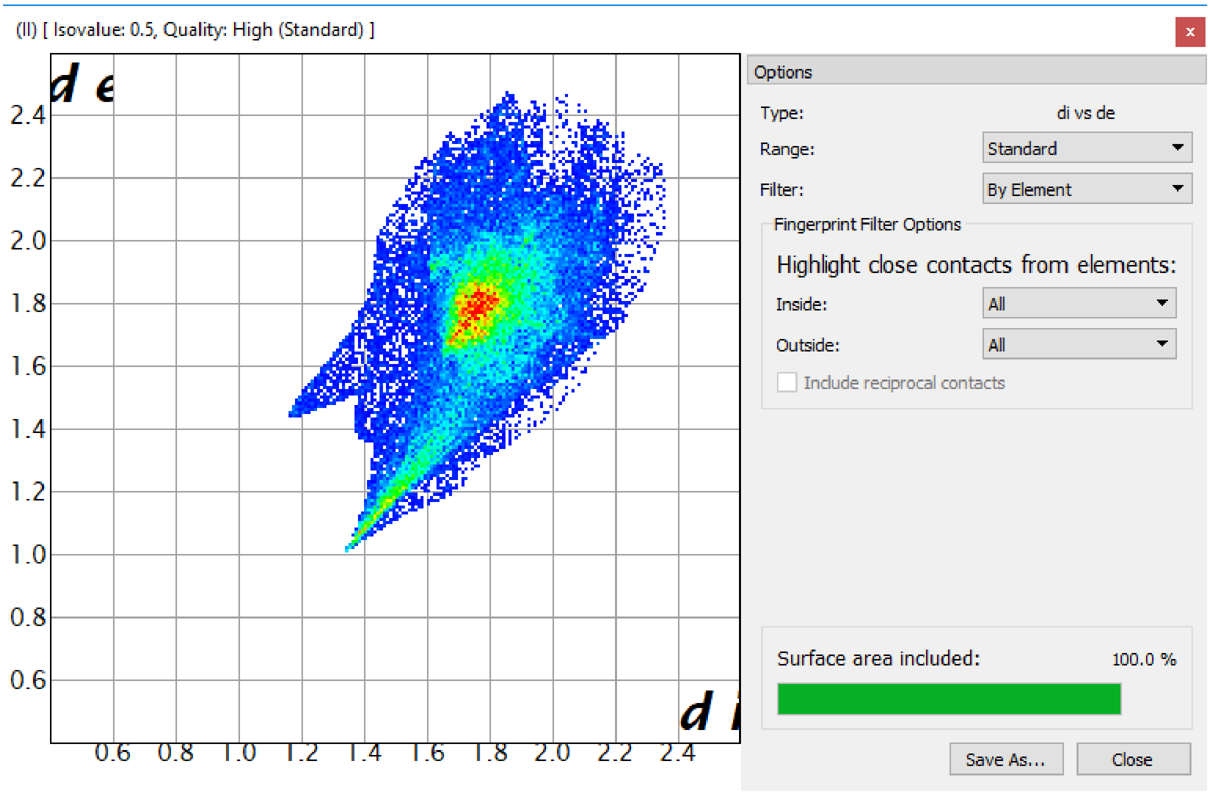

C-C: 20.6 %

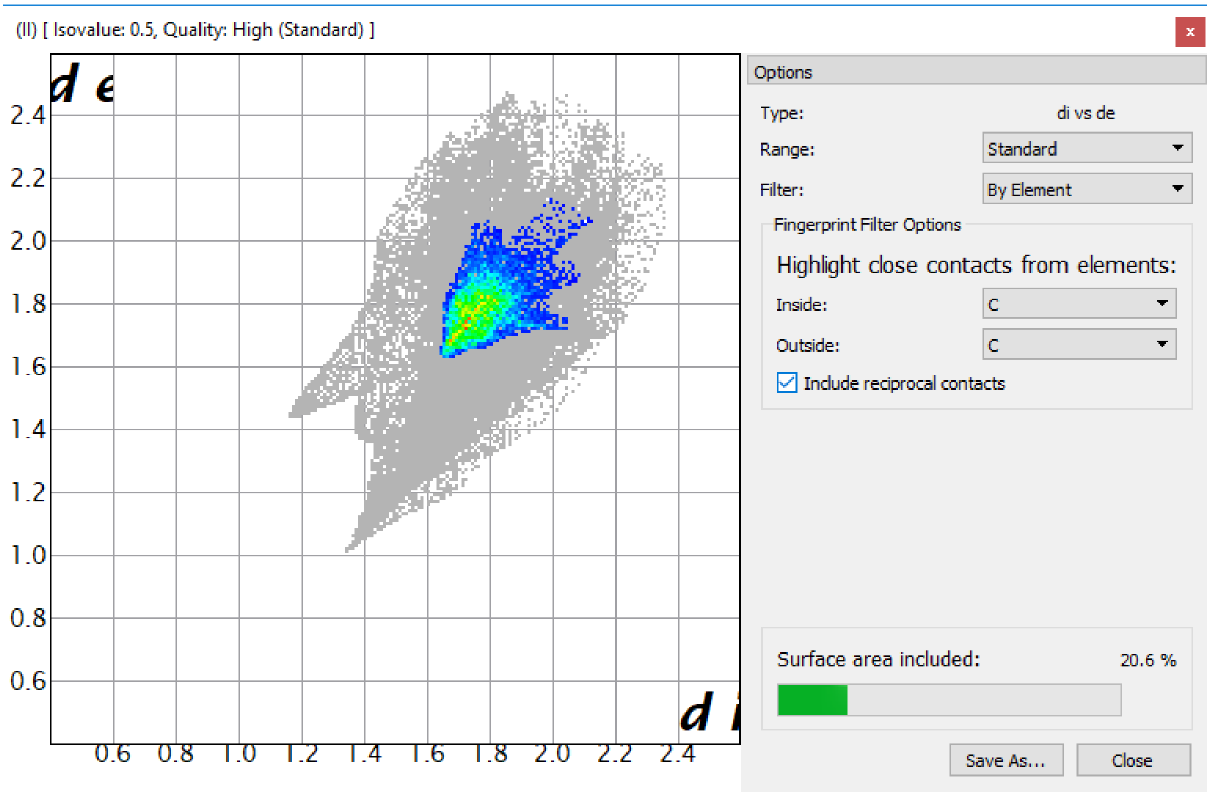
(II) [Isovalue: 0.5, Quality: High (Standard)]
d e
2.4
2.2
2.0
1.8
1.6
1.4
1.2
1.0
0.8
0.6
d i
0.6 0.8 1.0 1.2 1.4 1.6 1.8 2.0 2.2 2.4
Options
Type: di vs de
Range: Standard
Filter: By Element
Fingerprint Filter Options
Highlight close contacts from elements:
Inside: C
Outside: C
Include reciprocal contacts
Surface area included: 20.6 %
Save As...
Close

H-H: 11.7 %

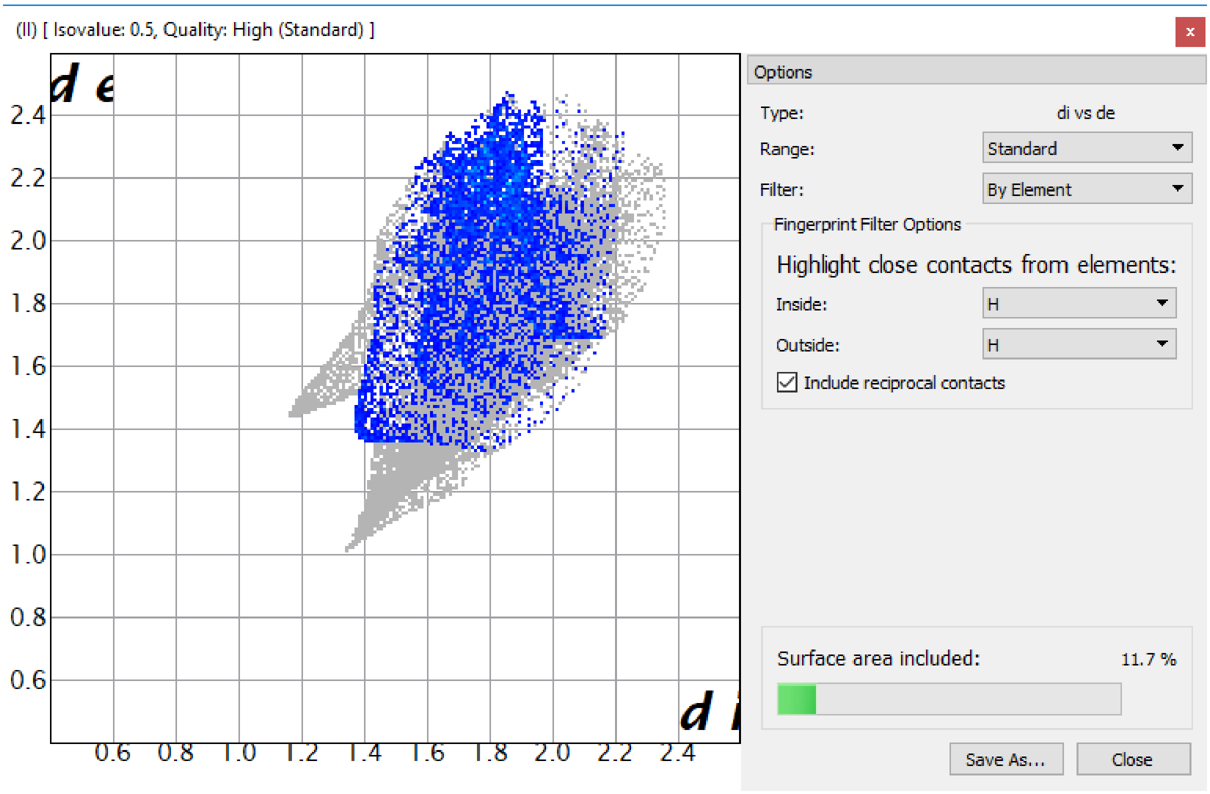
(II) [Isovalue: 0.5, Quality: High (Standard)]
d e
2.4
2.2
2.0
1.8
1.6
1.4
1.2
1.0
0.8
0.6
d i
0.6 0.8 1.0 1.2 1.4 1.6 1.8 2.0 2.2 2.4
Options
Type: di vs de
Range: Standard
Filter: By Element
Fingerprint Filter Options
Highlight close contacts from elements:
Inside: H
Outside: H
Include reciprocal contacts
Surface area included: 11.7 %
Save As...
Close

O-O: 7.1 %

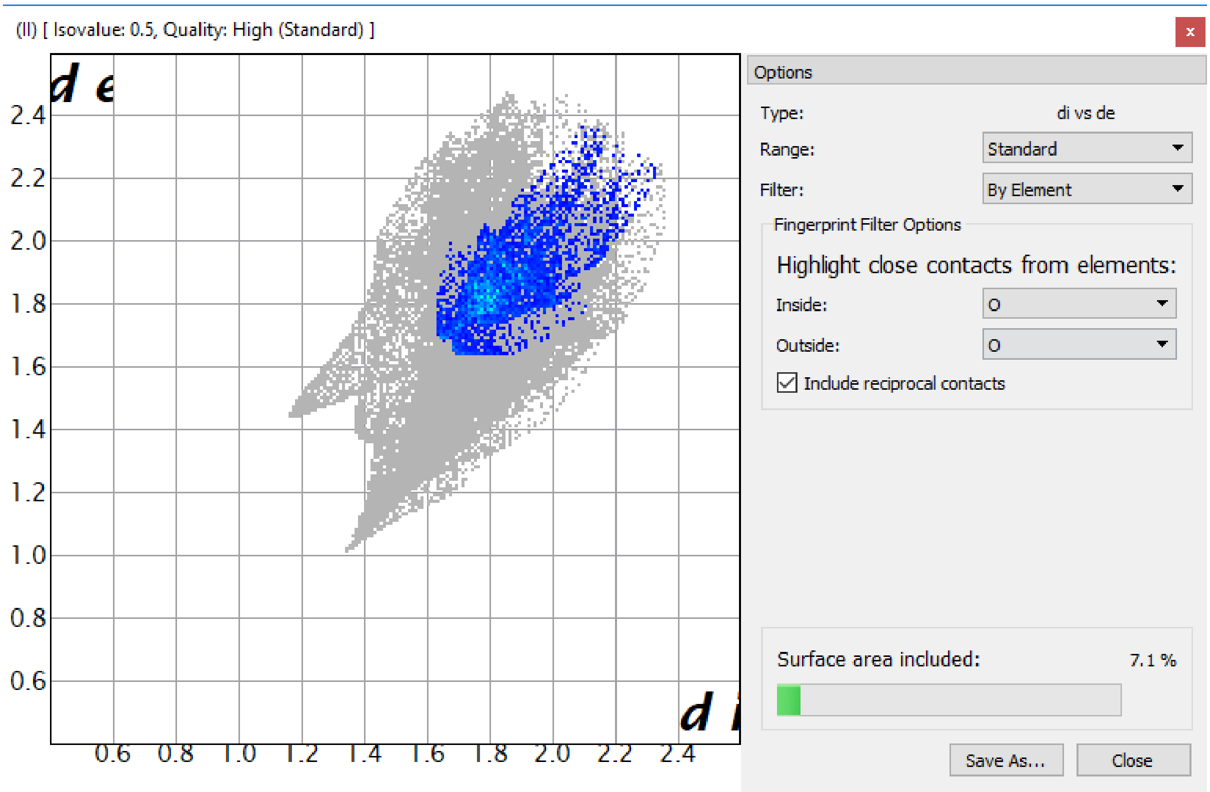
(II) [Isovalue: 0.5, Quality: High (Standard)]
d e
2.4
2.2
2.0
1.8
1.6
1.4
1.2
1.0
0.8
0.6
d i
0.6 0.8 1.0 1.2 1.4 1.6 1.8 2.0 2.2 2.4
Options
Type: di vs de
Range: Standard
Filter: By Element
Fingerprint Filter Options
Highlight close contacts from elements:
Inside: O
Outside: O
Include reciprocal contacts
Surface area included: 7.1 %
Save As...
Close

C-H: 6.2 %

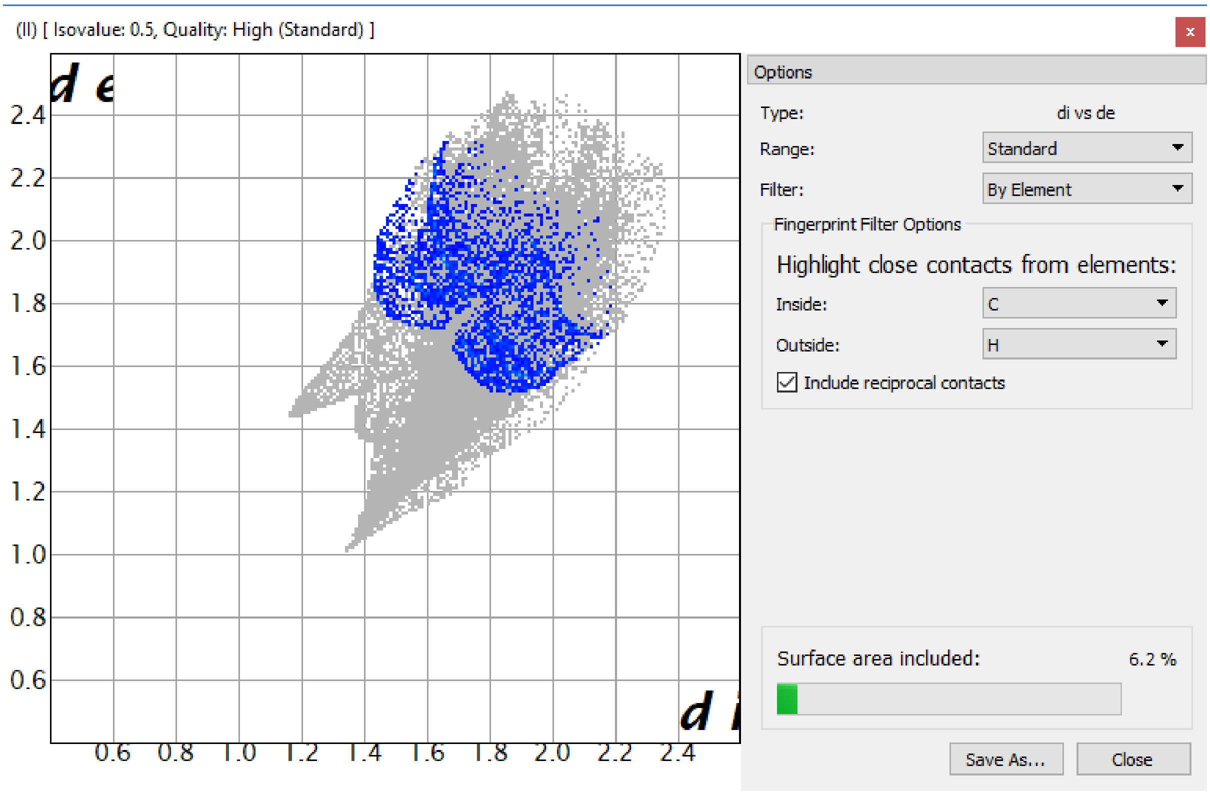
(II) [Isovalue: 0.5, Quality: High (Standard)]
d e
2.4
2.2
2.0
1.8
1.6
1.4
1.2
1.0
0.8
0.6
d i
0.6 0.8 1.0 1.2 1.4 1.6 1.8 2.0 2.2 2.4
Options
Type: di vs de
Range: Standard
Filter: By Element
Fingerprint Filter Options
Highlight close contacts from elements:
Inside: C
Outside: H
Include reciprocal contacts
Surface area included: 6.2 %
Save As...
Close

C-O: 5.9 %

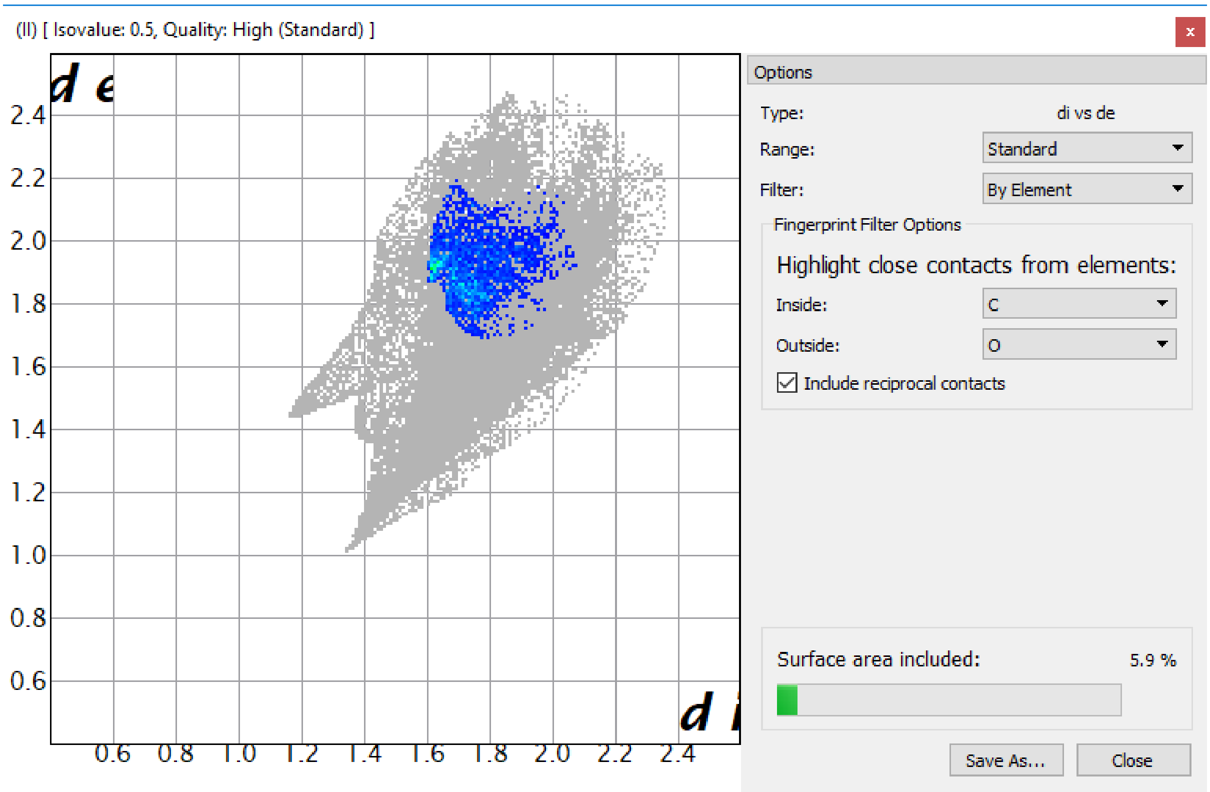

(II) [Isovalue: 0.5, Quality: High (Standard)]
d e
2.4
2.2
2.0
1.8
1.6
1.4
1.2
1.0
0.8
0.6
d i
0.6 0.8 1.0 1.2 1.4 1.6 1.8 2.0 2.2 2.4
Options
Type: di vs de
Range: Standard
Filter: By Element
Fingerprint Filter Options
Highlight close contacts from elements:
Inside: C
Outside: O
Include reciprocal contacts
Surface area included: 5.9 %
Save As...
Close

H-O: 48.5 %

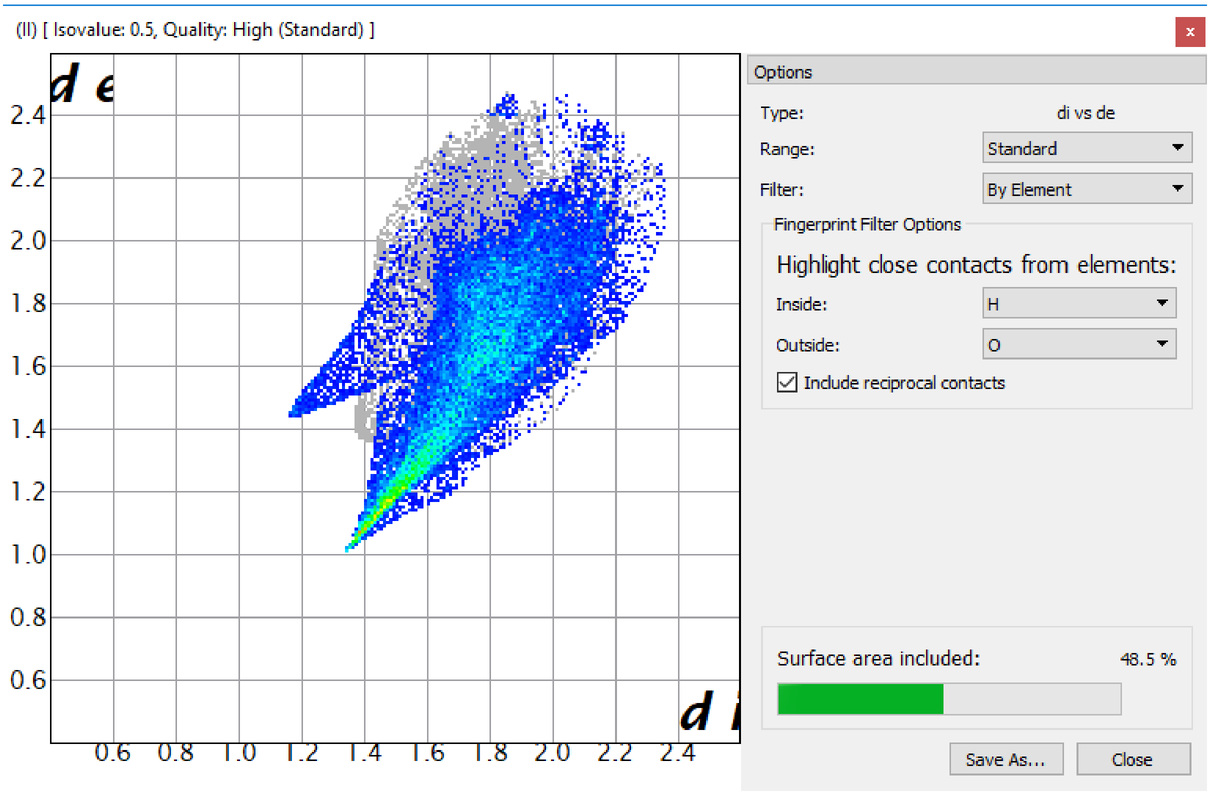

(II) [Isovalue: 0.5, Quality: High (Standard)]
d e
2.4
2.2
2.0
1.8
1.6
1.4
1.2
1.0
0.8
0.6
d i
0.6 0.8 1.0 1.2 1.4 1.6 1.8 2.0 2.2 2.4
Options
Type: di vs de
Range: Standard
Filter: By Element
Fingerprint Filter Options
Highlight close contacts from elements:
Inside: H
Outside: O
Include reciprocal contacts
Surface area included: 48.5 %
Save As...
Close

(III)

ALL: 100%

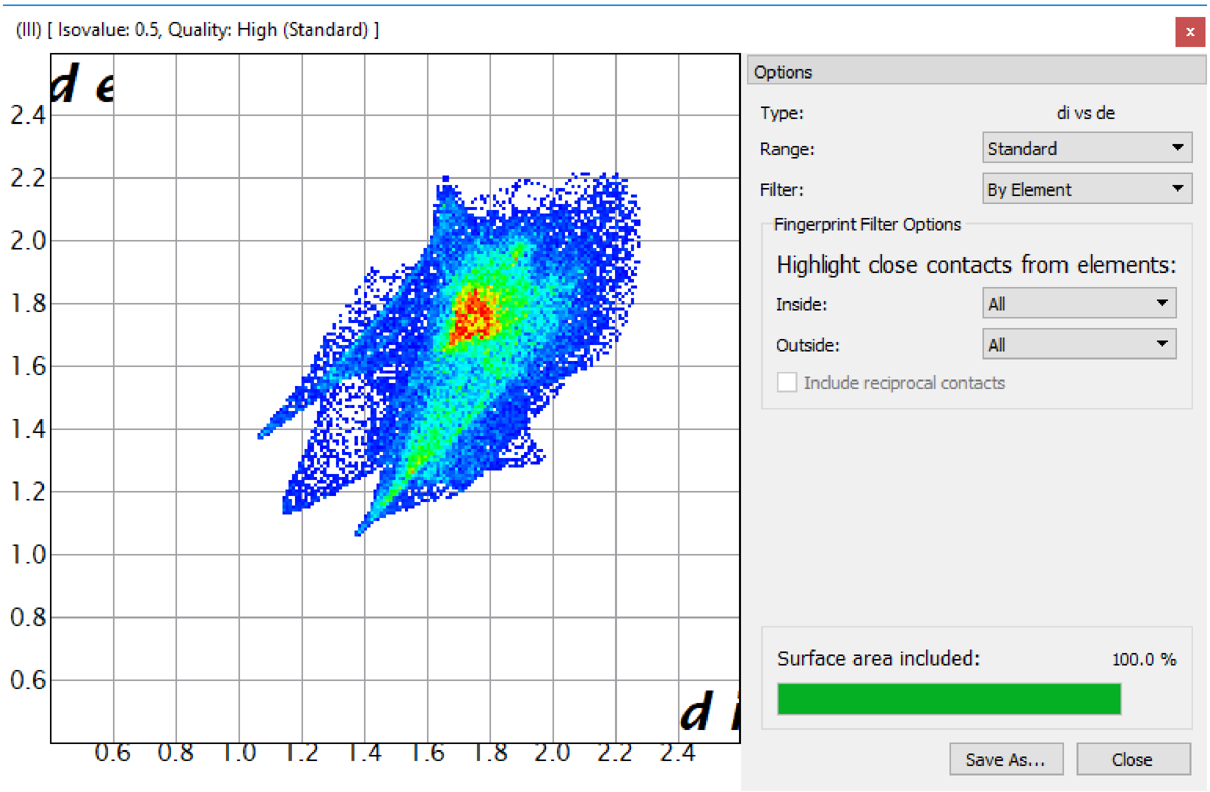
(III) [Isovalue: 0.5, Quality: High (Standard)]
d e
2.4
2.2
2.0
1.8
1.6
1.4
1.2
1.0
0.8
0.6
d i
0.6 0.8 1.0 1.2 1.4 1.6 1.8 2.0 2.2 2.4
Options
Type: di vs de
Range: Standard
Filter: By Element
Fingerprint Filter Options
Highlight close contacts from elements:
Inside: All
Outside: All
Include reciprocal contacts
Surface area included: 100.0 %
Save As...
Close

C-C: 20.2%

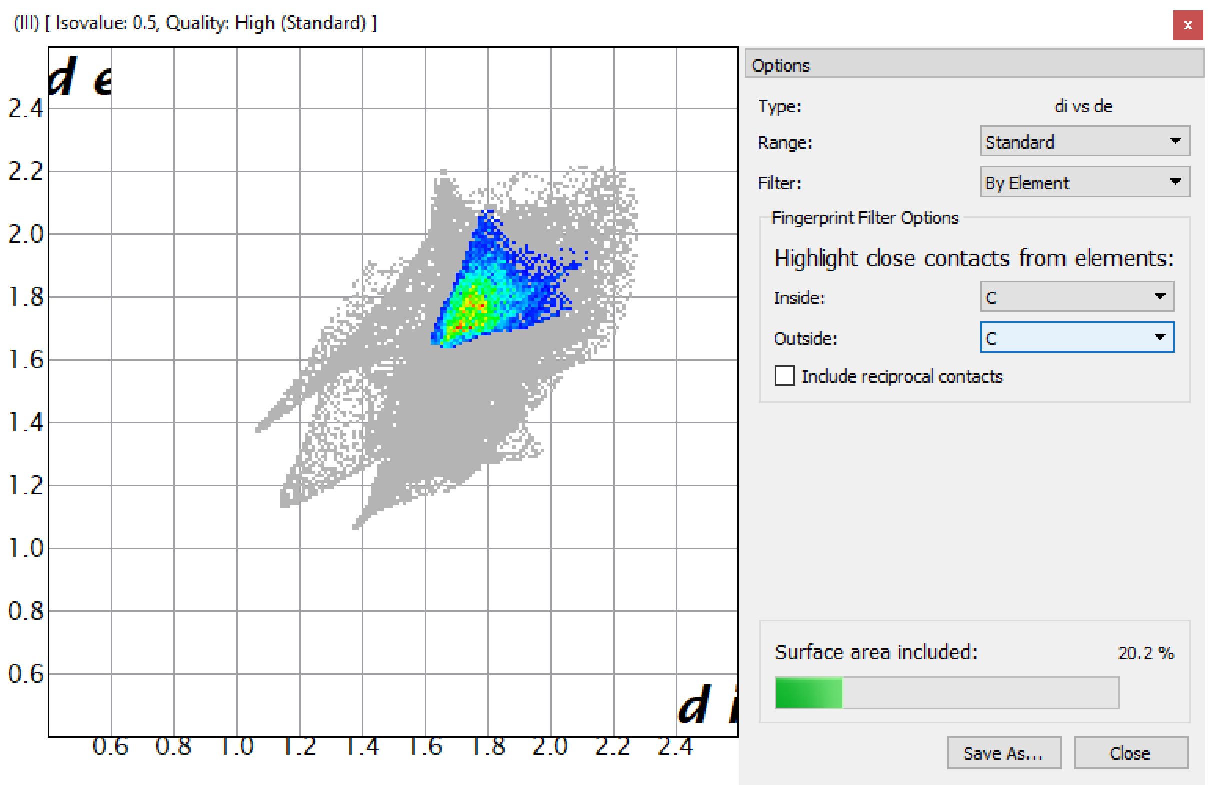
(III) [Isovalue: 0.5, Quality: High (Standard)]
de
di
2.4
2.2
2.0
1.8
1.6
1.4
1.2
1.0
0.8
0.6
0.6 0.8 1.0 1.2 1.4 1.6 1.8 2.0 2.2 2.4
Options
Type: di vs de
Range: Standard
Filter: By Element
Fingerprint Filter Options
Highlight close contacts from elements:
Inside: C
Outside: C
Include reciprocal contacts
Surface area included: 20.2 %
Save As...
Close

H-H: 9.5%

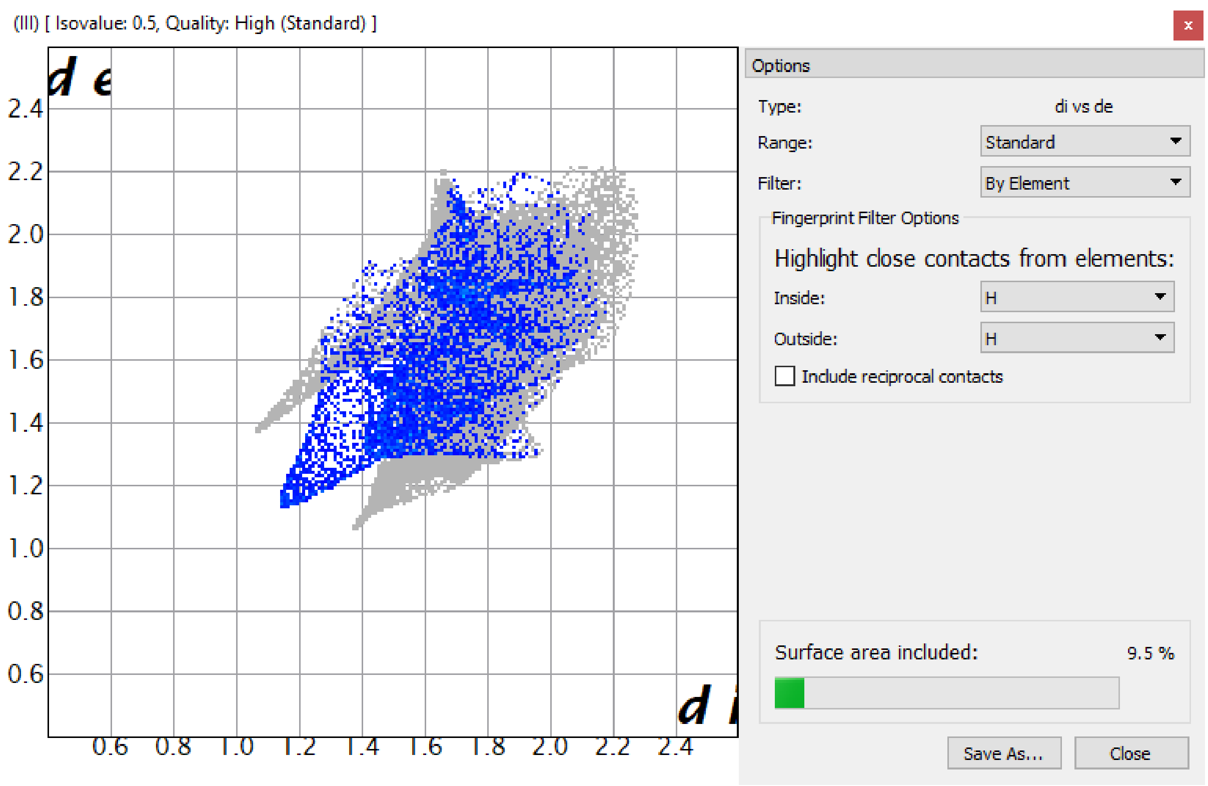
(III) [Isovalue: 0.5, Quality: High (Standard)]
de
di
2.4
2.2
2.0
1.8
1.6
1.4
1.2
1.0
0.8
0.6
0.6 0.8 1.0 1.2 1.4 1.6 1.8 2.0 2.2 2.4
Options
Type: di vs de
Range: Standard
Filter: By Element
Fingerprint Filter Options
Highlight close contacts from elements:
Inside: H
Outside: H
Include reciprocal contacts
Surface area included: 9.5 %
Save As...
Close

O-O: 4.2%

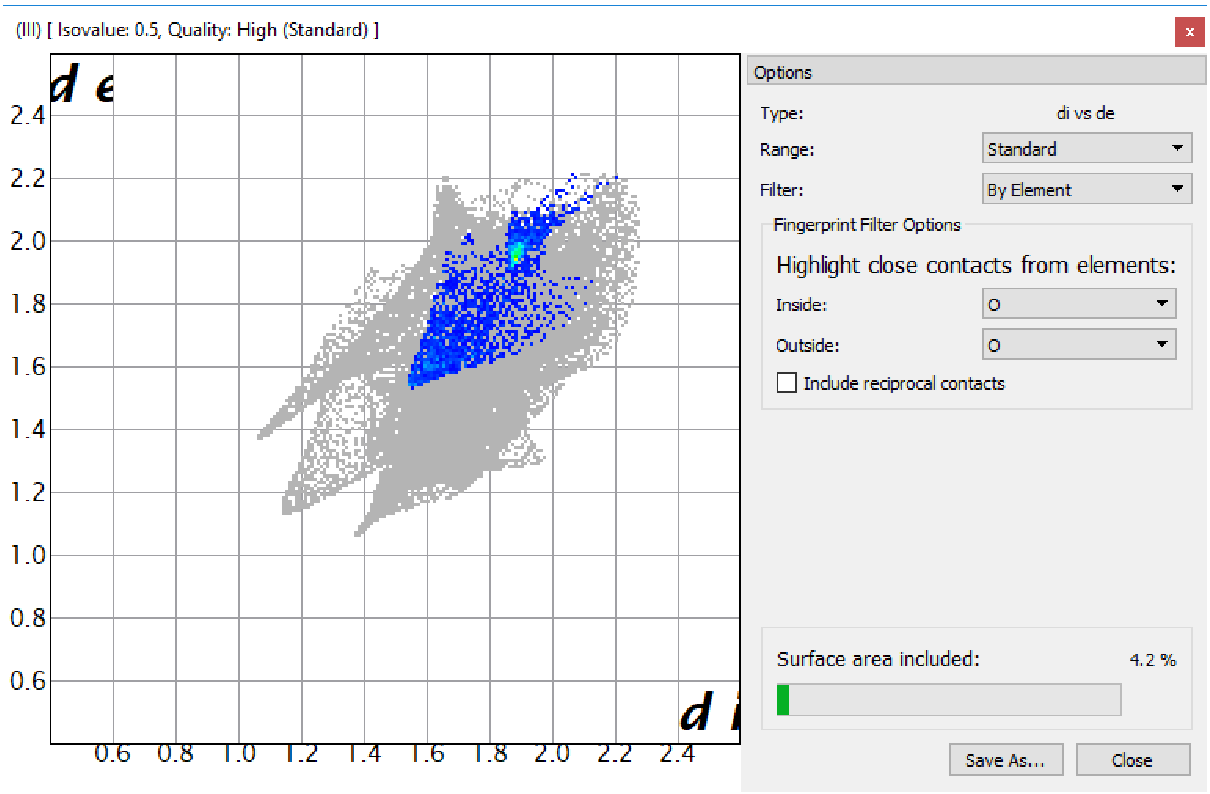
(III) [Isovalue: 0.5, Quality: High (Standard)]
d e
2.4
2.2
2.0
1.8
1.6
1.4
1.2
1.0
0.8
0.6
d i
0.6 0.8 1.0 1.2 1.4 1.6 1.8 2.0 2.2 2.4
Options
Type: di vs de
Range: Standard
Filter: By Element
Fingerprint Filter Options
Highlight close contacts from elements:
Inside: O
Outside: O
Include reciprocal contacts
Surface area included: 4.2 %
Save As...
Close

C-H: 4.1 %

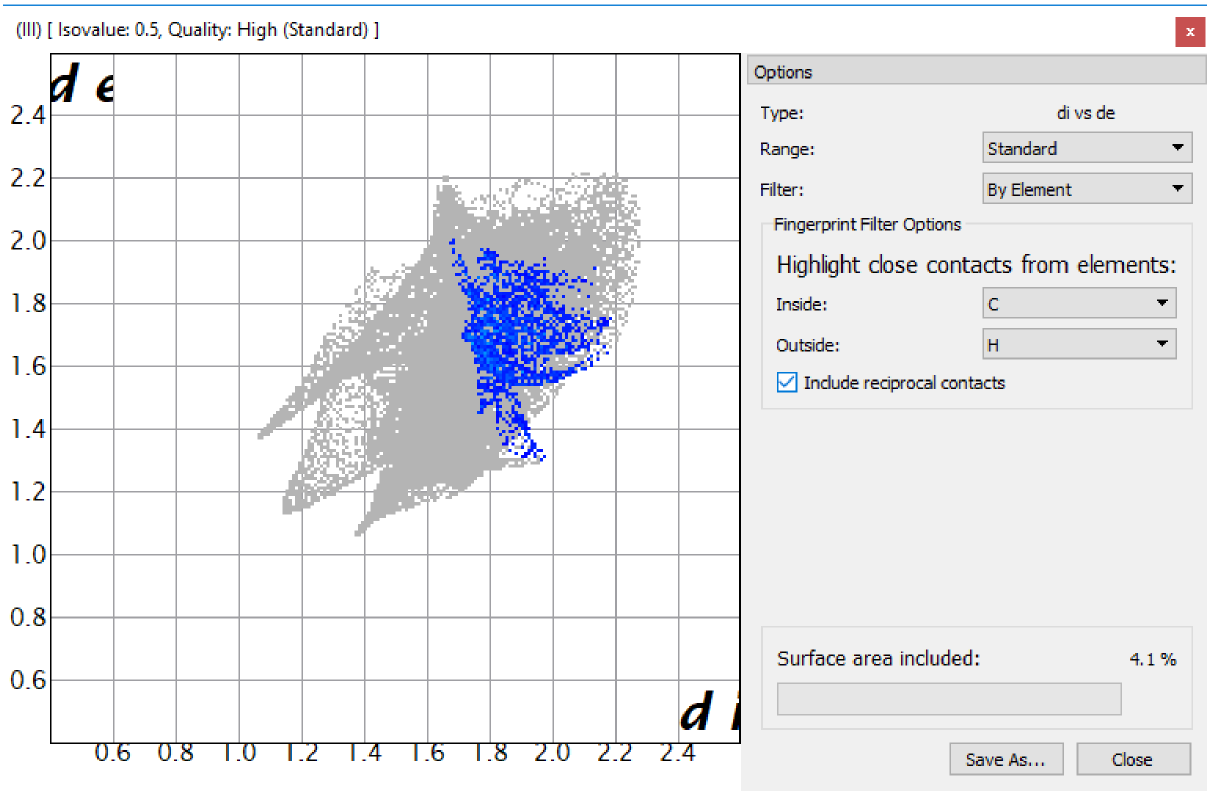
(III) [Isovalue: 0.5, Quality: High (Standard)]
d e
2.4
2.2
2.0
1.8
1.6
1.4
1.2
1.0
0.8
0.6
d i
0.6 0.8 1.0 1.2 1.4 1.6 1.8 2.0 2.2 2.4
Options
Type: di vs de
Range: Standard
Filter: By Element
Fingerprint Filter Options
Highlight close contacts from elements:
Inside: C
Outside: H
Include reciprocal contacts
Surface area included: 4.1 %
Save As...
Close

C-O: 5.2 %

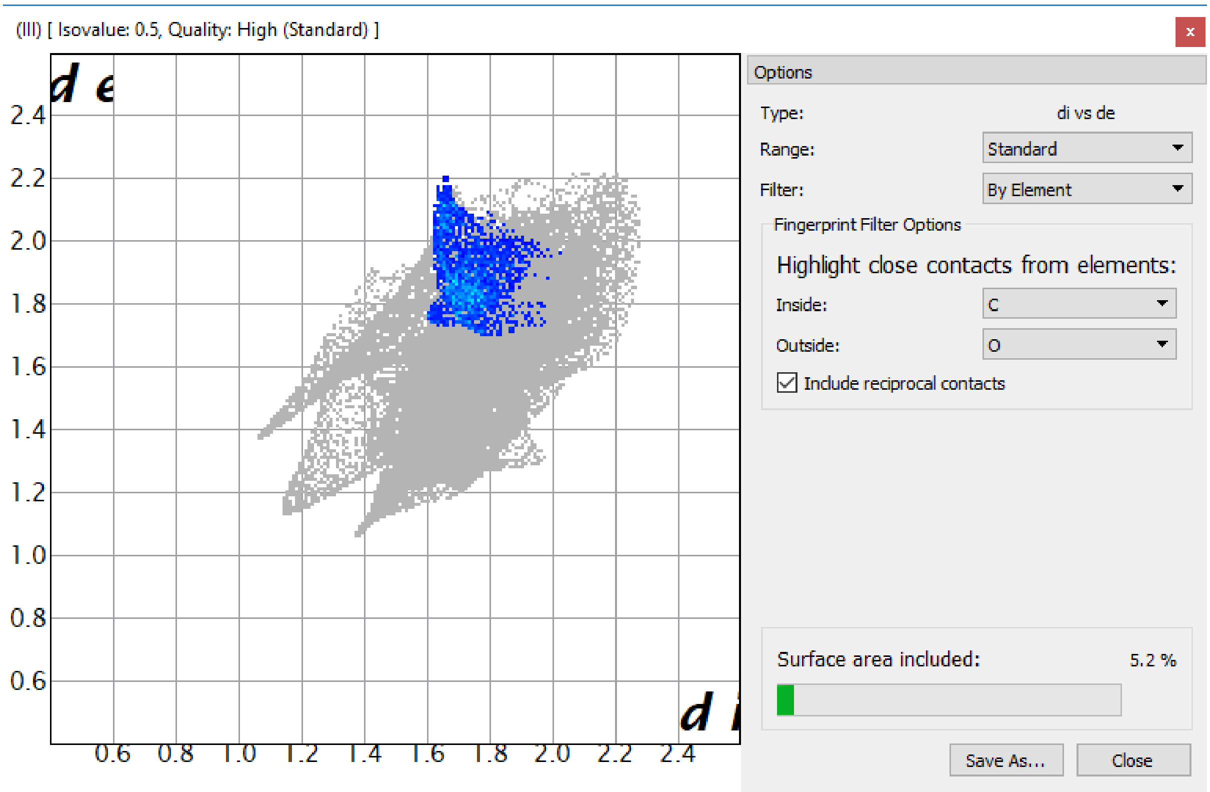
(III) [Isovalue: 0.5, Quality: High (Standard)]
d e
2.4
2.2
2.0
1.8
1.6
1.4
1.2
1.0
0.8
0.6
d i
0.6 0.8 1.0 1.2 1.4 1.6 1.8 2.0 2.2 2.4
Options
Type: di vs de
Range: Standard
Filter: By Element
Fingerprint Filter Options
Highlight close contacts from elements:
Inside: C
Outside: O
Include reciprocal contacts
Surface area included: 5.2 %
Save As...
Close

H-O: 56.8 %

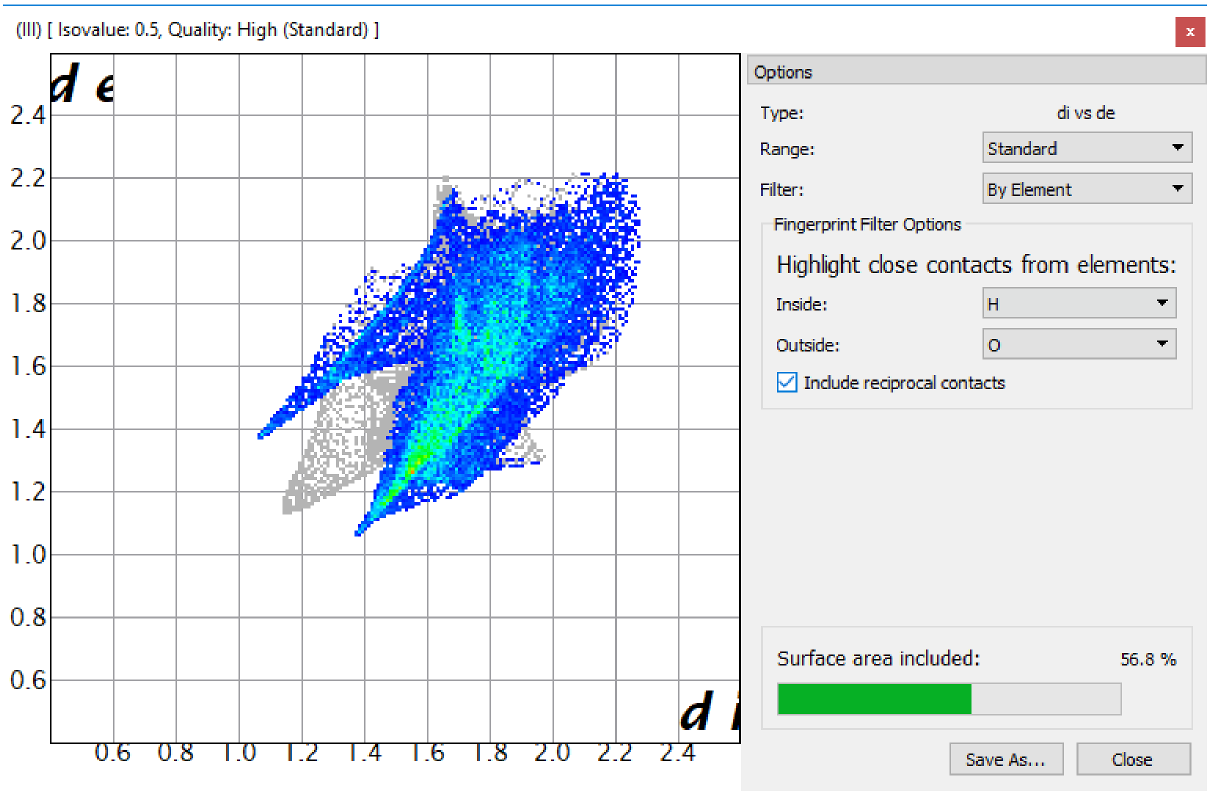
(III) [Isovalue: 0.5, Quality: High (Standard)]
d e
2.4
2.2
2.0
1.8
1.6
1.4
1.2
1.0
0.8
0.6
d i
0.6 0.8 1.0 1.2 1.4 1.6 1.8 2.0 2.2 2.4
Options
Type: di vs de
Range: Standard
Filter: By Element
Fingerprint Filter Options
Highlight close contacts from elements:
Inside: H
Outside: O
Include reciprocal contacts
Surface area included: 56.8 %
Save As...
Close

(IV)

ALL 100%

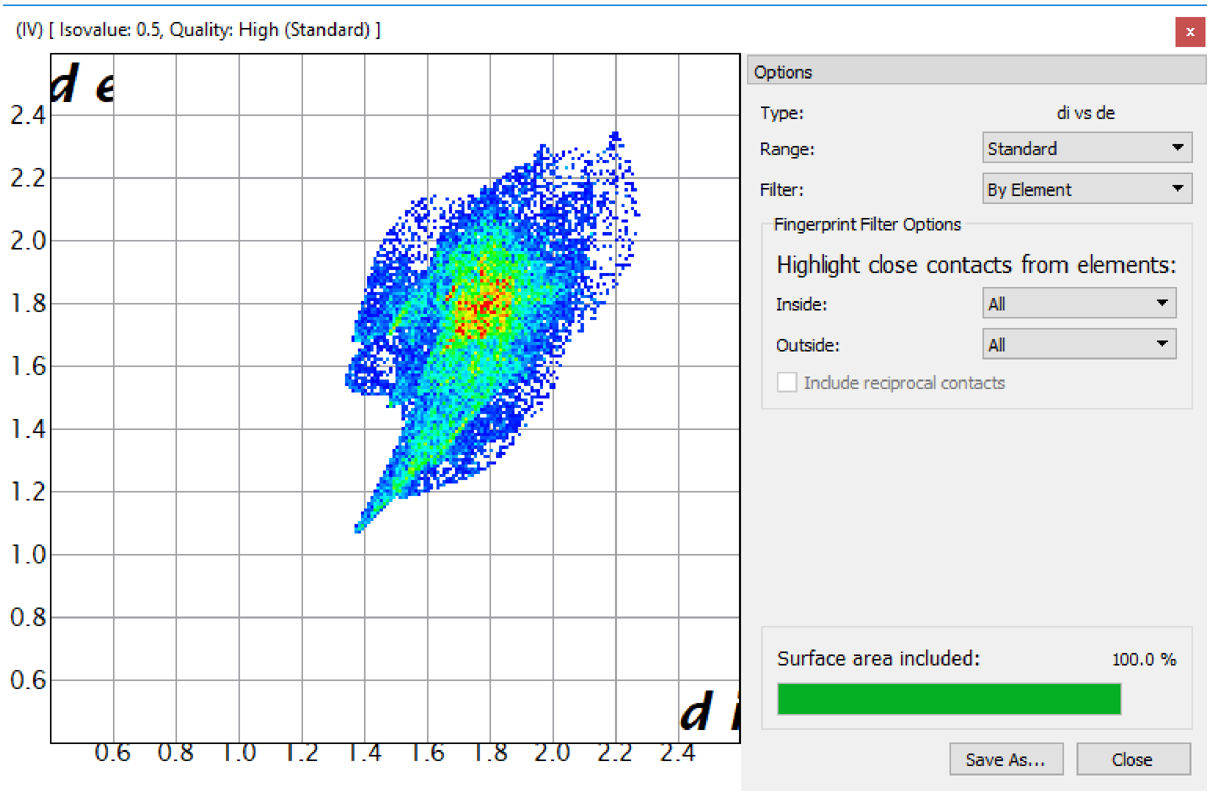
(IV) [Isovalue: 0.5, Quality: High (Standard)]
d e
d i
2.4
2.2
2.0
1.8
1.6
1.4
1.2
1.0
0.8
0.6
0.6 0.8 1.0 1.2 1.4 1.6 1.8 2.0 2.2 2.4
Options
Type: di vs de
Range: Standard
Filter: By Element
Fingerprint Filter Options
Highlight close contacts from elements:
Inside: All
Outside: All
Include reciprocal contacts
Surface area included: 100.0 %
Save As...
Close

C-C: 20.9 %

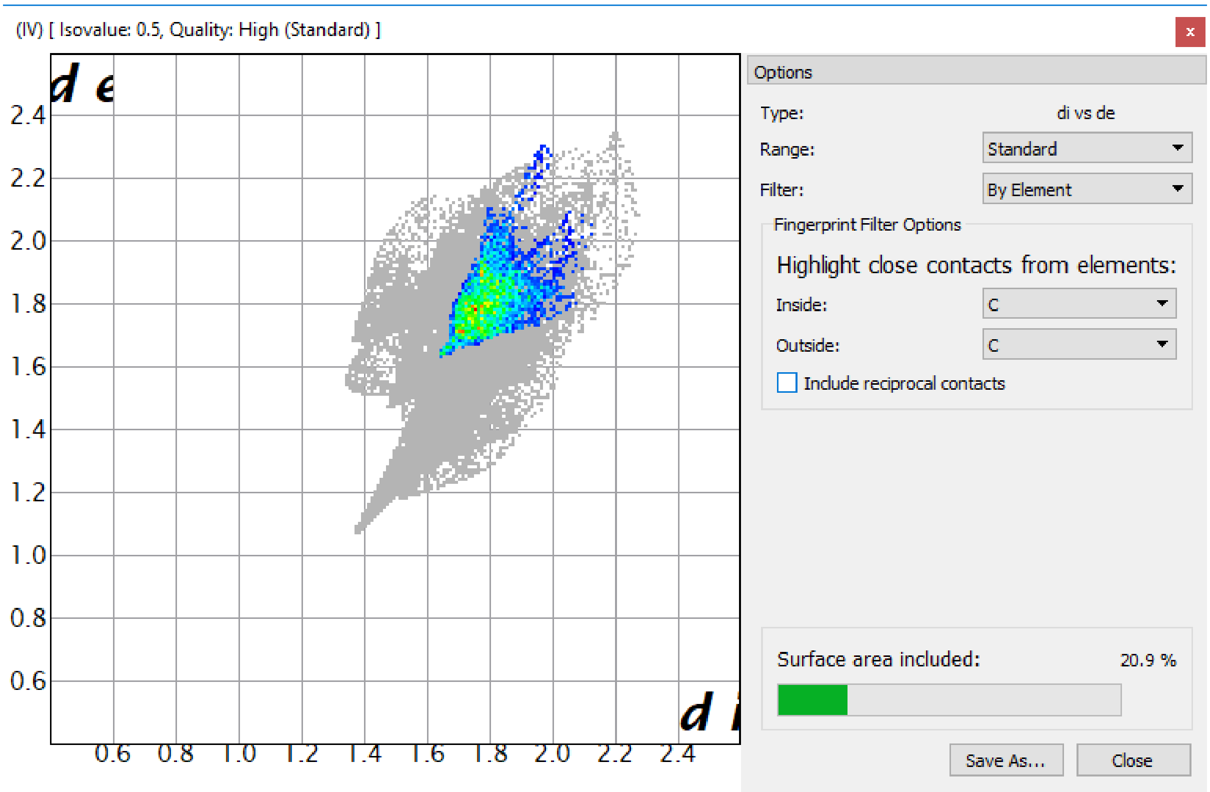
(IV) [Isovalue: 0.5, Quality: High (Standard)]
d e
2.4
2.2
2.0
1.8
1.6
1.4
1.2
1.0
0.8
0.6
d i
0.6 0.8 1.0 1.2 1.4 1.6 1.8 2.0 2.2 2.4
Options
Type: di vs de
Range: Standard
Filter: By Element
Fingerprint Filter Options
Highlight close contacts from elements:
Inside: C
Outside: C
Include reciprocal contacts
Surface area included: 20.9 %
Save As...
Close

H-H: 10.8 %

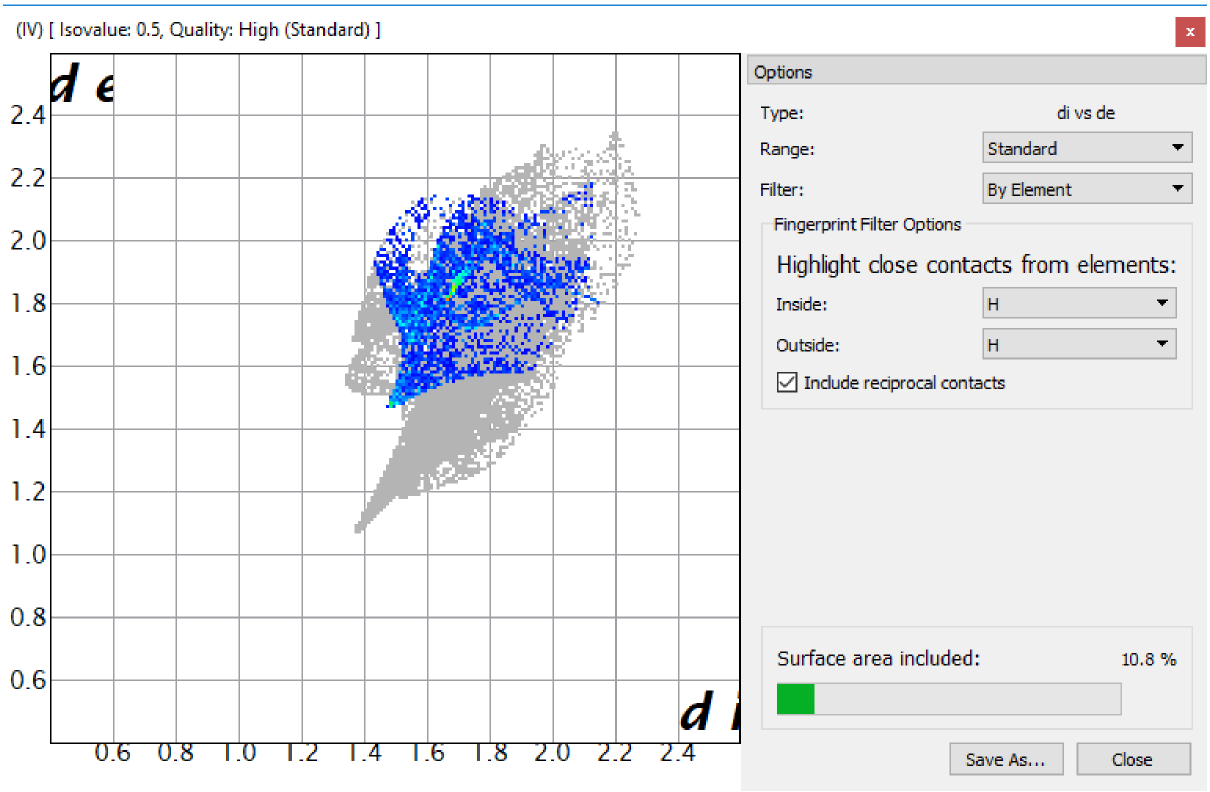
(IV) [Isovalue: 0.5, Quality: High (Standard)]
d e
2.4
2.2
2.0
1.8
1.6
1.4
1.2
1.0
0.8
0.6
d i
0.6 0.8 1.0 1.2 1.4 1.6 1.8 2.0 2.2 2.4
Options
Type: di vs de
Range: Standard
Filter: By Element
Fingerprint Filter Options
Highlight close contacts from elements:
Inside: H
Outside: H
Include reciprocal contacts
Surface area included: 10.8 %
Save As...
Close

O-O: 4.4 %

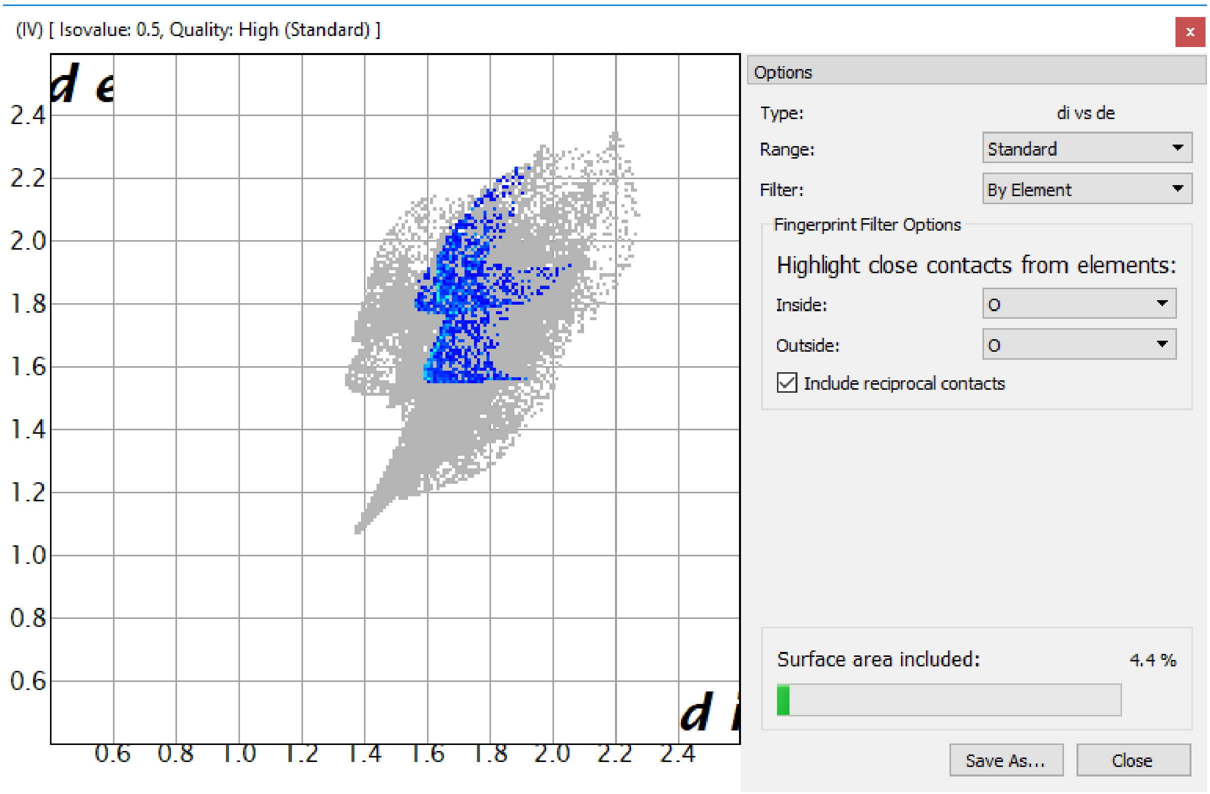
(IV) [Isovalue: 0.5, Quality: High (Standard)]
d e
2.4
2.2
2.0
1.8
1.6
1.4
1.2
1.0
0.8
0.6
d i
0.6 0.8 1.0 1.2 1.4 1.6 1.8 2.0 2.2 2.4
Options
Type: di vs de
Range: Standard
Filter: By Element
Fingerprint Filter Options
Highlight close contacts from elements:
Inside: O
Outside: O
Include reciprocal contacts
Surface area included: 4.4 %
Save As...
Close

C-H: 2.7 %

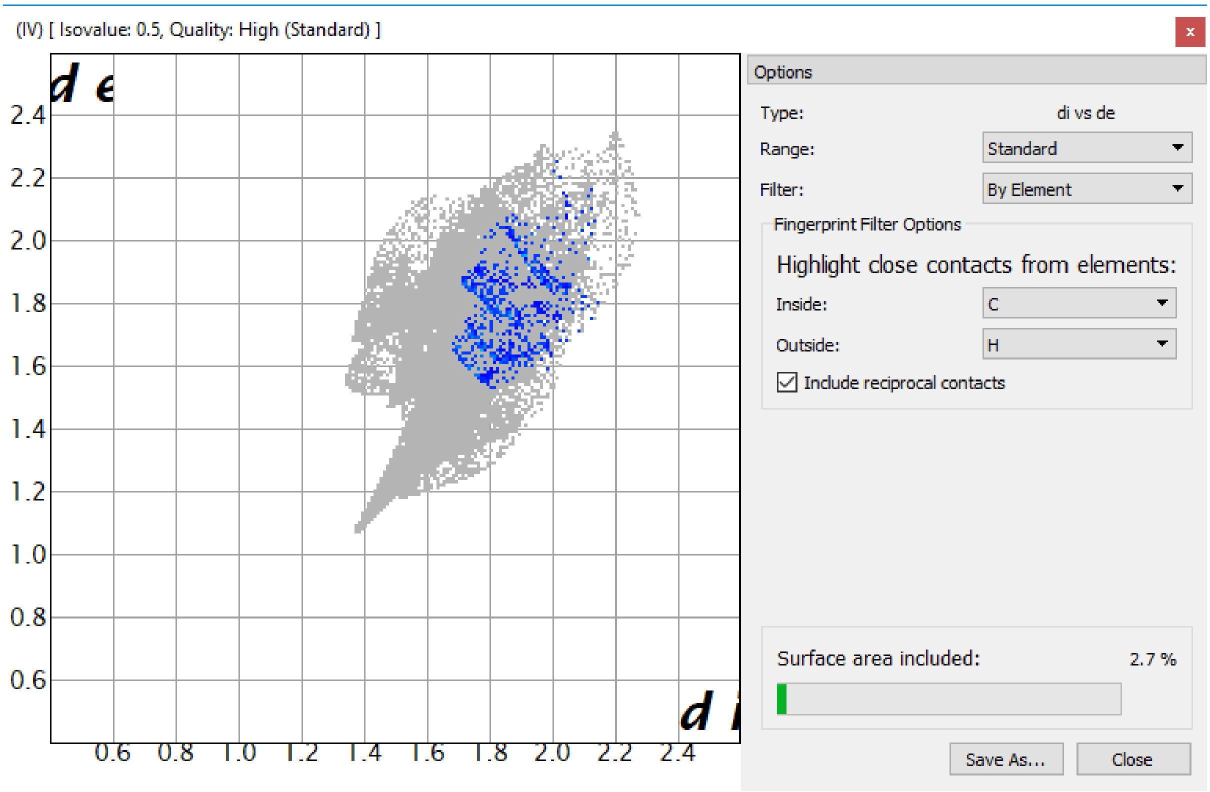
(IV) [Isovalue: 0.5, Quality: High (Standard)]
d e
2.4
2.2
2.0
1.8
1.6
1.4
1.2
1.0
0.8
0.6
d i
0.6 0.8 1.0 1.2 1.4 1.6 1.8 2.0 2.2 2.4
Options
Type: di vs de
Range: Standard
Filter: By Element
Fingerprint Filter Options
Highlight close contacts from elements:
Inside: C
Outside: H
Include reciprocal contacts
Surface area included: 2.7 %
Save As...
Close

C-O: 7.3 %

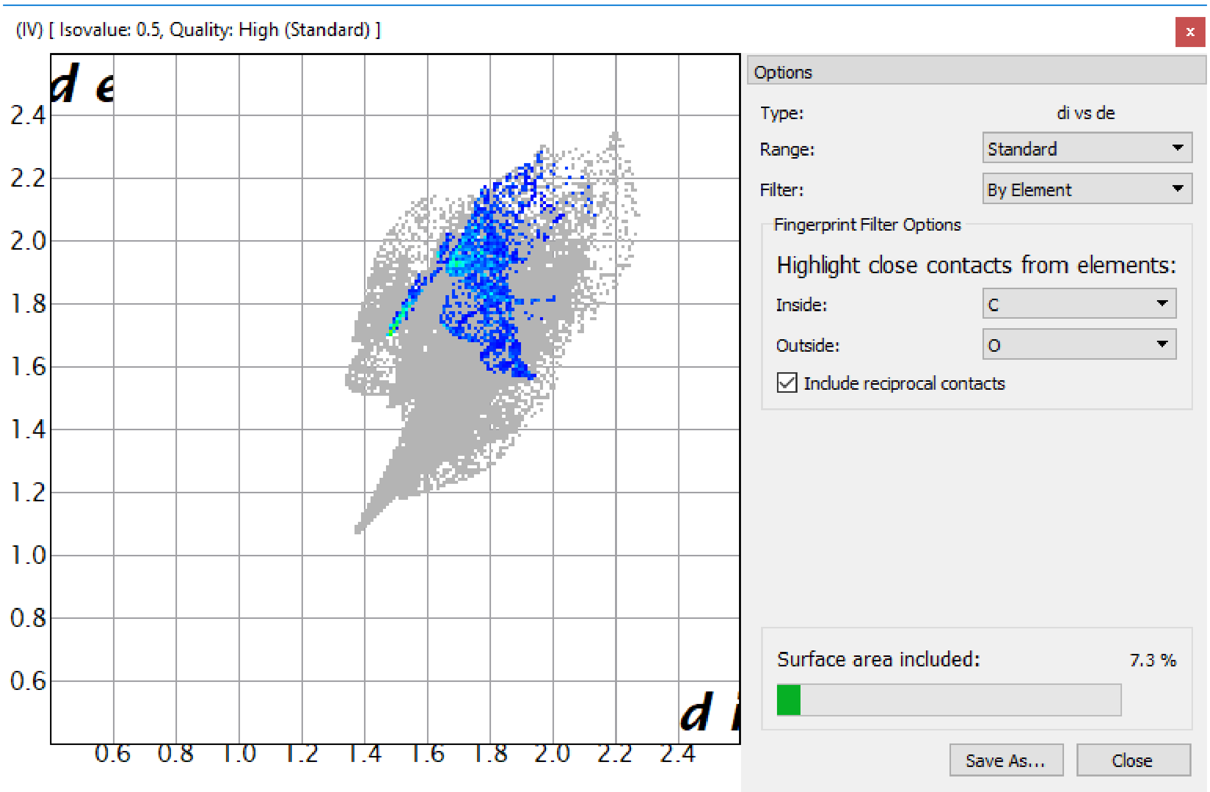
(IV) [Isovalue: 0.5, Quality: High (Standard)]
d e
2.4
2.2
2.0
1.8
1.6
1.4
1.2
1.0
0.8
0.6
d i
0.6 0.8 1.0 1.2 1.4 1.6 1.8 2.0 2.2 2.4
Options
Type: di vs de
Range: Standard
Filter: By Element
Fingerprint Filter Options
Highlight close contacts from elements:
Inside: C
Outside: O
Include reciprocal contacts
Surface area included: 7.3 %
Save As...
Close

O-H: 53.9 %

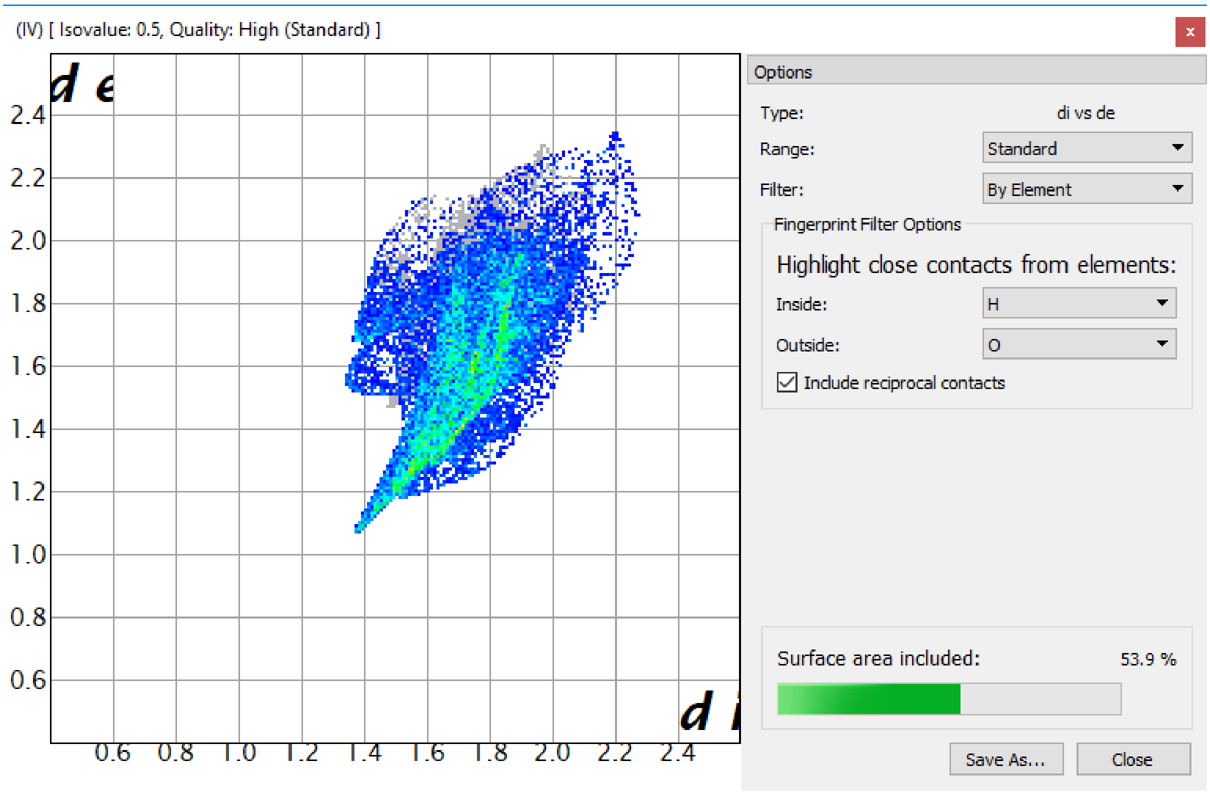
(IV) [Isovalue: 0.5, Quality: High (Standard)]
d e
2.4
2.2
2.0
1.8
1.6
1.4
1.2
1.0
0.8
0.6
d i
0.6 0.8 1.0 1.2 1.4 1.6 1.8 2.0 2.2 2.4
Options
Type: di vs de
Range: Standard
Filter: By Element
Fingerprint Filter Options
Highlight close contacts from elements:
Inside: H
Outside: O
Include reciprocal contacts
Surface area included: 53.9 %
Save As...
Close
